# Supplementary material for: TRACERx analysis identifies a role for FAT1 in regulating chromosomal instability and whole-genome doubling via Hippo signalling
Source: Nat Cell Biol. 2024 Dec 30;27(1):154–68. doi: 10.1038/s41556-024-01558-w (PMC11735399; doi:10.1038/s41556-024-01558-w)
Supplement: Supplementary file 1 — Supplementary Figs. 1–10, uncropped blots for Supplementary Figs. 2 and 6, Supplementary Methods, full plasmid maps and sequences. [file 41556_2024_1558_MOESM1_ESM.pdf]

nature cell biology

Supplementary information https://doi.org/10.1038/s41556-024-01558-w

TRACERx analysis identifies a role for *FAT1* in regulating chromosomal instability and whole-genome doubling via Hippo signalling

In the format provided by the authors and unedited

Supplementary figure 1 String functional analysis – TRACERx100 non-oncogenic drivers co-occuring with genome doubling.

Genes reported to contribute to DDR

Chromatin remodelers or transcription factors.

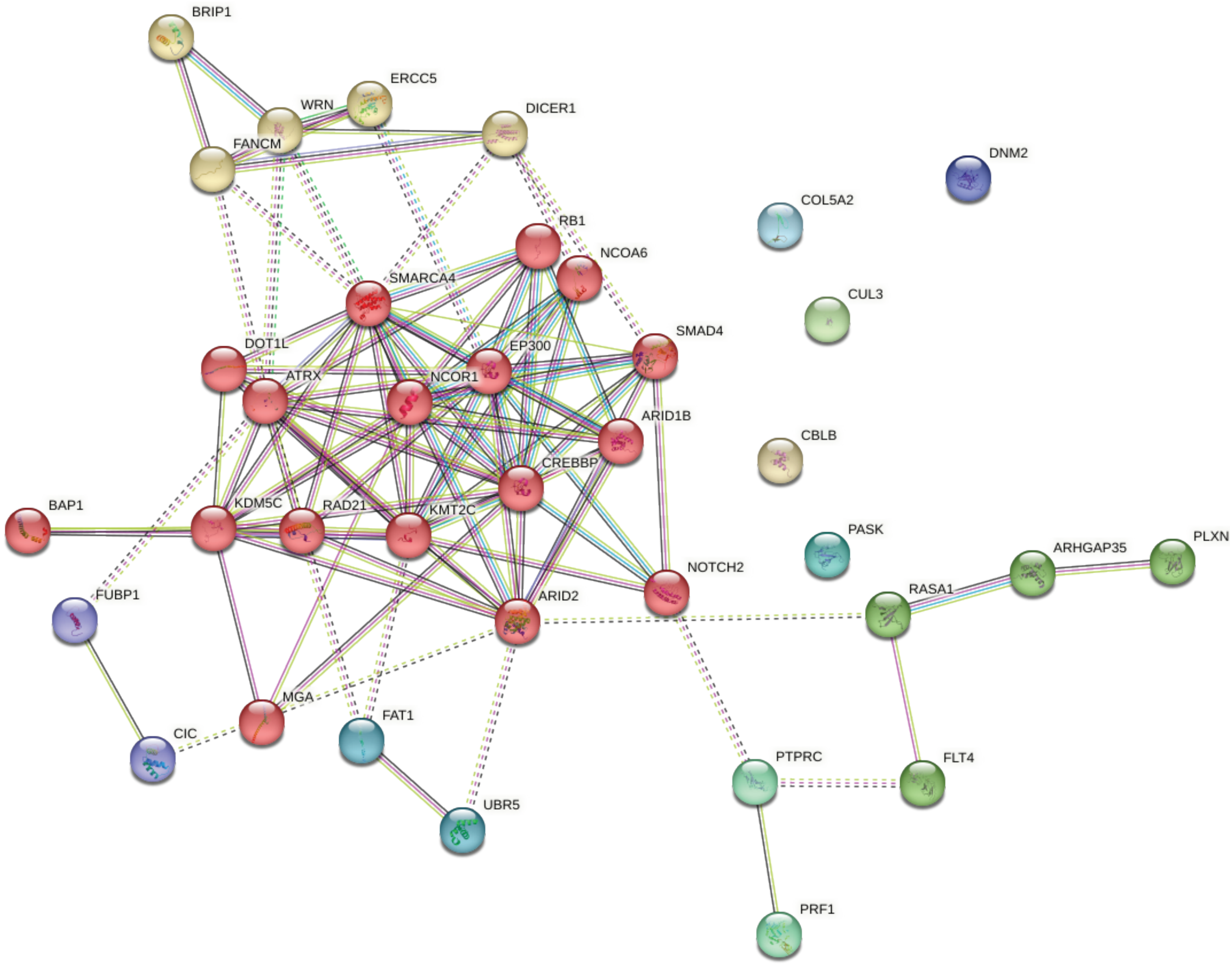

Supplementary figure 2: Representative western blots showing the efficiency of RNAi depletion of the 6 candidate genes in the U2OS-AsiSI site-directed resection assay.

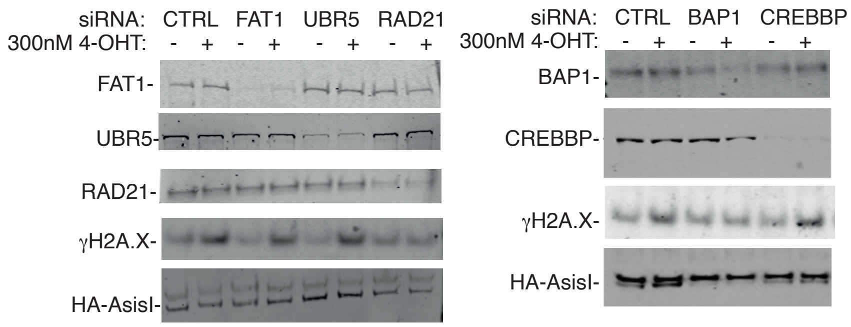

Supplementary figure 3. Representative figures illustrating RAD51/γH2A.X foci in the presence of 6Gy IR. A549 cellswere irradiated and allowed 1h recovery. Cells were counterstained with DAPI.White arrows = zoomed-in cells. Scale bar =10 μm

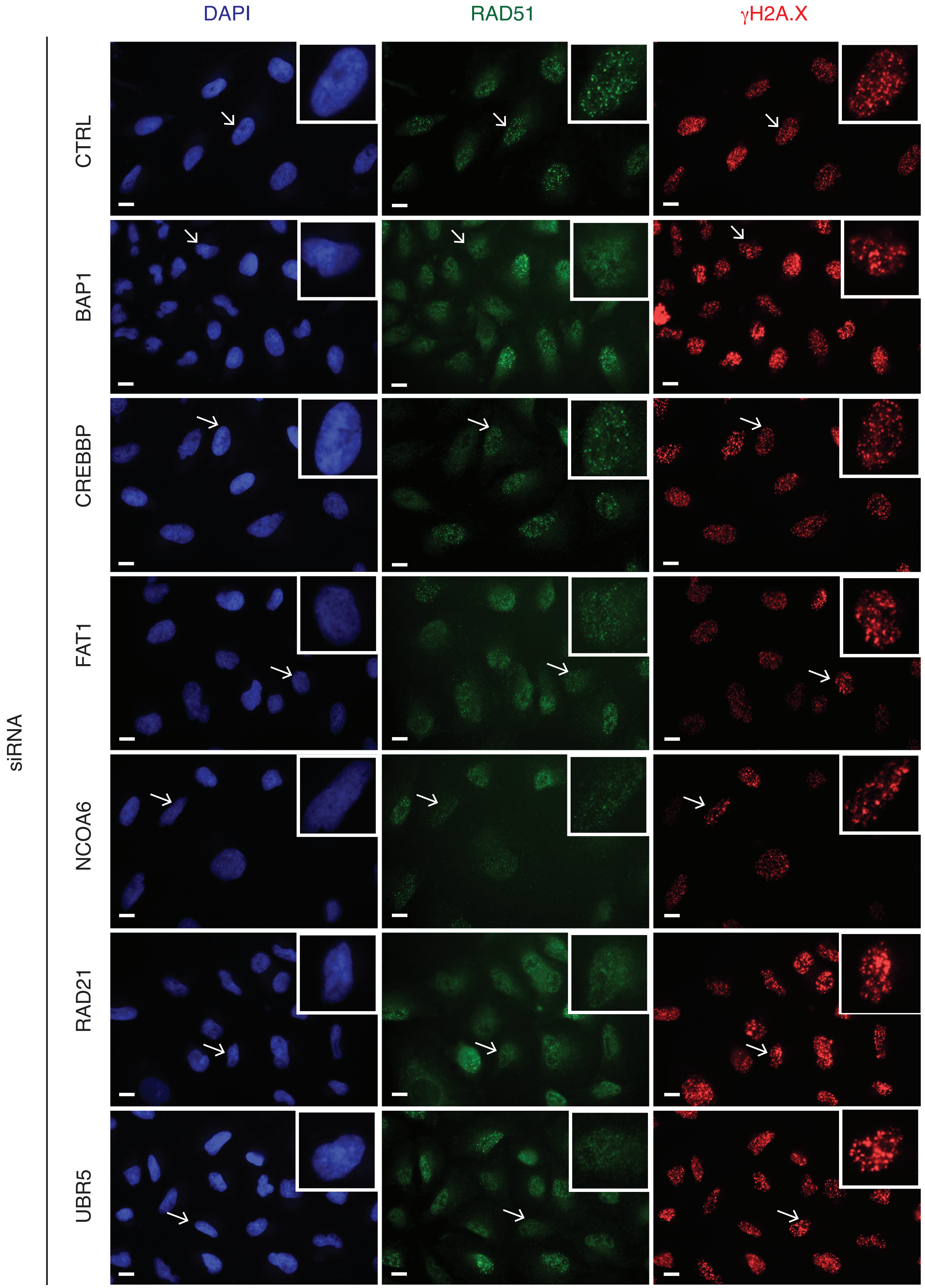

Supplementary figure 4: Representative figures and box plots illustrating RAD51 foci in the presence and absence of 6Gy IR. H1944 cells were irradiated and allowed 1h recovery. RAD51 in green and γH2A.X foci in red. Cells were counterstained with DAPI. White arrows=RAD51 positive cells. Dunn's test, ***p<0.001. Scale bar =10 μm

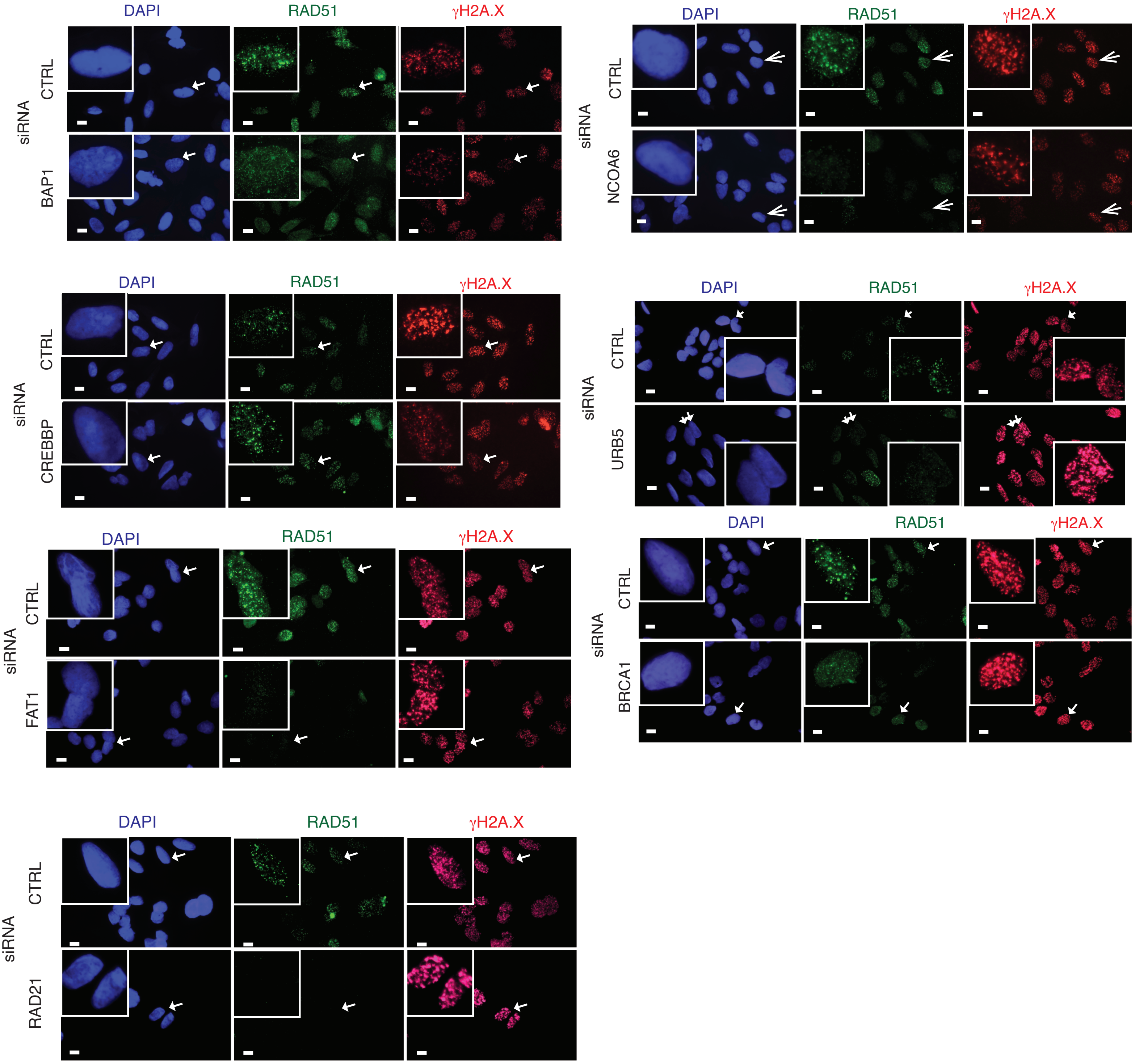

Supplementary figure 5A, Breakdown of clonal mutation occurrence among the 6 candidate DDR+CIN genes in the TRACERx421 cohort. *FAT1* clonal mutations occur frequently, particularly in LUSC tumors. **B,** Detail breakdown of clonal mutation occurrence among the 6 candidate DDR+CIN genes in the TRACERx421 cohort in LUAD tumors, stratified by stages. **C,** Breakdown of clonal mutation occurrence among the 6 candidate genes in the TRACERx421 cohort in LUSC tumors by stage. **D**, Driver mutation distribution of *FAT1*, and HR-related genes (*ATM-CHEK2, ATR-CHEK1*, and members of *FA/BRCA* pathway) in the TRACERx 421 cohort

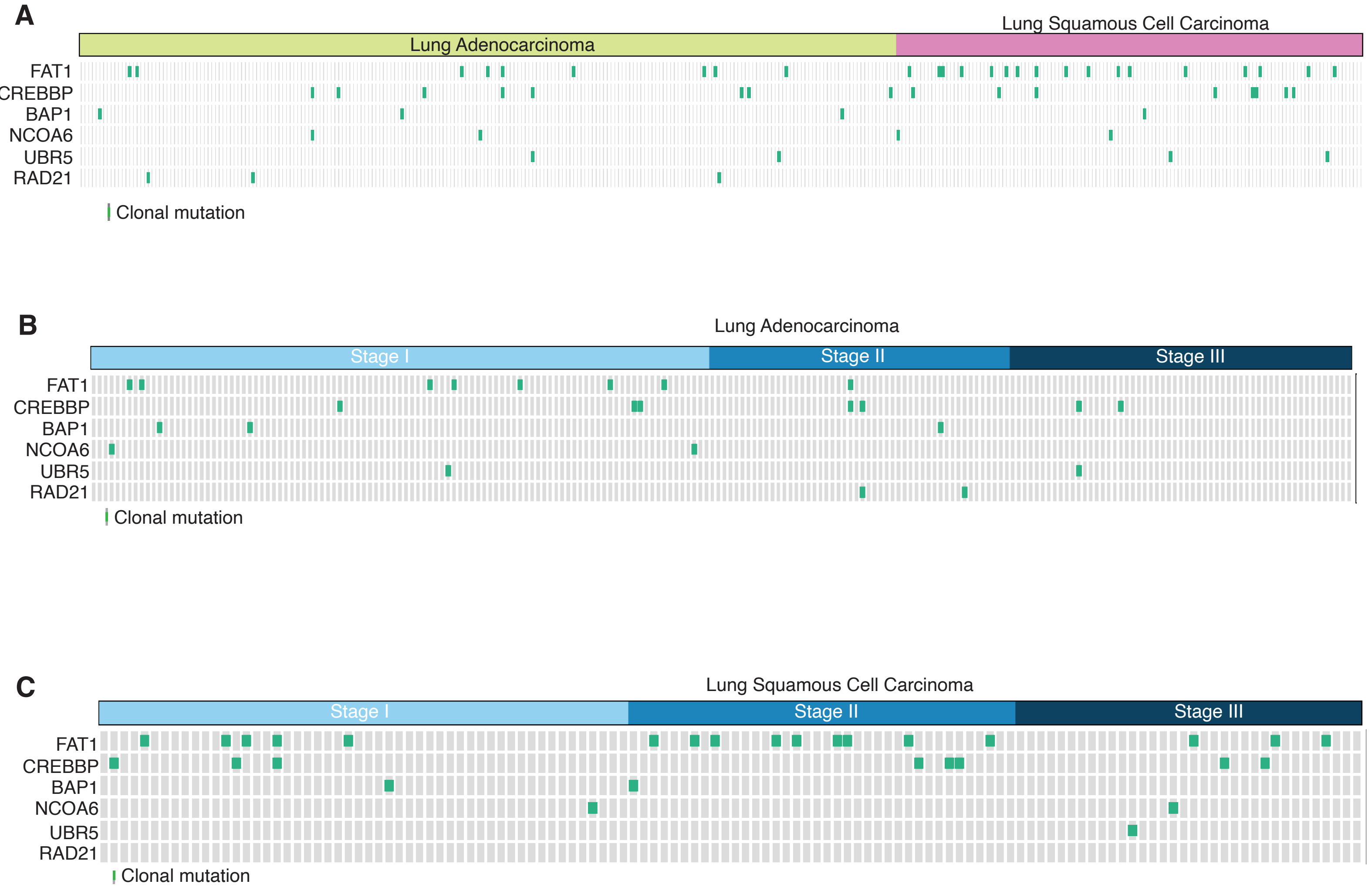

Supplementary figure 6A: Representative figures showing phospho-ATM S1981 foci in the presence of 6Gy IR. A549 cells were irradiated and allowed 1h recovery. Cells were counterstained with DAPI. Scale bar =10 μM. **B,C**: Representative figures showing 53BP1/γH2A.X foci (B) and CtIP/γH2A.X foci (C) in the presence of 6Gy IR. A549 cell were irradiated and allowed 1h recovery. Cells are counterstained with DAPI. n=3, scale bar =10 μM **D**: Representative figures illustrating BRCA1 foci in the presence of 6Gy IR. A549 cell were irradiated and allowed 1h recovery. Cells were counterstained with CENP-F to identify G2/M cells. Scale bar =10 μm. **E**: Representative western blot showing that following the knockout of *FAT1*, the level of KAP1/TRIM28 phosphorylation, γH2A.X, ubiquitination of γH2A.X, and phosphorylation of Chk2 at Thr68 are not affected.

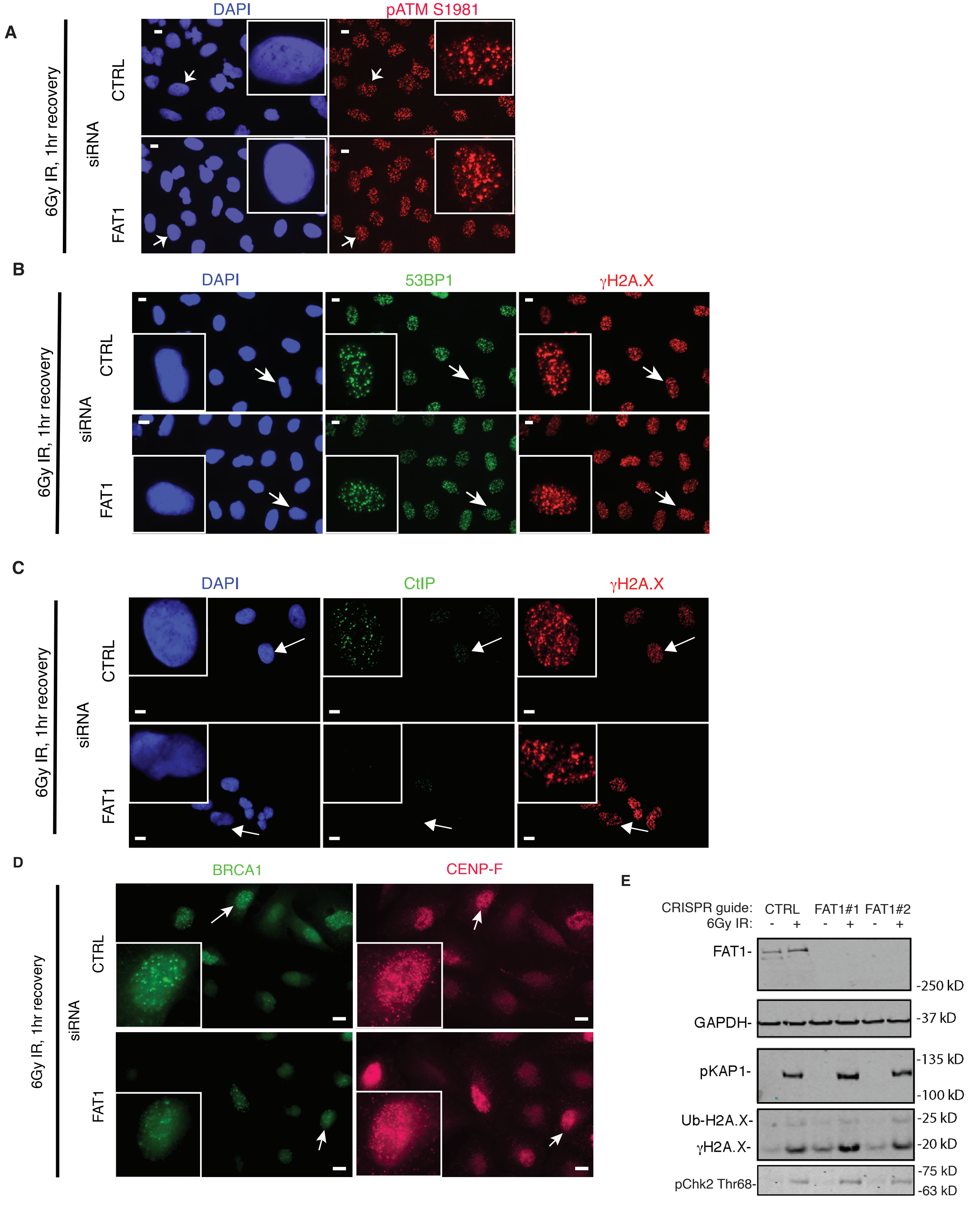

Supplementary figure 7A-B, Mutational profile cartoon showing the mutational profile of COSMIC ID6 (A) and SBS3 (B) mutational signatures, both dependent on indels as a result of end-joining activity.

A

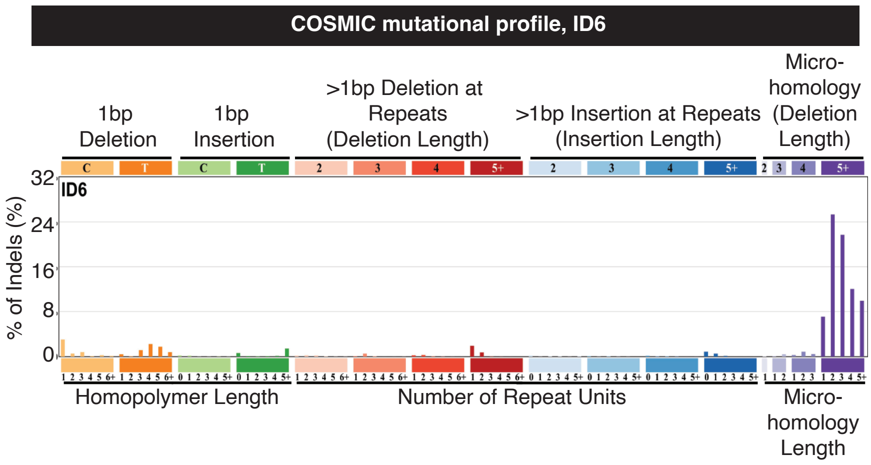

B

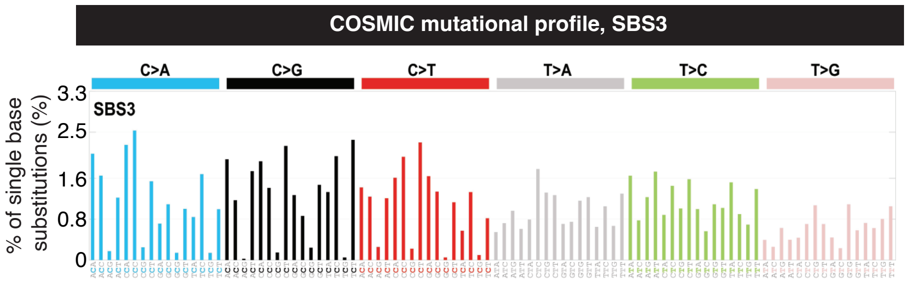

Supplementary Figure 8, Representative images of a normal mitosis versus a mitotic bypass event in RPE1-TERT-FUCCI in a live cell tracking experiment. A cell exhibiting prolonged mVenus-Gemini expressing G2 phase can be observed.

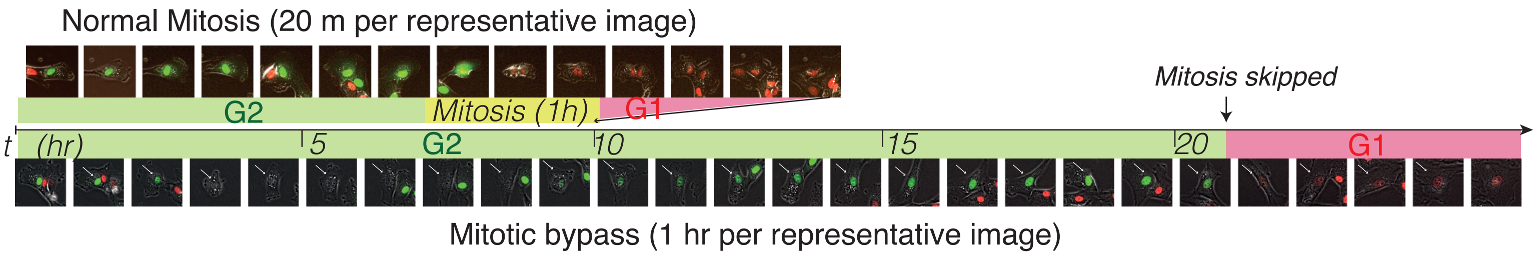

Supplementary figure 9, Representative immunofluorescence images illustrating the overexpression rate and subcellular localization of the HA-FAT1^{WT} construct versus the HA-FAT1^{ICD} construct.

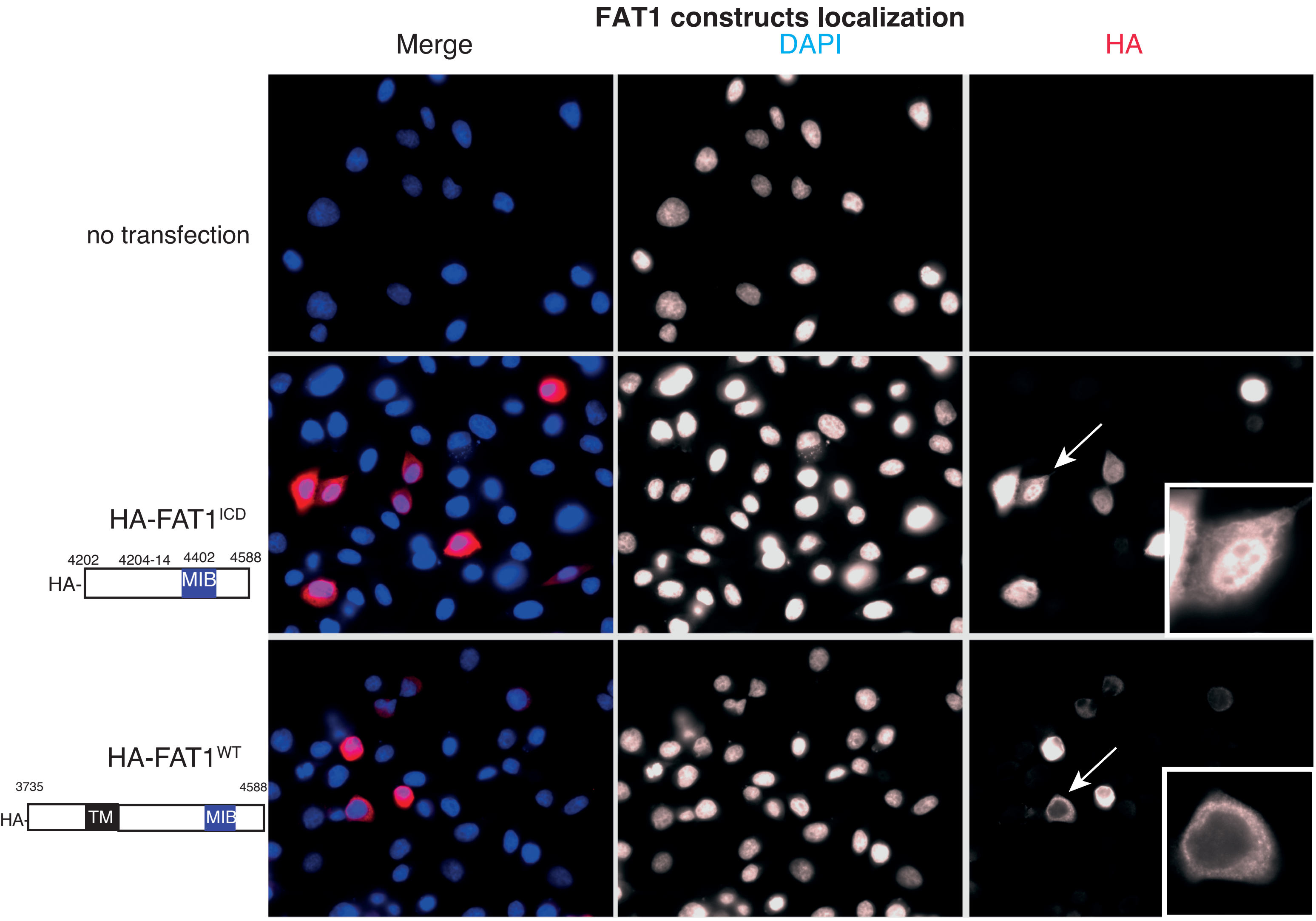

Supplementary figure10 A-B, Representative dot plot showing (A) selection of mScarlet-YAP^{5SA} subpopulation for flow cytometry analysis; and (B) EdU incorporation assay used to determine actively replicating WGD cells.

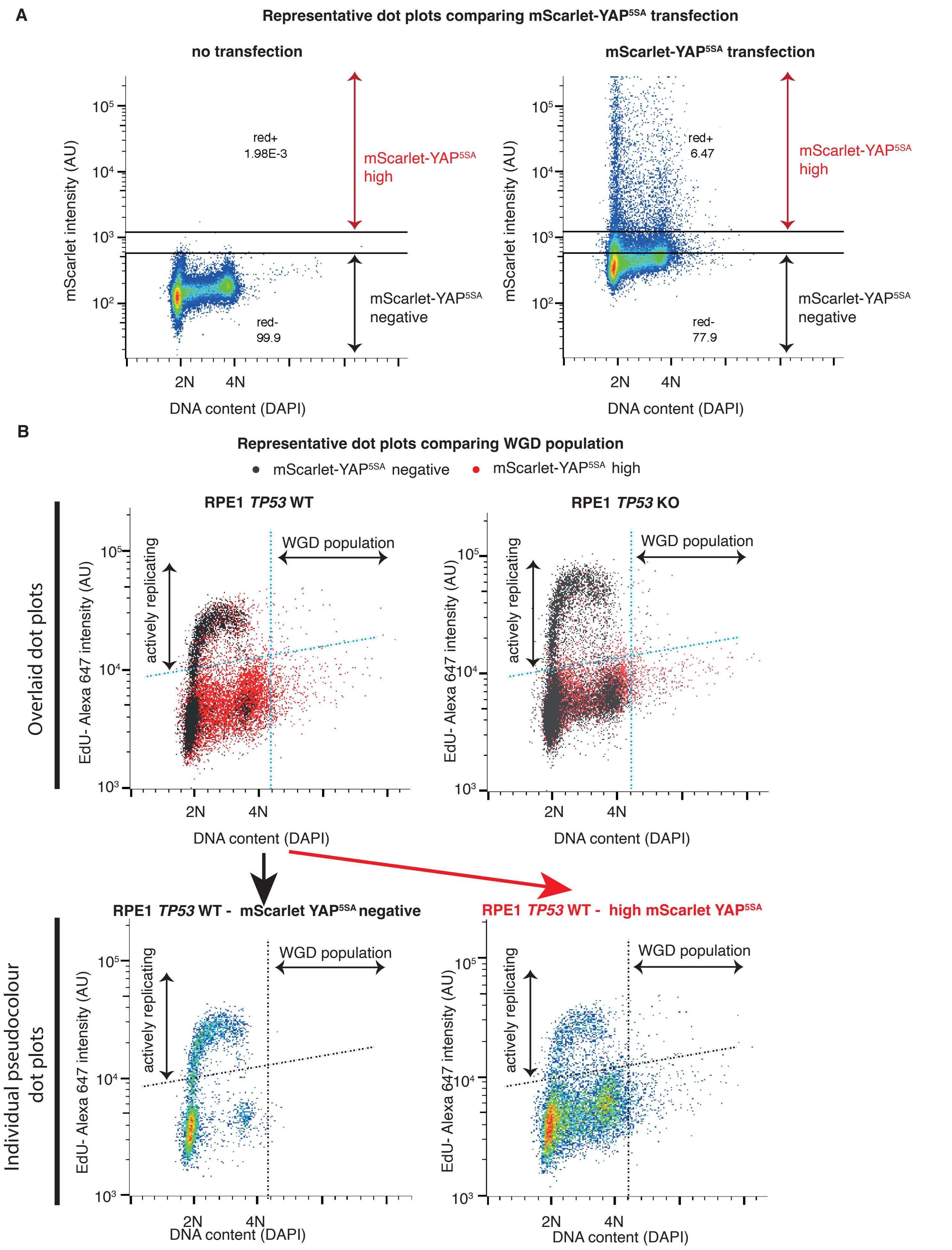

Uncropped WB for Supplementary Fig 2:

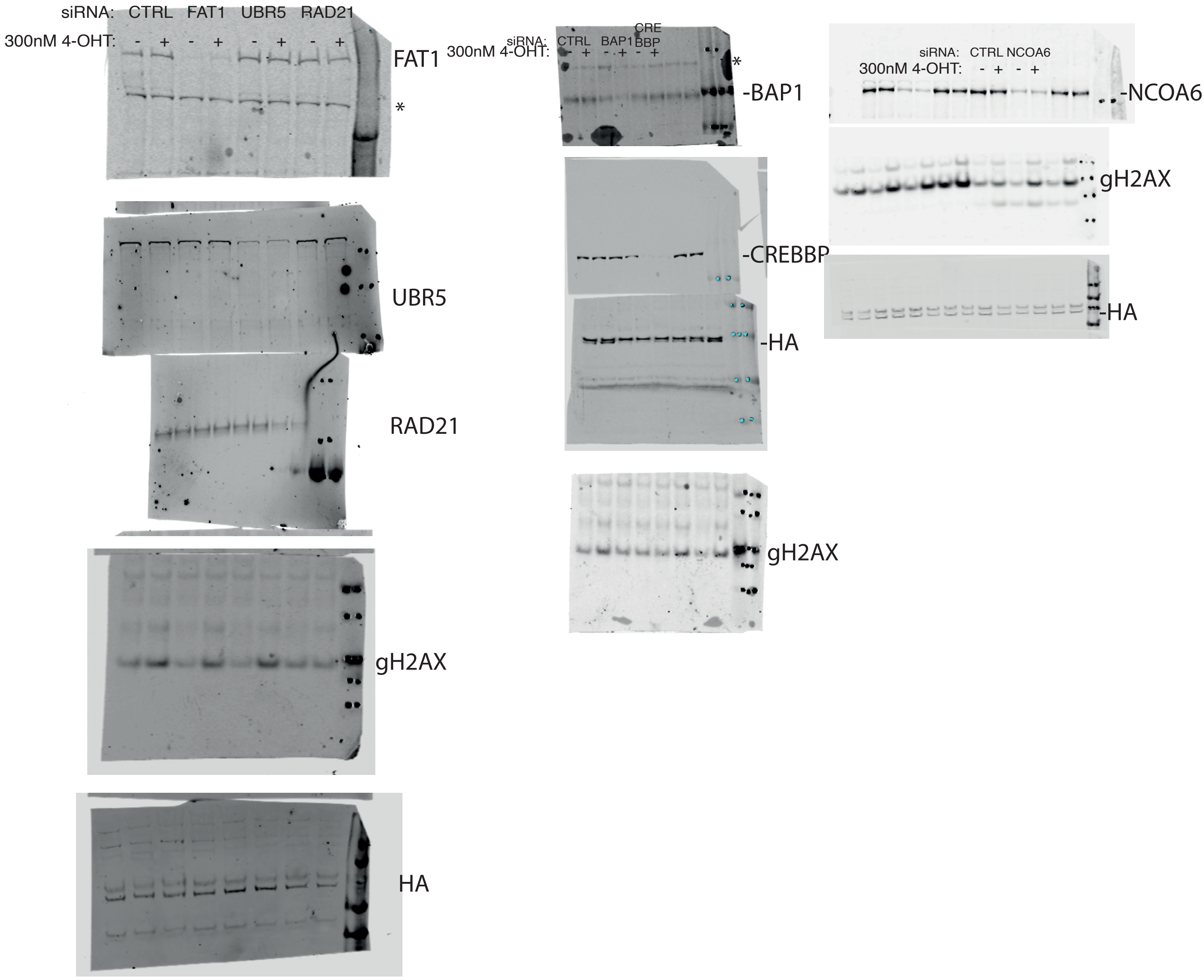

uncropped WB for Supplementary Fig 6E

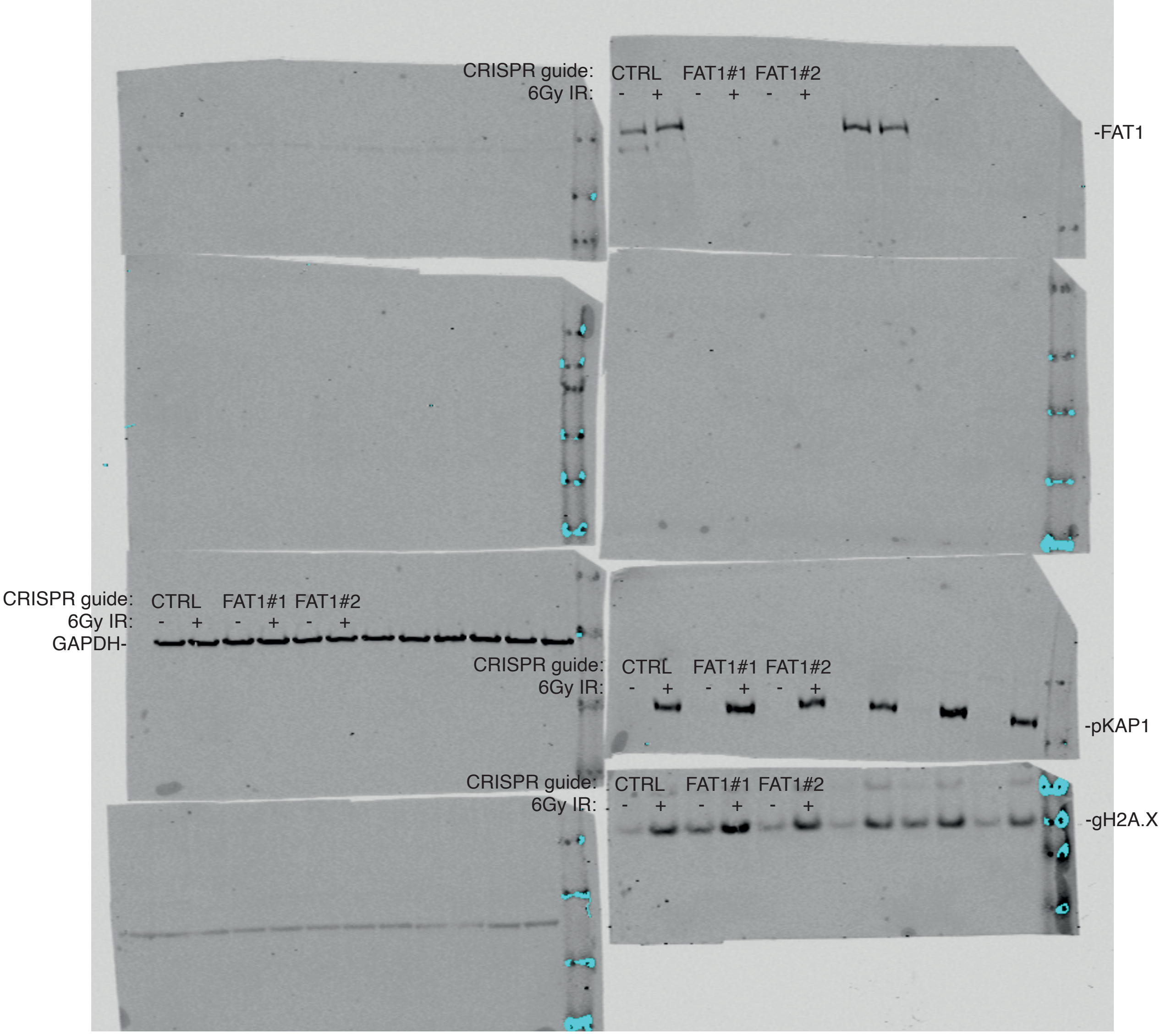

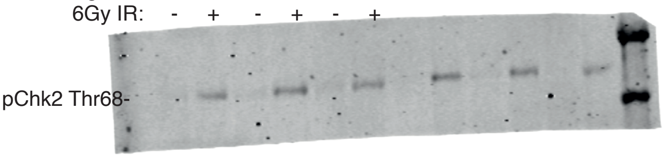

Supplementary methods

Western Blotting, subcellular fractionation, and antibodies

Whole-cell protein lysates were harvested by scrapping in Urea buffer (9 M Urea, 50 mM Tris-HCl pH7.5, 150 mM β-mercaptoethanol, protease and phosphatase inhibitor tablets). DNA was sheared by passing the lysate through a 23-gauge needle 10 times. Proteins were calibrated by Bradford assay and denatured with Laemmli buffer. IR-Dye labelled secondary antibodies (Li-COR Biosciences) were used and blots were scanned using a Li-COR Odyssey CLx scanner.

For subcellular fractionation, the cytoplasmic fraction was extracted on ice for 8 minutes using cytoplasmic buffer (10 mM HEPES, pH 7.9, 10 mM KCl, 1.5 mM $MgCl_2$, 0.34 M Sucrose,10 % Glycerol,1 mM DTT, Protease inhibitor cocktail, 10mM β-glycerol phosphate, 50mM NaF, 0.1% Triton X-100). The supernatant was obtained at 1,300 g, and pellet lysed with CSK buffer (10mM PIPES pH6.8, 100mM NaCl, 300 mM sucrose, 3 mM $MgCl_2$,1 mM EDTA, 0.4% Triton X-100, 1 mM DTT, protease inhibitor cocktail, 10mM β-glycerol phosphate) for 30 minutes. Lysates were centrifuged at 1,700 g, and the supernatant was designated as the nuclear soluble fraction. The pellet containing chromatin fraction was extracted with urea buffer (9 M Urea, 50 mM Tris-HCl pH7.5, 150 mM β-mercaptoethanol, protease and phosphatase inhibitor tablets). DNA was sheared by passing the lysate through a 23-gauge needle 10 times. Equal volumes of samples were loaded for each fraction.

All antibodies used in this study are listed in supplementary table 1.

High content immunofluorescence screen for altered DNA damage response

Cell preparation

Cells were pre-seeded 72 hours prior to siRNA transfection and 2.5 µL of 400 nM SMARTPool siRNAs (Dharmacon) were stamped onto 384-well plates. On the day of transfection, 2.5 µL/well of OptiMEM (Gibco) containing 0.1 µL of Lullaby reagent was added to the siRNA-containing wells and incubated for 20 minutes. Cells were resuspended in complete culture media and 25 x 10^6 H1944 or HCC4006 cells, and 21 x 10^6 H1792 or H1650 cells were each resuspended in 750 mL media. 45 µL of cell mixture containing either 1500 cells (H1944, HCC4006) or 1250 cells (H1792 and H1650) was added to the siRNA/lullaby mixes in the 384 plates. Cells were incubated for 72 hours before harvesting at either 72 hours (untreated), 3 hours after irradiation, 24 hours after irradiation, or 24 hours after 2mM Hydroxyurea treatment.

Irradiation treatment

Cells were irradiated in a Caesium source irradiator at room temperature with 6 Grays at a rate of ~1 Gray/minute (363 seconds).

Hydroxyurea treatment

Cells were incubated in 2mM hydroxyurea for 24 hours.

Fixation

30µL PTEMF (20mM PIPES pH6.8, 10mM EGTA, 0.2% TritonX-100, 1mM $MgCl_2$, 4%PFA) was added into every well for 22 minutes followed by washing 3 times with PBS.

Antibody incubation

Following blocking with 30µL blocking buffer (3% BSA, 0.2% triton, PBS, filter sterilised), 18 µL primary antibody mix was dispensed to assay wells. Plates were incubated at 4°C overnight in a humidified sealed box. Plates were washed 3 times with PBS and 18 µL secondary antibody mix supplemented 1 µg/mL DAPI diluted in IF antibody buffer (3% BSA, 0.1% Triton, PBS, filter sterilised) was added. Plates were incubated for 2 hours at room temperature in the dark before washing 3 times and sealing for automated microscope imaging.

Plate imaging

Plates were scanned at 20X magnification using a Cellomics ArrayScan Vti microscope collecting 15 fields/well. Exposure settings were calibrated on a per-plate basis using software auto-exposure

settings applied to the positive control wells. The measurement readouts were generated using the 'Target Activation' algorithm settings within the Cellomics Bioapplication software. Spot-calling settings within this algorithm were calibrated based on the images of the positive and negative controls present for each cell line in each screen condition.

siRNA Screen Analysis

The siRNA screen data was used to classify hits based on random forest classifiers built to identify genes within the screens that had feature scores similar to controls. For simplicity, a screen refers to one experimental condition for the set of siRNA library plates in triplicate. So each cell line, condition and stain were considered separate screens (with γH2A.X being the third stain in all screens). There were 48 individual screens in total.

36 different measurements were extracted from the Cellomics ArrayScan image analysis software as follows:
MEAN_ObjectSpotTotalCountCh2
MEAN_ObjectSpotTotalAreaCh2
MEAN_ObjectSpotAvgAreaCh2
MEAN_ObjectSpotTotalIntenCh2
MEAN_ObjectSpotAvgIntenCh2
MEAN_ObjectSpotTotalCountCh3
MEAN_ObjectSpotTotalAreaCh3
MEAN_ObjectSpotAvgAreaCh3
MEAN_ObjectSpotTotalIntenCh3
MEAN_ObjectSpotAvgIntenCh3
MEAN_Circ_TotalIntenCh2
MEAN_Circ_TotalIntenCh3
MEAN_Circ_AvgIntenCh2
MEAN_Circ_AvgIntenCh3
percentHIGH_Circ_TotalIntenCh2
percentLOW_Circ_TotalIntenCh2
percentHIGH_Circ_TotalIntenCh3
percentLOW_Circ_TotalIntenCh3
percentHIGH_Circ_AvgIntenCh2
percentLOW_Circ_AvgIntenCh2
percentHIGH_Circ_AvgIntenCh3
percentLOW_Circ_AvgIntenCh3
percentHIGH_SpotAvgAreaCh2
percentLOW_SpotAvgAreaCh2
percentHIGH_SpotAvgAreaCh3
percentHIGH_SpotAvgIntenCh2
percentLOW_SpotAvgIntenCh2
percentHIGH_SpotAvgIntenCh3
percentLOW_SpotAvgIntenCh3
percentHIGH_SpotCountCh2
percentHIGH_SpotCountCh3
percentLOW_SpotCountCh3
percentHIGH_SpotTotalAreaCh2
percentHIGH_SpotTotalAreaCh3
percentHIGH_SpotTotalIntenCh2
percentHIGH_SpotTotalIntenCh3

Where Ch2 measurements refer to γH2AX in every screen, and Ch3 measurements correspond to the specific stain within the screen. A random forest model was built for each screen with the Ch3 measurements. Ch2 measurements were then grouped across several screens (combining screens with the same condition/cell line as γH2AX replicates). Raw, un-normalised data was used.

To define which controls to use to train the random forest models, the separation of controls in each screen was investigated using principal component analysis. The first three components were used to calculate an Euclidean distance for each control well from the mean of the neutral control wells. A threshold for the neutral controls was taken as the 0.8 percentile distance of all neutral RISC-free control wells in the Ch3 measurement models, and 0.85 in the Ch2 γH2AX measurements (as there were more samples/replicates). This threshold was used to calculate the percentage of each control population that was above this threshold and therefore defined as separated from the RISC-free population of samples. Any control that had > 80% of its samples above the threshold was used to train the model. The details of the controls and their scores that went into each model are detailed in screen_modelScores.txt and screen_modelScores_H2AX.txt.

Each random forest model was built using 100 trees, with 2 variables randomly selected at each split using the R-package randomForest. All other default arguments were used for the randomForest function. 6 control wells for each control were randomly left out for validation of the models, with the remaining controls selected for training. With the Ch3 measurement models, 50 randomly selected RF and 50 randomly selected OTNT control wells, along with the selected control wells defined above were used to train the models. For the Ch2 γH2AX data, 80% of the RF and OTNT wells were used for training, together with the selected control wells. Model scores are outputted in the screen_modelScores.txt and screen_modelScores_H2AX.txt files.

The random forest models assigned each sample siRNA as well as a hit or non-hit neutral control based on the training as above. Within each screen, each siRNA was scored as a TRUE/FALSE hit if the random forest classified two or more replicate siRNAs as a hit. Heatmaps were produced with these binary classifications using the R package pheatmap, using hierarchical clustering based on both genes and conditions/cell lines and calculating distances between clusters using the Manhattan distance method.

Human artificial chromosome (HAC) assay for chromosome loss

For Human Artificial Chromosome (HAC) loss reporter assays, we used an HT-1080 cell line engineered with a HAC carrying an EGFP transgene and blasticidin selection marker (Lee et al, 2013). The HAC contains a functional centromere, therefore allowing stable inheritance as a nonessential chromosome; cells grown under selection (6μg/ml blasticidin) retain the HAC and EGFP fluorescence whereas upon selection removal, cells experience HAC loss at a rate x10 that of the native chromosomes, which is measured through loss of EGFP fluorescence by routine flow cytometry.

For screens, we established a 96-well high-throughput flow cytometry protocol to investigate the effect of each of our query genes on chromosome loss. Cells were removed from selection and transfected with a pool of four siRNAs/per gene (Dharmacon) with Lullaby (OZ Biosciences) as follows: OTNT: non-targeting, serving as negative control, the essential mitotic gene OIP5 and tumour suppressor TP53 as positive controls, alongside our 37 gene library, and UBB as a siRNA transfection efficiency control. Cells were grown for 72 hours either in the absence of treatment or exposure to low-level hydroxyurea (HU; 1mM) for the last 16h and thoroughly washed (x5) in PBS to remove the drug. Subsequently, cells were maintained in culture, in the absence of selection, for an additional 14 days, through split/re-seed cycles at a 1/5 split ratio every 4 days (time to confluence). At endpoint, cells were harvested by trypsinisation and single-cell suspensions of live cells were processed for EGFP expression on a MACSQuant Analyser (Miltenyi Biotec).

Experiments were run in triplicate wells, across three independent biological repeats. The raw scores (percentage of cells that lost the HAC by proxy of EGFP loss) were used to calculate fold changes (LFC) and Strictly standardized mean difference (SSMD) scores. LFCs were calculated for each replicate, where each well was divided by the mean NT control wells on the plate. SSMD was calculated for each gene (positive control) against the NT control (negative control) using the method:

$$\hat{\beta} = \frac{\tilde{X}_P - \tilde{X}_N}{1.4826\sqrt{\tilde{s}_P{}^2 + \tilde{s}_N{}^2}}$$

Where $\tilde{X}_P$, $\tilde{X}_N$, $\tilde{s}_P$, and $\tilde{s}_N$ are the medians and median absolute deviations in the positive and negative controls, respectively.

Drug sensitivity assays

For the clonogenic survival assays, 300 cells were seeded per well of a 6-well plate in duplicate. Drugs were added 18 hours after plating and cells were grown for 14 days before staining. Surviving colonies were stained with crystal violet and images were scanned and analysed using the GelCount colony counter (Oxford Optronix). 3 biological replicates were carried out for each condition.

To measure growth rate trajectories, HCT116-iRFP cells (Hock et al., 2014) were used. Cells were transfected with the relevant siRNA as described above. After 48 hours, cells were trypsinized and counted using a Vi-Cell XR cell counter (Beckman Coulter). 1000 cells were seeded per well in 96 well plates. A baseline iRFP reading was recorded 6 hours post adhesion by scanning at 700 nM spectra using a Li-COR Odyssey CLx scanner (Li-COR). 500 nM Olaparib was added thereafter. iRFP readings were monitored daily from day 3-7 days after the initial baseline reading.

To determine Osimertinib dose responses in PC9 cells, 10000 cells were seeded per well in a 96 plate and left to adhere for 16 hours. Varying concentrations of the EGFR inhibitor Osimertinib (AZD9291, Selleckchem) were added for 7 days. Cell survival was determined using AlamarBlue cell viability Reagents (Invitrogen) scanned at 570 nm and 600 nm absorbance with a Tecan Infinite 2000 plate reader (Tecan Inc.), according to the manufacturer's instructions.

Clonal FISH

Following siRNA transfection, 100ul of cells were plated at 5000 cells/ml onto polylysine-coated glass slides within a 10 cm^2 tissue-culture dish and left for 4 hours to adhere. Dishes were subsequently filled with media and incubated for for 3-14 days until colonies reached approximately the 64-cell stage. Media was gently removed, and cells were washed 1x with PBS and treated with hypotonic solution (0.4% KCl-0.4% sodium citrate prewarmed to 37°C) for 7 min. Cells were fixed by incubating with fixation buffer (3:1 Methanol: Acetic Acid, prechilled to -20°C) for 30 min at room temperature and slides were air-dried for at least 1 week at RT. For hybridisation, slides were treated with SPOT-light tissue pre-treatment solution (Invitrogen) at 98˚C for 10 min, washed twice in PBS for 3 minutes, dehydrated through an ethanol series (70%-85%-100%), 5 min each and air-dried. Slides were then incubated with 10 μl probe mix (8µl hybridization buffer and 1 μl of each Vysis Centromere Enumeration Probe (CEP2 probe (06J36-027, orange spectrum and CEP15 probe (06J37-015, green spectrum Abbott), covered with a 22 x 22 mm coverslip and sealed with rubber cement. Slides were placed on a heating block at 95°C for 5 min for denaturation and incubated in a humidified chamber overnight at 37˚C. After removing the coverslip by washing with 0.5x SSC, slides were washed once with 0.5x SSC at 75˚C for 5 minutes and once with 0.5x SSC at RT for 5 minutes. Slides were rinsed with water and incubated with 1µg/ml DAPI for 15 min at RT. Slides were dehydrated through an ethanol series (70%-85%-100%) for 5 min each, air-dried, mounted in Vectashield and sealed with clear nail varnish. For analysis, slides were scanned using an Olympus VS120 slide scanner at 4X magnification, and discrete colonies were selected. Acquisition of colonies was performed at 20X magnification with 5 z-stacks of 1.3 µm. Individual cells were categorised according to their copy number state for the two chromosomes. The percentage of cells with a centromere copy number deviating from the mode for each colony was quantified.

100KGP WGS lung cohort

We used the whole genome sequencing data of lung tumours from the Genomics England Limited (GEL) version 8 cohort of the 100,000 Genomes Project. The GEL version 8 dataset can be accessed via https://www.genomicsengland.co.uk/about-gecip/for-gecip-members/data-and-data-access. After multiple steps of quality control (QC), 1011 lung tumours were included in this study. We excluded formalin-fixed paraffin embedded samples (FFPE) and low purity samples that failed copy number calling. Some samples were duplicated with discordant information in the cancer summary table provided by GEL which is why they were excluded from the final cohort.

100KGP WGS lung cohort Clinical data

Cancer histology in terms of disease subtype was curated by Genomics England and is as described in the 'cancer_analysis' table available to researchers within the Genomics England research environment. Disease subtype was set to "ADENOCARCINOMA" for samples with annotations "SOLID_CARCINOMA" and "MIXED_TUMOUR_TYPE" after assessing pathology reports of these tumours which indicated that these tumours are of adenocarcinoma histology with solid or mixed growth patterns.

100KGP WGS lung cohort sequencing and alignment

Tumour samples and matched germline blood samples were subject to whole genome sequencing at a median depth of 97x for tumour samples and 33x for germline samples.

100KGP WGS lung cohort correction for reference bias

The Illumina Isaac pipeline (Raczy et al., 2013) has been used in the 100,000 Genomes Project to align and process the whole genome sequencing data to the hg38 assembly. However, recent studies have demonstrated that the soft clipping of semi-aligned reads performed by the Isaac aligner leads to reference bias which affects the calling of somatic copy number alterations (SCNAs) as well as purity and cancer cell fraction (CCF) estimations in cancer (Cornish et. al, 2020). To address this caveat, a tool called fixVAF was developed by Cornish et al. (https://github.com/danchubb/FixVAF) to remove sources of reference bias ensuring a robust CCF estimation. We applied fixVAF to the BAM files and VCF files of the GEL lung cohort which were produced by the Genomics England core pipeline.

100KGP WGS lung cohort SNV and indel calling

As part of the Genomics England core pipeline, Strelka (Saunders et. al., 2012) has been applied for somatic variant calling. The resulting VCF files were corrected for biases in the variant allele frequency (VAF) by applying fixVAF which includes multiple filtering and QC steps. Additional filters for single nucleotide variants (SNVs) and INDELs, informed by the TRACERx pipeline (Frankell et. al., 2023), were applied. This includes that any variant located within a blacklist region of the genome, as used in the TRACERx pipeline and informed by the "blacklisted" regions reported on ENCODE (Amemiya et. al., 2019), were removed.

Additional filters that a SNV had to pass to be included in the final mutation table:

- VAF ≥ 5%
- alternative reads ≥ 5
- Germline VAF < 1%
- Germline number of alternative reads < 5
- Total depth ≥ 30

Additional filters that an INDEL had to pass to be included in the final mutation table:

- VAF ≥ 5%
- alternative reads ≥ 10
- Germline VAF < 1%
- Germline number of alternative reads < 5
- Total depth ≥ 50

100KGP WGS lung cohort Signature Extraction: SBS, ID

We extracted mutational signatures de novo using hierarchical Dirichlet processes (HDP) ("Patterns of Somatic Genome Rearrangement in Human Cancer," n.d.) implemented in the R package hdp (v.0.1.5) available on GitHub (https://github.com/nicolaroberts/hdp). Variants were classified into mutational channels in accordance to COSMIC,resulting in mutation type specific counts matrices which were used as input to HDP. We defined a tree of parent Dirichlet process (DP) nodes to define hierarchies of relatedness between samples. The HDP was structured to have one grandparent DP, two parent DPs representing the time point of sampling (primary and metastasis) and the individual tumours within each time point group as a child DP (primary=969 and met=42). This allowed for refined signature extraction of potentially treatment related signatures in the metastasis sub-cohort. Tumours were excluded for the according mutation type specific signature extraction if they had less than 50 SBSs or 50 IDs.

Signatures that were previously identified to be commonly active in lung cancer were included as priors. For SBS this was SBS1, SBS2, SBS4, SBS5, SBS13, SBS17b, SBS40, SBS92 for ID signatures ID2 and ID3. This means that for each prior, a cluster was initialised at the start of the algorithm and the prior signature mutation channel was provided as prior knowledge to force the algorithm to look for those signatures in the data. In addition, ten random clusters were initialised to detect de novo signatures that were not included in the list of priors. The model was initialised by applying the function hdp_init(). The mutation channel profiles were assigned to the leaves by hdp_setdata(), and the nodes were activated by dp_activate(). By applying hdp_posterior() 15 times with different seeds, 15 independent posterior sampling chains were constructed followed by 10,000 burn-in iterations and the collection of 100 posterior samples off each chain with 200 iterations between each. The hdp_multi_chain() function was applied to combine the results of the 15 chains, from which the final components were extracted using hdp_extract_components(). A mutational signature was deemed as present in a sample if more than or equal to 5% of mutations in that sample were associated with this signature. We compared the extracted mutational signatures to the reference signatures on COSMIC using cosine similarity. To assign a previously described signature to any of the de novo extracted signatures we used a cosine similarity threshold of 0.85 and declared the signature to be novel if the cosine similarity was smaller than 0.85 to any of the previously described signatures.

GISTIC2.0 peak identification

The copy number profiles for all regions of the same patient underwent uniform segmentation. For each segment, the highest and lowest log2 copy number values from all regions were chosen. GISTIC2.0 (Mermel et. al, 2011) was executed with the lowest log2 copy number values (to investigate losses). Somatic copy number losses were defined as ploidy-normalized copy number less than log2(1.5/2). Significance was defined as ($q<0.1$).

Reduced representation bisulfite sequencing and methylation status analysis

Methylation profiles from 217 tumor and 59 normal adjacent tissue samples from 59 patients were analyzed by reduced representation bisulphite sequencing (RRBS). Sequencing was performed using the NuGEN Ovation RRBS Methyl-Seq System that incorporates unique molecular barcodes (UMIs) facilitating single-molecule analysis and precise methylation estimates. Bisulfite conversion was performed using Qiagen's EpiTect Fast DNA Bisulphite Kit. Agencourt® and RNAClean® XP magnetic beads were used to purify converted libraries amplified by PCR. Purified libraries were quantified by Qubit dsDNA HS Assay (Invitrogen) and quality was evaluated using the Agilent Bioanalyzer High Sensitivity DNA Assay (Agilent Technologies).

Differentially methylated regions (DMR) were called by binning CpGs into neighbourhoods and identifying DMP hotspots within these clusters. CpGs which fell within 100 bp of one another were grouped together. For each bin, the number of consecutive DMPs with effect size 0.2 and $p < 0.01$ compared with the paired normal tissue were computed. The methylation status in gene promoter (defined as starting 2.5kb upstream and ending 250bp downstream of the transcription start site) was used to compute the methylation status per gene.

The correlation between the expression and promoter methylation status was assessed using a Pearson's test. The difference in the number of promoter DMRs between LUAD and LUSC tumors was determined by a Chi-squared test.

TAI/LST/HRDLOH and wGII analyses

For the analysis shown in Figure 3D-F and Figure 4B, analyses evaluating the relationship between FAT1 status and HRD in lung cancer were conducted using two cohorts, including the TCGA Lung and TRACERx Lung (TRACERx, n=740 tumour regions from 224 patients with lung adenocarcinoma, and n=461 tumour regions from 127 patients with lung squamous cell carcinoma). Using TRACERx data, patient tumours were stratified into two groups based on the presence or absence of FAT1 driver mutations. Per sampled tumour region, HRD estimates were calculated using the scarHRD R package (https://github.com/sztup/scarHRD), which estimates the levels of three HRD measures (LOH, TAI, and LST) using NGS data (Raczy et al, 2013). Results of the three metrics were compared between stratified

groups at the tumour-level (mean score per metric across all regions from the same tumour); linear mixed effects modelling was applied, adjusting for the number of tumours/regions sampled. Leveraging data from TCGA, the observed probability for loss, given by the percentage of the genome that is lost in that sample, was generated as an aberration state (loss or no loss) for each sample separately. A point biserial correlation between aberration state and TAI/LST/HRDLOH/wGII was then calculated across samples. This process was repeated 10,000 times and a p-value was obtained for *FAT1* by counting the percentage of permutations showing a greater correlation coefficient than that observed for *FAT1*.

Nuclear morphometric measurements

DAPI-stained images of nuclei were segmented using the following procedure: First, images were corrected for uneven illumination and variations in brightness in individual nuclei. This was performed by dividing the original DAPI image with a 2D-Gaussian-blurred version (sigma=200). Next we performed thresholding on each image using the Li method (Li and Tam, 1998) to create a binary mask of all nuclei. This binary mask was post-processed to remove holes within objects, refined with morphological closing and the image border was cleared of objects to remove nuclei partially out of the field of view. Subsequently, we performed a marker-controlled distance transform watershed to separate all nuclear instances into separate labels. Markers were calculated from the Euclidean distance transform image using the peak local max method. Morphological features (area, solidity and eccentricity) of all nuclei were then calculated. All image processing functions used were from the Python library scikit-image, except for the function used to calculate the distance transform image, for which we used distance_transform_edt from the Python library Scipy.

REFERENCES

Amemiya, H.M., Kundaje, A. & Boyle, A.P. The ENCODE Blacklist: Identification of Problematic Regions of the Genome. Sci Rep 9, 9354 (2019).
Cornish, A.J. *et al.* Reference bias in the Illumina Isaac aligner. *Bioinformatics* **36**, 4671-4672 (2020).
Frankell, A.M. *et al.* The evolution of lung cancer and impact of subclonal selection in TRACERx. *Nature* **616**, 525-533 (2023).
Hock, A.K. et al. iRFP is a sensitive marker for cell number and tumor growth in high-throughput systems. Cell Cycle 13, 220-226 (2014).
Lee, H.S. et al. A new assay for measuring chromosome instability (CIN) and identification of drugs that elevate CIN in cancer cells. BMC Cancer 13, 252 (2013).
Li, C.H. & Tam, P.K.S. An iterative algorithm for minimum cross entropy thresholding. Pattern Recognition Letters 19, 771-776 (1998).
Mermel, C.H. et al. GISTIC2.0 facilitates sensitive and confident localization of the targets of focal somatic copy-number alteration in human cancers. Genome Biol 12, R41 (2011).
Raczy, C. *et al.* Isaac: ultra-fast whole-genome secondary analysis on Illumina sequencing platforms. *Bioinformatics* **29**, 2041-2043 (2013).
Saunders, C.T. *et al.* Strelka: accurate somatic small-variant calling from sequenced tumor-normal sample pairs. *Bioinformatics* **28**, 1811-1817 (2012).

Full plasmids sequences

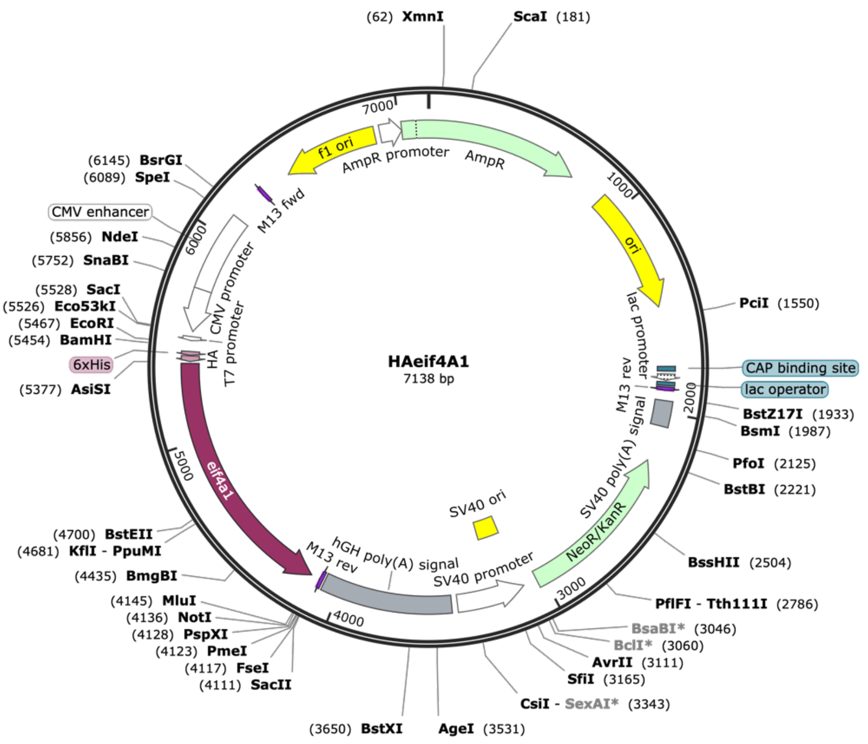

>HA-EIF4A1 7138bps

GGTTACATCGAACTGGATCTCAACAGCGGTAAGATCCTTGAGAGTTTTCGCCCCGAAGAACGTTTTCCAATGATGAGCACTTTTAA
AGTTCTGCTATGTGGCGCGGTATTATCCCGTATTGACGCCGGGCAAGAGCAACTCGGTCGCCGCATACACTATTCTCAGAATGACT
TGGTTGAGTACTCACCAGTCACAGAAAAGCATCTTACGGATGGCATGACAGTAAGAGAATTATGCAGTGCTGCCATAACCATGAGT
GATAACACTGCGGCCAACTTACTTCTGACAACGATCGGAGGACCGAAGGAGCTAACCGCTTTTTTGCACAACATGGGGGATCATGT
AACTCGCCTTGATCGTTGGGAACCGGAGCTGAATGAAGCCATACCAAACGACGAGCGTGACACCACGATGCCTGTAGCAATGGCAA
CAACGTTGCGCAAACTATTAACTGGCGAACTACTTACTCTAGCTTCCCGGCAACAATTAATAGACTGGATGGAGGCGGATAAAGTT
GCAGGACCACTTCTGCGCTCGGCCCTTCCGGCTGGCTGGTTTATTGCTGATAAATCTGGAGCCGGTGAGCGTGGGTCTCGCGGTAT
CATTGCAGCACTGGGGCCAGATGGTAAGCCCTCCCGTATCGTAGTTATCTACACGACGGGGAGTCAGGCAACTATGGATGAACGAA
ATAGACAGATCGCTGAGATAGGTGCCTCACTGATTAAGCATTGGTAACTGTCAGACCAAGTTTACTCATATATACTTTAGATTGAT
TTAAAACTTCATTTTTAATTTAAAAGGATCTAGGTGAAGATCCTTTTTGATAATCTCATGACCAAAATCCCTTAACGTGAGTTTTC
GTTCCACTGAGCGTCAGACCCCGTAGAAAAGATCAAAGGATCTTCTTGAGATCCTTTTTTTCTGCGCGTAATCTGCTGCTTGCAAA
CAAAAAAACCACCGCTACCAGCGGTGGTTTGTTTGCCGGATCAAGAGCTACCAACTCTTTTTCCGAAGGTAACTGGCTTCAGCAGA
GCGCAGATACCAAATACTGTTCTTCTAGTGTAGCCGTAGTTAGGCCACCACTTCAAGAACTCTGTAGCACCGCCTACATACCTCGC
TCTGCTAATCCTGTTACCAGTGGCTGCTGCCAGTGGCGATAAGTCGTGTCTTACCGGGTTGGACTCAAGACGATAGTTACCGGATA
AGGCGCAGCGGTCGGGCTGAACGGGGGGTTCGTGCACACAGCCCAGCTTGGAGCGAACGACCTACACCGAACTGAGATACCTACAG
CGTGAGCTATGAGAAAGCGCCACGCTTCCCGAAGGGAGAAAGGCGGACAGGTATCCGGTAAGCGGCAGGGTCGGAACAGGAGAGCG
CACGAGGGAGCTTCCAGGGGGAAACGCCTGGTATCTTTATAGTCCTGTCGGGTTTCGCCACCTCTGACTTGAGCGTCGATTTTTGT
GATGCTCGTCAGGGGGGCGGAGCCTATGGAAAAACGCCAGCAACGCGGCCTTTTTACGGTTCCTGGCCTTTTGCTGGCCTTTTGCT
CACATGTTCTTTCCTGCGTTATCCCCTGATTCTGTGGATAACCGTATTACCGCCTTTGAGTGAGCTGATACCGCTCGCCGCAGCCG
AACGACCGAGCGCAGCGAGTCAGTGAGCGAGGAAGCGGAAGAGCGCCCAATACGCAAACCGCCTCTCCCCGCGCGTTGGCCGATTC
ATTAATGCAGCTGGCACGACAGGTTTCCCGACTGGAAAGCGGGCAGTGAGCGCAACGCAATTAATGTGAGTTAGCTCACTCATTAG
GCACCCCAGGCTTTACACTTTATGCTTCCGGCTCGTATGTTGTGTGGAATTGTGAGCGGATAACAATTTCACACAGGAAACAGCTA
TGACCATGATTACGCCAAGCTCTAGCTAGAGGTCGACGGTATACAGACATGATAAGATACATTGATGAGTTTGGACAAACCACAAC
TAGAATGCAGTGAAAAAAATGCTTTATTTGTGAAATTTGTGATGCTATTGCTTTATTTGTAACCATTATAAGCTGCAATAAACAAG
TTGGGGTGGGCGAAGAACTCCAGCATGAGATCCCCGCGCTGGAGGATCATCCAGCCGGCGTCCCGGAAAACGATTCCGAAGCCCAA
CCTTTCATAGAAGGCGGCGGTGGAATCGAAATCTCGTGATGGCAGGTTGGGCGTCGCTTGGTCGGTCATTTCGAACCCCAGAGTCC
CGCTCAGAAGAACTCGTCAAGAAGGCGATAGAAGGCGATGCGCTGCGAATCGGGAGCGGCGATACCGTAAAGCACGAGGAAGCGGT
CAGCCCATTCGCCGCCAAGCTCTTCAGCAATATCACGGGTAGCCAACGCTATGTCCTGATAGCGGTCGGCCACACCCAGCCGGCCA
CAGTCGATGAATCCAGAAAAGCGGCCATTTTCCACCATGATATTCGGCAAGCAGGCATCGCCATGGGTCACGACGAGATCCTCGCC
GTCGGGCATGCGCGCCTTGAGCCTGGCGAACAGTTCGGCTGGCGCGAGCCCCTGATGCTCTTCGTCCAGATCATCCTGATCGACAA
GACCGGCTTCCATCCGAGTACGTGCTCGCTCGATGCGATGTTTCGCTTGGTGGTCGAATGGGCAGGTAGCCGGATCAAGCGTATGC
AGCCGCCGCATTGCATCAGCCATGATGGATACTTTCTCGGCAGGAGCAAGGTGAGATGACAGGAGATCCTGCCCCGGCACTTCGCC
CAATAGCAGCCAGTCCCTTCCCGCTTCAGTGACAACGTCGAGCACAGCTGCGCAAGGAACGCCCGTCGTGGCCAGCCACGATAGCC
GCGCTGCCTCGTCCTGCAGTTCATTCAGGGCACCGGACAGGTCGGTCTTGACAAAAAGAACCGGGCGCCCCTGCGCTGACAGCCGG
AACACGGCGGCATCAGAGCAGCCGATTGTCTGTTGTGCCCAGTCATAGCCGAATAGCCTCTCCACCCAAGCGGCCGGAGAACCTGC
GTGCAATCCATCTTGTTCAATCATGCGAAACGATCCTCATCCTGTCTCTTGATCAGATCCGAAAATGGATATACAAGCTCCCGGGA
GCTTTTTGCAAAAGCCTAGGCCTCCAAAAAAGCCTCCTCACTACTTCTGGAATAGCTCAGAGGCCGAGGCGGCCTCGGCCTCTGCA
TAAATAAAAAAAATTAGTCAGCCATGGGGCGGAGAATGGGCGGAACTGGGCGGAGTTAGGGGCGGGATGGGCGGAGTTAGGGGCGG
GACTATGGTTGCTGACTAATTGAGATGCATGCTTTGCATACTTCTGCCTGCTGGGGAGCCTGGGGACTTTCCACACCTGGTTGCTG
ACTAATTGAGATGCATGCTTTGCATACTTCTGCCTGCTGGGGAGCCTGGGGACTTTCCACACCCTAACTGACACACATTCCACAGC

CAAGCTGGCCGCGTACCCAATTCAACAGGCATCTACTGAGTGGACCCAACGCATGAGAGGACAGTGCCAAGCAAGCAACTCAAATG
TCCCACCGGTTGGGCATGGCCAGGTAGCCTATGCTGTGTCTGGACGTCCTCCTGCTGGTATAGTTATTTTAAAATCAGAAGGACAG
GGAAGGGAGCAGTGGTTCACGCCTGTAATCCCAGCAATTTGGGAGGCCAAGGTGGGTAGATCACCTGAGATTAGGAGTTGGAGACC
AGCCTGGCCAATATGGTGAAACCCCGTCTCTACCAAAAAAACAAAAATTAGCTGAGCCTGGTCATGCATGCCTGGAATCCCAACAA
CTCGGGAGGCTGAGGCAGGAGAATCGCTTGAACCCAGGAGGCGGAGATTGCAGTGAGCCAAGATTGTGCCACTGCACTCCAGCTTG
GTTCCCAATAGACCCCGCAGGCCCTACAGGTTGTCTTCCCAACTTGCCCCTTGCTCCATACCACCCCCCTCCACCCCATAATATTA
TAGAAGGACACCTAGTCAGACAAAATGATGCAACTTAATTTTATTAGGACAAGGCTGGTGGGCACTGGAGTGGCAACTTCCAGGGC
CAGGAGAGGCACTGGGGAGGGGTCACAGGGATGCCACCCGGGATCTGTTCAGGAAACAGCTATGACCGCGGCCGGCCGTTTAAACC
TCGAGTGCGGCCGCTCACGCGTTCAGATGAGGTCAGCAACATTGAGGGGCATTTCCTCAATGGAGGTGTTGTAGAAGGTCTCAATG
TCTCGAAGAGTCCTCTTGTCTTCTTCTGTCACCATGTTAATAGCCACACCTTTACGGCCAAACCGTCCACCTCGACCGATTCTGTG
GATATAGTTTTCCCTGTTGGTGGGAAGGTCATAGTTGATGACTAAAGAAACCTGCTGCACATCAATGCCTCTGGCCAGCAGGTCAG
TGGTAATCAAAACTCTGCTAGAGCCAGAACGAAACTCCCTCATAATCACGTCTCGTTCCTTTTGGTCCATATCTCCATGCATGGCG
GATACAGTGAAATCTCGAGCATGCATCTTCTCGGTGAGCCAGTCCACCTTCCTCCGGGTGTTGATGAAGATGACTGCCTGGGTGAT
GGTCAGGGTTTCATACAAGTCACATAGTGTGTCCAGCTTCCACTCCTCTCGTTCCACGTTGATGTAGAACTGGCGGATACCCTCCA
GGGTCAACTCTTCCTTCTTGACAAGAATCCGAATGGGGTCCCTCATGAACTTCTTGGTCACCTCAAGCACATCAGAAGGCATTGTG
GCTGACAGCAAAACTACCTGGGTGTTGCTGTTGAGCTTTTGGAATATGTCATAGATCTGGTCCTTGAATCCACGGCTTAACATTTC
GTCAGCTTCATCCAGTACAAACATCTTGATGTATTTGGGGGACAGGTATCTCCGGTTAAGCATATCAAACACACGGCCAGGGGTAC
CCACGATGATGTGGGGAGCTTCCATCTGCAGTTTCTGCACCTCAGCACGCACGTTGGTGCCCCCGATACAGGCGTGACAGGAGGCG
CCCATGTAGTCTCCTAGTGCCATGACCACCTTCTGTATCTGCTGAGCCAATTCTCGAGTGGGTGCTAGGACCAAGGCCTGGGTGGC
TTTTAGATCTAATTCAATCTGCTGCAGAATTGATATGGCAAATGTGGCCGTTTTCCCAGTCCCAGATTGGGCTTGAGCAATCACAT
CATAACCCTTGATACAAGGTAGAATGGCTCGCTGCTGGATGGCAGAGGGCTTCTCAAAACCATAGGCGTAGATGCCACGGAGAAGG
GACTCCGAGAGGTTCATGTCATCAAAGCTGTCAACAATCTCATTCCAGTTACTCTCGATGACGCCTTCGGGCTCCATCCCATCGGG
GCCATTGTCTCTGGATCGGGAATCCTGGCTCGCAGACATGGCGATCGCAGCGTAATCTGGAACATCGTATGGGTAATCGTGATGGT
GATGGTGATGCATGGTGGCAGATCTCCTCGGTACCGGATCCAGTCGACGAATTCCCGGCCGCCCTATAGTGAGTCGTATTACAAAA
TTCTGACGGTTCACTAAACGAGCTCTGCTTATATAGACCTCCCACCGTACACGCCTACCGCCCATTTGCGTCAACGGGGCGGGGTT
ATTACGACATTTTGGAAAGTCCCGTTGATTTTGGTGCCAAAACAAACTCCCATTGACGTCAATGGGGTGGAGACTTGGAAATCCCC
GTGAGTCAAACCGCTATCCACGCCCATTGGTGTACTGCCAAAACCGCATCACCATGGTAATAGCGATGACTAATACGTAGATGTAC
TGCCAAGTAGGAAAGTCCCGTAAGGTCATGTACTGGGCATAATGCCAGGCGGGCCATTTACCGTCATTGACGTCAATAGGGGGCGG
ACTTGGCATATGATACACTTGATGTACTGCCAAGTGGGCAGTTTACCGTAAATACTCCACCCATTGACGTCAATGGAAAGTCCCTA
TTGGCGTTACTATGGGAACATACGTCATTATTGACGTCAATGGGCGGGGGTCGTTGGGCGGTCAGCCAGGCGGGCCATTTACCGTA
AGTTATGTAACGCGGAACTCCATATATGGGCTATGAACTAATGACCCCGTAATTGATTACTATTAATAACTAGTCAATAATCAATG
TCAACATGGCGGTCATATTGGACATGAGCCAATATAAATGTACATATTATGATATAGATACAACGTATGCAATGGCCAATAGCCAA
TATTGATTTATGCTATATAACCAATGACTAATATGGCTAATTGCCAATATTGATTCAATGTATAGATCAGCTTGGCACTGGCCGTC
GTTTTACAACGTCGTGACTGGGAAAACCCTGGCGTTACCCAACTTAATCGCCTTGCAGCACATCCCCCTTTCGCCAGCTGGCGTAA
TAGCGAAGAGGCCCGCACCGATCGCCCTTCCCAACAGTTGCGCAGCCTGAATGGCGAATGGAAATTGTAAGCGTTAATATTTTGTT
AAAATTCGCGTTAAATTTTTGTTAAATCAGCTCATTTTTTAACCAATAGGCCGAAATCGGCAAAATCCCTTATAAATCAAAAGAAT
AGACCGAGATAGGGTTGAGTGTTGTTCCAGTTTGGAACAAGAGTCCACTATTAAAGAACGTGGACTCCAACGTCAAAGGGCGAAAA
ACCGTCTATCAGGGCGATGGCCCACTACGTGAACCATCACCCTAATCAAGTTTTTTGGGGTCGAGGTGCCGTAAAGCACTAAATCG
GAACCCTAAAGGGAGCCCCCGATTTAGAGCTTGACGGGGAAAGCCGGCGAACGTGGCGAGAAAGGAAGGGAAGAAAGCGAAAGGAG
CGGGCGCTAGGGCGCTGGCAAGTGTAGCGGTCACGCTGCGCGTAACCACCACACCCGCCGCGCTTAATGCGCCGCTACAGGGCGCG
TCAGGTGGCACTTTTCGGGGAAATGTGCGCGGAACCCCTATTTGTTTATTTTTCTAAATACATTCAAATATGTATCCGCTCATGAG
ACAATAACCCTGATAAATGCTTCAATAATATTGAAAAAGGAAGAGTATGAGTATTCAACATTTCCGTGTCGCCCTTATTCCCTTTT
TTGCGGCATTTTGCCTTCCTGTTTTTGCTCACCCAGAAACGCTGGTGAAAGTAAAAGATGCTGAAGATCAGTTGGGTGCACGAGTG

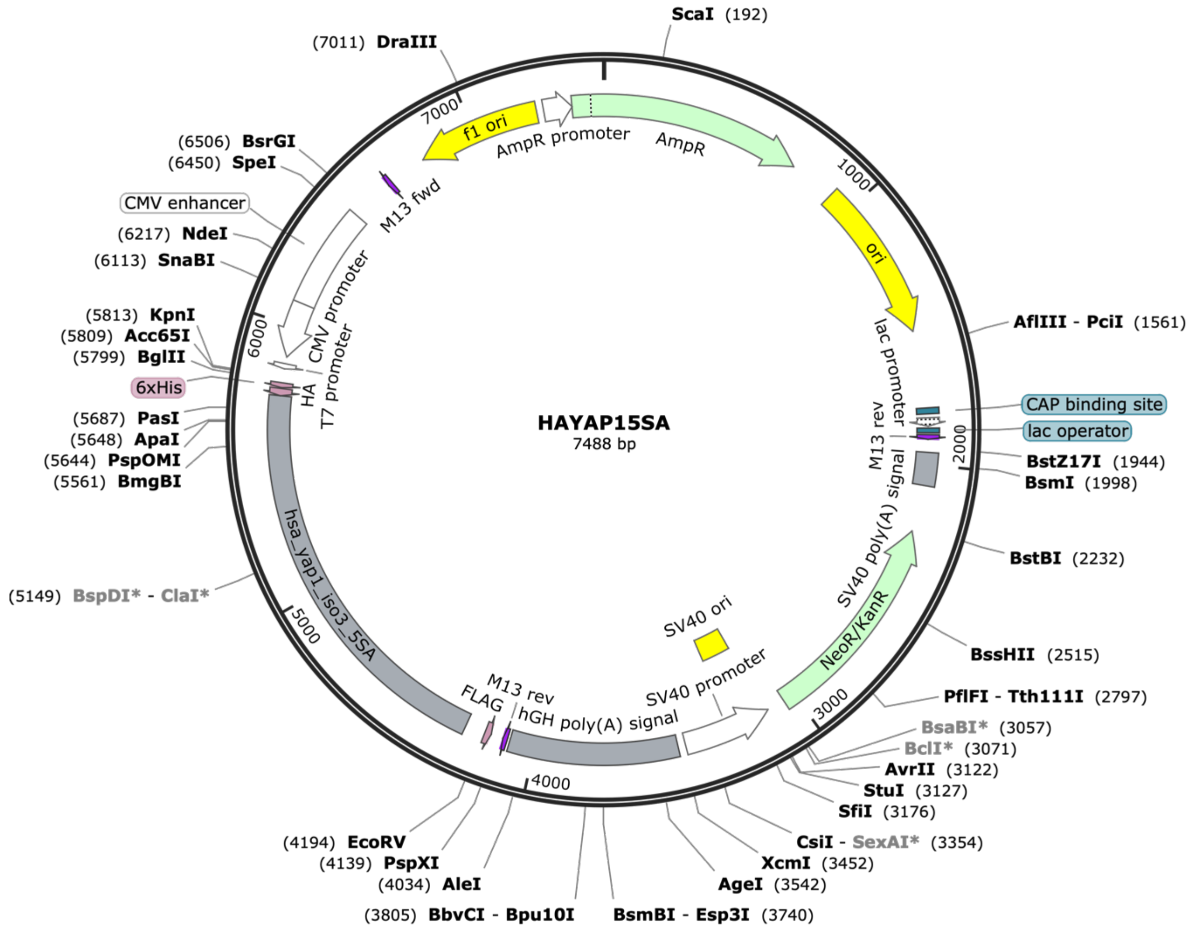

> **HA-YAP1^{5SA} 7488bps**

GTGCACGAGTGGGTTACATCGAACTGGATCTCAACAGCGGTAAGATCCTTGAGAGTTTTCGCCCCGAAGAACGTTTTCCAATGATG
AGCACTTTTAAAGTTCTGCTATGTGGCGCGGTATTATCCCGTATTGACGCCGGGCAAGAGCAACTCGGTCGCCGCATACACTATTC
TCAGAATGACTTGGTTGAGTACTCACCAGTCACAGAAAAGCATCTTACGGATGGCATGACAGTAAGAGAATTATGCAGTGCTGCCA
TAACCATGAGTGATAACACTGCGGCCAACTTACTTCTGACAACGATCGGAGGACCGAAGGAGCTAACCGCTTTTTTGCACAACATG
GGGGATCATGTAACTCGCCTTGATCGTTGGGAACCGGAGCTGAATGAAGCCATACCAAACGACGAGCGTGACACCACGATGCCTGT
AGCAATGGCAACAACGTTGCGCAAACTATTAACTGGCGAACTACTTACTCTAGCTTCCCGGCAACAATTAATAGACTGGATGGAGG
CGGATAAAGTTGCAGGACCACTTCTGCGCTCGGCCCTTCCGGCTGGCTGGTTTATTGCTGATAAATCTGGAGCCGGTGAGCGTGGG
TCTCGCGGTATCATTGCAGCACTGGGGCCAGATGGTAAGCCCTCCCGTATCGTAGTTATCTACACGACGGGGAGTCAGGCAACTAT
GGATGAACGAAATAGACAGATCGCTGAGATAGGTGCCTCACTGATTAAGCATTGGTAACTGTCAGACCAAGTTTACTCATATATAC
TTTAGATTGATTTAAAACTTCATTTTTAATTTAAAAGGATCTAGGTGAAGATCCTTTTTGATAATCTCATGACCAAAATCCCTTAA
CGTGAGTTTTCGTTCCACTGAGCGTCAGACCCCGTAGAAAAGATCAAAGGATCTTCTTGAGATCCTTTTTTTCTGCGCGTAATCTG
CTGCTTGCAAACAAAAAAACCACCGCTACCAGCGGTGGTTTGTTTGCCGGATCAAGAGCTACCAACTCTTTTTCCGAAGGTAACTG
GCTTCAGCAGAGCGCAGATACCAAATACTGTTCTTCTAGTGTAGCCGTAGTTAGGCCACCACTTCAAGAACTCTGTAGCACCGCCT
ACATACCTCGCTCTGCTAATCCTGTTACCAGTGGCTGCTGCCAGTGGCGATAAGTCGTGTCTTACCGGGTTGGACTCAAGACGATA
GTTACCGGATAAGGCGCAGCGGTCGGGCTGAACGGGGGGTTCGTGCACACAGCCCAGCTTGGAGCGAACGACCTACACCGAACTGA
GATACCTACAGCGTGAGCTATGAGAAAGCGCCACGCTTCCCGAAGGGAGAAAGGCGGACAGGTATCCGGTAAGCGGCAGGGTCGGA
ACAGGAGAGCGCACGAGGGAGCTTCCAGGGGGAAACGCCTGGTATCTTTATAGTCCTGTCGGGTTTCGCCACCTCTGACTTGAGCG
TCGATTTTTGTGATGCTCGTCAGGGGGGCGGAGCCTATGGAAAAACGCCAGCAACGCGGCCTTTTTACGGTTCCTGGCCTTTTGCT
GGCCTTTTGCTCACATGTTCTTTCCTGCGTTATCCCCTGATTCTGTGGATAACCGTATTACCGCCTTTGAGTGAGCTGATACCGCT
CGCCGCAGCCGAACGACCGAGCGCAGCGAGTCAGTGAGCGAGGAAGCGGAAGAGCGCCCAATACGCAAACCGCCTCTCCCCGCGCG
TTGGCCGATTCATTAATGCAGCTGGCACGACAGGTTTCCCGACTGGAAAGCGGGCAGTGAGCGCAACGCAATTAATGTGAGTTAGC
TCACTCATTAGGCACCCCAGGCTTTACACTTTATGCTTCCGGCTCGTATGTTGTGTGGAATTGTGAGCGGATAACAATTTCACACA
GGAAACAGCTATGACCATGATTACGCCAAGCTCTAGCTAGAGGTCGACGGTATACAGACATGATAAGATACATTGATGAGTTTGGA
CAAACCACAACTAGAATGCAGTGAAAAAAATGCTTTATTTGTGAAATTTGTGATGCTATTGCTTTATTTGTAACCATTATAAGCTG
CAATAAACAAGTTGGGGTGGGCGAAGAACTCCAGCATGAGATCCCCGCGCTGGAGGATCATCCAGCCGGCGTCCCGGAAAACGATT
CCGAAGCCCAACCTTTCATAGAAGGCGGCGGTGGAATCGAAATCTCGTGATGGCAGGTTGGGCGTCGCTTGGTCGGTCATTTCGAA
CCCCAGAGTCCCGCTCAGAAGAACTCGTCAAGAAGGCGATAGAAGGCGATGCGCTGCGAATCGGGAGCGGCGATACCGTAAAGCAC
GAGGAAGCGGTCAGCCCATTCGCCGCCAAGCTCTTCAGCAATATCACGGGTAGCCAACGCTATGTCCTGATAGCGGTCGGCCACAC
CCAGCCGGCCACAGTCGATGAATCCAGAAAAGCGGCCATTTTCCACCATGATATTCGGCAAGCAGGCATCGCCATGGGTCACGACG
AGATCCTCGCCGTCGGGCATGCGCGCCTTGAGCCTGGCGAACAGTTCGGCTGGCGCGAGCCCCTGATGCTCTTCGTCCAGATCATC
CTGATCGACAAGACCGGCTTCCATCCGAGTACGTGCTCGCTCGATGCGATGTTTCGCTTGGTGGTCGAATGGGCAGGTAGCCGGAT
CAAGCGTATGCAGCCGCCGCATTGCATCAGCCATGATGGATACTTTCTCGGCAGGAGCAAGGTGAGATGACAGGAGATCCTGCCCC
GGCACTTCGCCCAATAGCAGCCAGTCCCTTCCCGCTTCAGTGACAACGTCGAGCACAGCTGCGCAAGGAACGCCCGTCGTGGCCAG
CCACGATAGCCGCGCTGCCTCGTCCTGCAGTTCATTCAGGGCACCGGACAGGTCGGTCTTGACAAAAAGAACCGGGCGCCCCTGCG

CTGACAGCCGGAACACGGCGGCATCAGAGCAGCCGATTGTCTGTTGTGCCCAGTCATAGCCGAATAGCCTCTCCACCCAAGCGGCC
GGAGAACCTGCGTGCAATCCATCTTGTTCAATCATGCGAAACGATCCTCATCCTGTCTCTTGATCAGATCCGAAAATGGATATACA
AGCTCCCGGGAGCTTTTTGCAAAAGCCTAGGCCTCCAAAAAAGCCTCCTCACTACTTCTGGAATAGCTCAGAGGCCGAGGCGGCCT
CGGCCTCTGCATAAATAAAAAAAATTAGTCAGCCATGGGGCGGAGAATGGGCGGAACTGGGCGGAGTTAGGGGCGGGATGGGCGGA
GTTAGGGGCGGGACTATGGTTGCTGACTAATTGAGATGCATGCTTTGCATACTTCTGCCTGCTGGGGAGCCTGGGGACTTTCCACA
CCTGGTTGCTGACTAATTGAGATGCATGCTTTGCATACTTCTGCCTGCTGGGGAGCCTGGGGACTTTCCACACCCTAACTGACACA
CATTCCACAGCCAAGCTGGCCGCGTACCCAATTCAACAGGCATCTACTGAGTGGACCCAACGCATGAGAGGACAGTGCCAAGCAAG
CAACTCAAATGTCCCACCGGTTGGGCATGGCCAGGTAGCCTATGCTGTGTCTGGACGTCCTCCTGCTGGTATAGTTATTTTAAAAT
CAGAAGGACAGGGAAGGGAGCAGTGGTTCACGCCTGTAATCCCAGCAATTTGGGAGGCCAAGGTGGGTAGATCACCTGAGATTAGG
AGTTGGAGACCAGCCTGGCCAATATGGTGAAACCCCGTCTCTACCAAAAAAACAAAAATTAGCTGAGCCTGGTCATGCATGCCTGG
AATCCCAACAACTCGGGAGGCTGAGGCAGGAGAATCGCTTGAACCCAGGAGGCGGAGATTGCAGTGAGCCAAGATTGTGCCACTGC
ACTCCAGCTTGGTTCCCAATAGACCCCGCAGGCCCTACAGGTTGTCTTCCCAACTTGCCCCTTGCTCCATACCACCCCCCTCCACC
CCATAATATTATAGAAGGACACCTAGTCAGACAAAATGATGCAACTTAATTTTATTAGGACAAGGCTGGTGGGCACTGGAGTGGCA
ACTTCCAGGGCCAGGAGAGGCACTGGGGAGGGGTCACAGGGATGCCACCCGGGATCTGTTCAGGAAACAGCTATGACCGCGGCCGG
CCGTTTAAACCTCGAGTGCCGGCCGGCCGTTTAAACCTTATCGTCGTCATCCTTGTAATCCAGGATATCATTTGCTGCCAGATCCT
CTTCTGAGATGAGTTTCTGATCTGCAGAATTCAGTCTGCCTGAGGGCTCTATAACCATGTAAGAAAGCTTTCTTTATCTAGCTTGG
TGGCAGCCAAAACAGACTCCATGTCATTAAGGATGTCAGAACTCAAAGCTTCCTGCAGACTTGGCATCAGCTCCTCTCCTTCTATG
TTCATTCCATCTCCTTCCAGTGTTCCAAGGTCCACATTTGTCCCAGGAATGGCTTCAAGGTAGTCTGGGAAACGGTTCTGCTGTGA
GGGCAGGGTGCTTTGGTTGATAGTATCACCTGTATCCATCTCATCCACACTGTTCAGGAAGTCATCTGGGGTTCGAGGGACACTGT
AGCTGCTCATGCTTAGTCCACTGTCTGTAGCCTCATCTCGAGAGTGATAGGTGCCACTGTTAAGGAAAGGATCTGAGCTATTGGTC
GTCATTGTTCTCAATTCCTGAGACATCCCGGGAGAAGACACTGGATTTTGAGTCCCACCATCCTGCTCCAGTGTTGGTAACTGGCT
ACGCAGGGCTAACTCCTGCCGAAGCAGTTCTTGCTGTTTCAGCCGCAGCCTCTCCTTCTCCATCTGCAGTTGCTGCAGTCGCATCT
GTTGCTGCTGGTTGGAGTTGCTGCCACCCATGACGCCTCCCTGTGGGCTCTGGGGAGCCAGGGGTGGTGGCTGTTTCACTGGAGCA
CTCTGACTGATTCTCTGGTTCATGGCAAAACGAGGGTCAAGCCTTGGGTCTAGCCAAGAGGTGGTCTTGTTCTTATGGTTTATATA
GTAAATTTCTCCATCCTGAGTCATGGCTTGTTCCCATCCATCAGGAAGAGGACCTGAAGCCGAGTTCATCATATTCTGCTGCACTG
GTGGACTGGTGGGGGCTGTGACGTTCATCTGGGACAGCATGGCCTTCCTGGGGTCCTGCCATGTTGTTGTCTGATCGATGTGATTT
AAGAAGTATCTCTGACCAGAAGATGTCTTTGCCATCTCCCAACCTGCTGGCAGAGGTACATCATCAGGTATCTCAAAAGCAGCCTG
TCGAAGATGCTGAGCTGTGGGTGTAGCTGCTGGGCCAGAGACTACTCCAGTGGGGGTCAGTGTCCCAGGAGAAACAGCTCCCAACT
GCAGAGCAGCTGGAGCGGCATGAGCTCGAACATGCTGTGGAGTCAGGGCTCCTGCAGTGCCTGCATCAGTAGCGGCCTGTCGGGAG
TGGGATTTGGGCTCCGGCGGCTTGAAGAAGGAGTCGGGCAGCTTCCGGAGCCTCATGGGCACGGTCTGGGGCACGTTGGCCGTCTT
GGGGTTCATGACGGCGTTGAAGAGCGCCTCCAGGTCGGTCTCCGCGTCCCCGCGGACGTGCACGATCTGATGCCCGGCGGGGGGTG
CCTGCGGCGCCGCCTGGGTCGCCGCGGGTGCCGGTTGCCCGGGTCCGGACGGCGGGCCCTGCCCCTGCGGGGGCTGCGAAGGCGGC
TGCCCTTGGCCCTGGGGGGCCGGTTGAGGCGGCGGCTGCTGCCCGGGATCCTTAGGTACTATCGCAGCGTAATCTGGAACATCGTA
TGGGTAATCGTGATGGTGATGGTGATGCATGGTGGCAGATCTCCTCGGTACCGGATCCAGTCGACGAATTCCCGGCCGCCCTATAG
TGAGTCGTATTACAAAATTCTGACGGTTCACTAAACGAGCTCTGCTTATATAGACCTCCCACCGTACACGCCTACCGCCCATTTGC
GTCAACGGGGCGGGGTTATTACGACATTTTGGAAAGTCCCGTTGATTTTGGTGCCAAAACAAACTCCCATTGACGTCAATGGGGTG
GAGACTTGGAAATCCCCGTGAGTCAAACCGCTATCCACGCCCATTGGTGTACTGCCAAAACCGCATCACCATGGTAATAGCGATGA
CTAATACGTAGATGTACTGCCAAGTAGGAAAGTCCCGTAAGGTCATGTACTGGGCATAATGCCAGGCGGGCCATTTACCGTCATTG
ACGTCAATAGGGGGCGGACTTGGCATATGATACACTTGATGTACTGCCAAGTGGGCAGTTTACCGTAAATACTCCACCCATTGACG
TCAATGGAAAGTCCCTATTGGCGTTACTATGGGAACATACGTCATTATTGACGTCAATGGGCGGGGGTCGTTGGGCGGTCAGCCAG
GCGGGCCATTTACCGTAAGTTATGTAACGCGGAACTCCATATATGGGCTATGAACTAATGACCCCGTAATTGATTACTATTAATAA
CTAGTCAATAATCAATGTCAACATGGCGGTCATATTGGACATGAGCCAATATAAATGTACATATTATGATATAGATACAACGTATG
CAATGGCCAATAGCCAATATTGATTTATGCTATATAACCAATGACTAATATGGCTAATTGCCAATATTGATTCAATGTATAGATCA
GCTTGGCACTGGCCGTCGTTTTACAACGTCGTGACTGGGAAAACCCTGGCGTTACCCAACTTAATCGCCTTGCAGCACATCCCCCT
TTCGCCAGCTGGCGTAATAGCGAAGAGGCCCGCACCGATCGCCCTTCCCAACAGTTGCGCAGCCTGAATGGCGAATGGAAATTGTA
AGCGTTAATATTTTGTTAAAATTCGCGTTAAATTTTTGTTAAATCAGCTCATTTTTTAACCAATAGGCCGAAATCGGCAAAATCCC
TTATAAATCAAAAGAATAGACCGAGATAGGGTTGAGTGTTGTTCCAGTTTGGAACAAGAGTCCACTATTAAAGAACGTGGACTCCA
ACGTCAAAGGGCGAAAAACCGTCTATCAGGGCGATGGCCCACTACGTGAACCATCACCCTAATCAAGTTTTTTGGGGTCGAGGTGC
CGTAAAGCACTAAATCGGAACCCTAAAGGGAGCCCCCGATTTAGAGCTTGACGGGGAAAGCCGGCGAACGTGGCGAGAAAGGAAGG
GAAGAAAGCGAAAGGAGCGGGCGCTAGGGCGCTGGCAAGTGTAGCGGTCACGCTGCGCGTAACCACCACACCCGCCGCGCTTAATG
CGCCGCTACAGGGCGCGTCAGGTGGCACTTTTCGGGGAAATGTGCGCGGAACCCCTATTTGTTTATTTTTCTAAATACATTCAAAT
ATGTATCCGCTCATGAGACAATAACCCTGATAAATGCTTCAATAATATTGAAAAAGGAAGAGTATGAGTATTCAACATTTCCGTGT
CGCCCTTATTCCCTTTTTTGCGGCATTTTGCCTTCCTGTTTTTGCTCACCCAGAAACGCTGGTGAAAGTAAAAGATGCTGAAGATC
AGTTGG

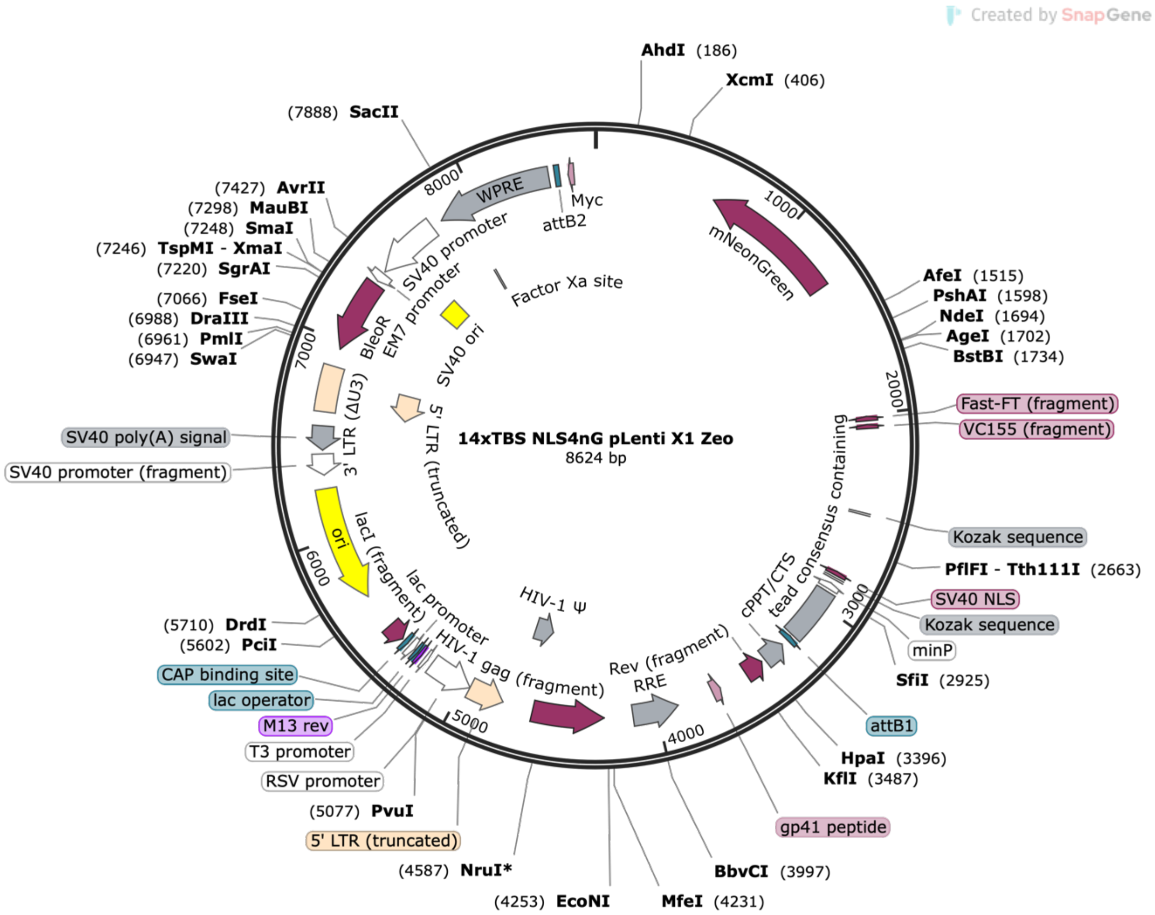

> 14XTEAD Binding Seqeuence 4xNeonGreen 8624bps

cgtacataggttgattttcagataatttgcggccataggcttagcaaaagtataggtggtccgggctgtgctgcgataccgtttt
ccgtttccagttgtgtaagaccacttgaatgtggaaatgattgttttgtcattcggatacgttttcttgcttctacaccaatctgc
agcagtcagactattagtcataacaggcccatctgcggggaagccagtgcctttgacttgggcttcgcccttgatatgtgaccctt
cgtaagtgtaccgatagttgaccgtcagactagcaccatcttcaaattgcatcgtccgatggacttggtatcctgatccatccacc
attgctgcctgaaatggactcattccgtccggatatggcaggtattgatgaaagccataaccaatatggggcacgagaatccaggg
tgaaaattgcaggtcccctttggtagatttcagattgagttcctcatatccgtcatttgggtttcccgtgccttggccgaccatat
cgaagtcaaccccgttaatacttccaaaaatatgcagctcgtgcgtagcggggagtgatgccatgttgtcctcttcgcccttggac
acgctgccaccacctttgtagagttcgtccatgcccataacatctgtgaaggccttttgccattctttaaagttgagttcggtctt
gctgtgtttcagctcagttttcctaaacacatacataggttgattcttgagatagttagctgccattggcttggcaaaggtatatg
ttgtcctggcggtagacctgtaccgtttgccattgcctgttgtatatgaccacttaaaagtagagataatggttttgtcatttggg
taagtcttttggagcggcaccagtctgctgcggtcagggaatttgtcatgactggcccatcggctggaaacccagtgcctttcac
ttgggcttcgcccttaatatgagatccttcataggtatagcggtagttgacggtgagagaggccccatcctcgaattgcatggttc
tatgcacttgatagccgcttccgtcgaccattgcggcttggaatggggacattccatccggatacggcagatattggtggaatcca
tatccaatgtgcggaaccaggatccatggggaaaattgcagatcgcccttcgtgcttttgagattcagttcctcataaccatcgtt
cggatttccagtgccttgtccaaccatatcgaaatcgacaccgttaatggagccgaaaatgtggagttcgtgagtagctggcagac
ttgccatattatcctcttcgcccttgctgacggatccgccgcctttgtagagttcatccattcccatgacatccgtaaaagccttt
tgccattctttgaaattcagctctgtcttagagtgcttgagttccgtctttctgaaaacgtacatcggttgattttgaggtaatt
ggcagccatgggcttagcgaatgtgtatgtggtccgagcggtggatctgtagcgctttccatttccggtcgtataagaccatttga
acgtagagatgatcgtcttatcattaggataggtctttttgctccgacaccagtcagcagctgtcaggctgtttgtcatcacaggt
ccgtcggcgggaaatccggtgcctttgacttgagcttcgcccttgatgtgagagccctcatatgtgtaccggtaattcactgtcag
ggaagcgccatcttcgaactgcattgtgcggtggacctgatagccagagccgtccaccatagcagcttgaaaggggctcatcccat
ctggatatgggagatactgatgaaacccgtatccgatatgtggcacgaggatccaaggagagaactggagatcccctttggtgctc
ttgaggttgagttcttcgtatccatcgttagggttccctgtgccttggccaaccatatcaaagtcgaccccattgatagagccaaa
gatatgcagttcatgagtggcgggcagagaagccatgttatcctcttcgcccttagagacagagcctccgcccttgtacagctcgt
ccatgcccatcacgtcggtgaaggctttctgccactctttgaagttcagttcggttttggagtgcttcagctcggtcttgcggaac
acgtacataggctggttcttcaggtagttggcggccataggtttggcgaaggtgtaggtggttctggcggtgcttctgtacctctt
gccgttgccggtggtgtaggaccacttgaaggtgctgatgatggtcttgtcgttggggtatgttttcttggacctgcaccaatcgg
cggctgtcagagaattggtcatcacggggccatcagcagggaagcctgtgcctttcacttgggcctcgcctttgatgtggctgccc
tcgtaggtgtatctgtagttcacggtcaggctggcgccgtcctcaaactgcatggtcctgtgcacctggtatccgctgccatcaac
catggcggcctggaaagggctcatgccgtcaggataaggcaggtactggtggaagccgtagccgatgtgaggcaccagaatccagg
ggctgaactgcaggtcgcccttggtggacttcaggttcagttcctcgtagccgtcattgggattgccggtgccttgtccgaccatg
tcgaagtccacgccgttgatgctgccgaagatgtgcagctcgtgtgtggcaggcaggctggccatattgtcctcttcgcctttga
cacgccggttccagagccattgccgccgactttgttgtagagtgtgggcatgacgtcgaactccaccttgcgcttcttcttgggg
ggctgcggtccatggtggcttcaacagtaccggattgccaagctggaagtcgagcttccattatataccctctaagcttggccgcc
gaggccagatcagtcattccttgcattccgctaggggcccgaggggtggaacagtcattccttgcattccgctagggcccgagggg
tggaacagtcattccttgcattccgctagatcttagcaaaatacgctgtgccagtcattccttgcattccgctagcctgggcccga

ggggtggaacagtcattccttgcattccgctaggggcccgaggggtggaacagtcattccttgcattccgctagggcccgaggggt
ggaacagtcattccttgcattccgctagggtaccgaagcctgctttttgtacaaacttgttgatatctgcagaattccaccacac
tggacgataaaattttgaattttgtaatttgtttttgtaattctttagtttgtatgtctgttgctattatgtctactattctttcc
cctgcactgtacccccaatccccccttttcttttaaaagttaaccgataccgtcgagatccgttcactaatcgaatggatctgtc
tctgtctctctctccaccttcttcttctattccttcgggcctgtcgggtcccctcggggttgggaggtgggtctgaaacgataatg
gtgaatatccctgcctaactctattcactatagaaagtacagcaaaaactattcttaaacctaccaagcctcctactatcattatg
aataattttatataccacagccaatttgttatgttaaaccaattccacaaacttgcccatttatctaattccaataattcttgttc
attcttttcttgctggttttgcgattcttcaattaaggagtgtattaagcttgtgtaattgttaatttctctgtcccactccatcc
aggtcgtgtgattccaaatctgttccagagatttattactccaactagcattccaaggcacagcagtggtgcaaatgagttttcca
gagcaaccccaaatccccaggagctgttgatcctttaggtatctttccacagccaggattcttgcctggagctgcttgatgcccca
gactgtgagttgcaacagatgctgttgcgcctcaatagccctcagcaaattgttctgctgctgcactataccagacaataattgtc
tggcctgtaccgtcagcgtcattgacgctgcgcccatagtgcttcctgctgctcccaagaacccaaggaacaaagctcctattccc
actgctctttttttctctctgcaccactcttctctttgccttggtgggtgctactcctaatggttcaatttttactactttatattt
atataattcacttctccaattgtccctcatatctcctcctccaggtctgaagatcagcggccgcttgctgtgcggtggtcttactt
ttgttttgctcttcctctatcttgtctaaagcttccttggtgtcttttatctctatcctttgatgcacacaatagagggttgctac
tgtattatataatgatctaagttcttctgatcctgtctgaagggatggttgtagctgtcccagtatttgtctacagccttctgatg
tttctaacaggccaggattaactgcgaatcgttctagctccctgcttgcccatactatatgttttaatttatatttttttctttccc
cctggccttaaccgaatttttcccatcgcgatctaattctcccccgcttaatactgacgctctcgcacccatctctctccttcta
gcctccgctagtcaaaatttttggcgtactcaccagtcgccgcccctcgcctcttgccgtgcgcgcttcagcaagccgagtcctgc
gtcgagagagctcctctggtttccctttcgctttcaagtccctgttcgggcgccactgctagagattttccacactgactaaaagg
gtctgagggatctctagttaccagagtcacacaacagacgggcacacactacttgaagcactcaaggcaagctttattgaggctta
agcagtgggttccctagttagccagagagctcccaggctcagatctggtctaaccagagagacccgtttatgtatcgagctaggca
cttaaatacaatatctctgcaatgcggcaattcagtggttcgtccaatccatgtcagacccgtctgttgccttcctaataaggcac
gatcgtaccaccttacttccaccaatcggcatgcacggtgctttttctctccttgtaaggcatgttgctaactcatcgttaccatg
ttgcaagactacaagagtattgcataagactacattaagcttgcagctccagcttttgttccctttagtgagggttaattgcgcgc
ttggcgtaatcatggtcatagctgtttcctgtgtgaaattgttatccgctcacaattccacacaacatacgagccggaagcataaa
gtgtaaagcctggggtgcctaatgagtgagctaactcacattaattgcgttgcgctcactgcccgctttccagtcgggaaacctgt
cgtgccagctgcattaatgaatcggccaacgcgcggggagaggcggtttgcgtattgggcgctcttccgcttcctcgctcactgac
tcgctgcgctcggtcgttcggctgcggcgagcggtatcagctcactcaaaggcggtaatacggttatccacagaatcaggggataa
cgcaggaaagaacatgtgagcaaaaggccagcaaaaggccaggaaccgtaaaaaggccgcgttgctggcgtttttccataggctcc
gcccccctgacgagcatcacaaaaatcgacgctcaagtcagaggtggcgaaacccgacaggactataaagataccaggcgtttccc
cctggaagctccctcgtgcgctctcctgttccgaccctgccgcttaccggatacctgtccgcctttctcccttcgggaagcgtggc
gctttctcatagctcacgctgtaggtatctcagttcggtgtaggtcgttcgctccaagctgggctgtgtgcacgaaccccccgttc
agcccgaccgctgcgccttatccggtaactatcgtcttgagtccaacccggtaagacacgacttatcgccactggcagcagccact
ggtaacaggattagcagagcgaggtatgtaggcggtgctacagagttcttgaagtggtggcctaactacggctacactagaagaac
agtatttggtatctgcgctctgctgaagccagttaccttcggaaaaagagttggtagctcttgatccggcaaacaaaccaccgctg
gtagcggtggtttttttgtttgcaagcagcagattacgcgcagaaaaaaaggatctcaagaagatcctttgatcttttctacgggg
tctgacgctcagtggaacgaaaactcacgttaagggattttggcggcctcggcctctgcataaataaaaaaaattagtcagccatg
gggcggagaatgggcggaactgggcggagttaggggcgggatgggcggagttaggggcgggatagctagagccagacatgataaga
tacattgatgagtttggacaaaccacaactagaatgcagtgaaaaaaatgctttatttgtgaaatttgtgatgctattgctttatt
tgtaaccattataagctgcaataaacaagttcctctcactctctgatattcatttctttgcaagttataaatactgaataataaga
tgacatgaactactactgctagagattttccacactgactaaaagggtctgagggatctctagttaccagagtcacacaacagacg
ggcacacactacttgaagcactcaaggcaagctttattgaggcttaagcagtgggttccctagttagccagagagctcccaggctc
agatctggtctaaccagagagacccagtacaagcaaaaagcagatcttgtcttcgttgggagtgagctagcccttccagtcccccc
ttttcttttaaaaagtggctaagatctacagctgccttgtaagtcattggtcttaaaggtaccatttaaatctcgtagcacgtgtc
agtcctgctcctcggccacgaagtgcacgcagttgccggccgggtcgcgcagggcgaactcccgcccccacggctgctcgccgatc
tcggtcatggccggcccggaggcgtcccggaagttcgtggacacgacctccgaccactcggcgtacagctcgtccaggccgcgcac
ccacacccaggccagggtgttgtccggcaccacctggtcctggaccgcgctgatgaacagggtcacgtcgtcccggaccacaccgg
cgaagtcgtcctccacgaagtcccgggagaacccgagccggtcggtccagaactcgaccgctccggcgacgtcgcgcgcggtgagc
accggaacggcactggtcaacttggccatggtttagttcctcaccttgtcgtattatactatgccgatatactatgccgatgatta
attgtcaacaggggggagctttttgcaaaagcctaggcctccaaaaaagcctcctcactacttctggaatagctcagaggcagaggc
ggcctcggcctctgcataaataaaaaaaattagtcagccatggggcggagaatgggcggaactgggcggagttaggggcgggatgg
gcggagttaggggcgggactatggttgctgactaattgagatgcatgctttgcatacttctgcctgctggggagcctggggacttt
ccacacctggttgctgactaattgagatgcatgctttgcatacttctgcctgctggggagcctggggactttccacaccctaactg
acacacattccacagaattaattccaaactcattactaaccggatatctgcagaattccaggcggggaggcggcccaaagggagat
ccgactcgtctgagggcgaaggcgaagacgcggaagaggccgcagagccggcagcaggccgcgggaaggaaggtccgctggattga
gggccgaagggacgtagcagaaggacgtcccgcgcagaatccaggtggcaacacaggcgagcagccatggaaaggacgtcagcttc
cccgacaacaccacggaattgtcagtgcccaacagccgagcccctgtccagcagcgggcaaggcaggcggcgatgagttccgccgt
ggcaatagggagggggaaagcgaaagtcccggaaaggagctgacaggtggtggcaatgccccaaccagtggggggttgcgtcagcaa
acacagtgcacaccacgccacgttgcctgacaacgggccacaactcctcataaagagacagcaaccaggatttatacaaggaggag
aaaatgaaagccatacgggaagcaatagcatgatacaaaggcattaaagcagcgtatccacatagcgtaaaaggagcaacatagtt
aagaataccagtcaatctttcacaaattttgtaatccagaggttgattgtcgagcggccgccactgtgctggatatcaaccacttt
gtacaagaaagctgggtcgggtggctcaggctgctgaaccagttcttgcaggcgctgttcagatcttcctcggagatcagcttctg
ctcgtcgcccttgtacagttcatccatgcccatcacgtcagtaaaagccttttgccactctttaaagttcagctctgttttgctat
gtttgagttcggttttccggaaca

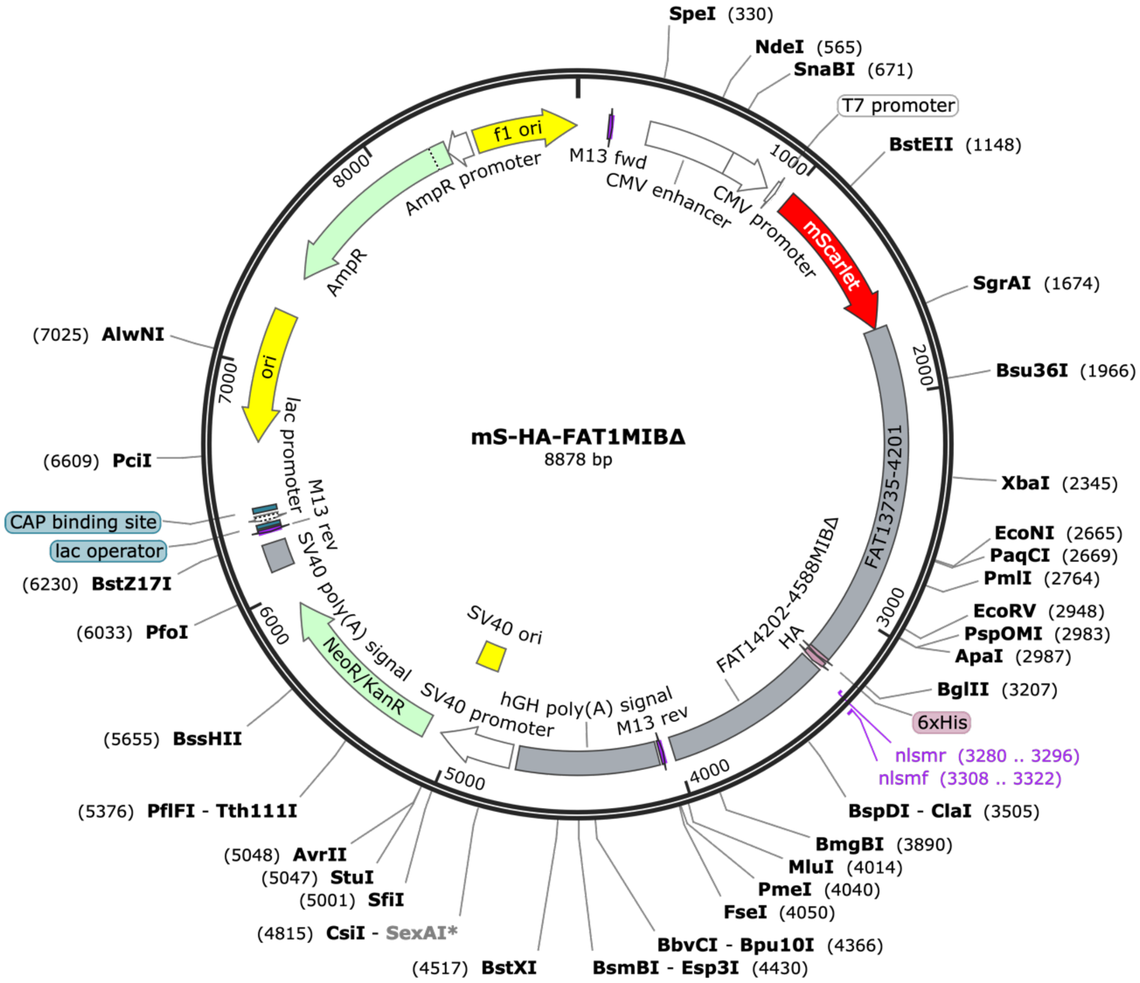

>mScarlet-HA-FAT1$^{3735-4588}$MIBΔ, 8878bps

ATTCGCCATTCAGGCTGCGCAACTGTTGGGAAGGGCGATCGGTGCGGGCCTCTTCGCTATTACGCCAGCTGGCGAAAGGGGGATGT
GCTGCAAGGCGATTAAGTTGGGTAACGCCAGGGTTTTCCCAGTCACGACGTTGTAAAACGACGGCCAGTGCCAAGCTGATCTATAC
ATTGAATCAATATTGGCAATTAGCCATATTAGTCATTGGTTATATAGCATAAATCAATATTGGCTATTGGCCATTGCATACGTTGT
ATCTATATCATAATATGTACATTTATATTGGCTCATGTCCAATATGACCGCCATGTTGACATTGATTATTGACTAGTTATTAATAG
TAATCAATTACGGGGTCATTAGTTCATAGCCCATATATGGAGTTCCGCGTTACATAACTTACGGTAAATGGCCCGCCTGGCTGACC
GCCCAACGACCCCCGCCCATTGACGTCAATAATGACGTATGTTCCCATAGTAACGCCAATAGGGACTTTCCATTGACGTCAATGGG
TGGAGTATTTACGGTAAACTGCCCACTTGGCAGTACATCAAGTGTATCATATGCCAAGTCCGCCCCCTATTGACGTCAATGACGGT
AAATGGCCCGCCTGGCATTATGCCCAGTACATGACCTTACGGGACTTTCCTACTTGGCAGTACATCTACGTATTAGTCATCGCTAT
TACCATGGTGATGCGGTTTTGGCAGTACACCAATGGGCGTGGATAGCGGTTTGACTCACGGGGATTTCCAAGTCTCCACCCCATTG
ACGTCAATGGGAGTTTGTTTTGGCACCAAAATCAACGGGACTTTCCAAAATGTCGTAATAACCCCGCCCCGTTGACGCAAATGGGC
GGTAGGCGTGTACGGTGGGAGGTCTATATAAGCAGAGCTCGTTTAGTGAACCGTCAGAATTTTGTAATACGACTCACTATAGGGCG
GCCGGGAATTCGTCGACTGGATCCGGTACGGTAGTATCAGCTAAGCCTGGTACCACCAATGGTGAGCAAGGGCGAGGCAGTGATCA
AGGAGTTCATGCGGTTCAAGGTGCACATGGAGGGCTCCATGAACGGCCACGAGTTCGAGATCGAGGGCGAGGGCGAGGGCCGCCCC
TACGAGGGCACCCAGACCGCCAAGCTGAAGGTGACCAAGGGTGGCCCCCTGCCCTTCTCCTGGGACATCCTGTCCCCTCAGTTCAT
GTACGGCTCCAGGGCCTTCACCAAGCACCCCGCCGACATCCCCGACTACTATAAGCAGTCCTTCCCCGAGGGCTTCAAGTGGGAGC
GCGTGATGAACTTCGAGGACGGCGGCGCCGTGACCGTGACCCAGGACACCTCCCTGGAGGACGGCACCCTGATCTACAAGGTGAAG
CTCCGCGGCACCAACTTCCCTCCTGACGGCCCCGTAATGCAGAAGAAGACAATGGGCTGGGAAGCGTCCACCGAGCGGTTGTACCC
CGAGGACGGCGTGCTGAAGGGCGACATTAAGATGGCCCTGCGCCTGAAGGACGGCGGCCGCTACCTGGCGGACTTCAAGACCACCT
ACAAGGCCAAGAAGCCCGTGCAGATGCCCGGCGCCTACAACGTCGACCGCAAGTTGGACATCACCTCCCACAACGAGGACTACACC
GTGGTGGAACAGTACGAACGCTCCGAGGGCCGCCACTCCACCGGCGGCATGGACGAGCTGTACAAGCCCTGGAGATTTTCATCACC
TCTTCTGGAGAAGAATACTCAGGTGGCGTCATTGGGAAGATCCATGCCACAGAAACTCTGCGCGGACTGGACTGCCCCTGGAAGT
TCTGCGATGAAAAGGTGTCTGTGGATGAAAGTGTGATGTCAACACACAGCACAGCCAGACTGAGTTTTGTGACTCCCCGCCACCAC
AGGGCAGCGGTGTGTCTCTGCAAAGAGGGAAGGTGCCCACCTGTCCACCATGGCTGTGAAGATGATCCGTGCCCTGAGGGATCCGA
ATGTGTGTCTGATCCCTGGGAGGAGAAACACACCTGTGTCTGTCCCAGCGGCAGGTTTGGTCAGTGCCCAGGGAGTTCATCTATGA
CACTGACTGGAAACAGCTACGTGAAATACCGTCTGACGGAAAATGAAAACAAATTAGAGATGAAACTGACCATGAGGCTCAGAACA
TATTCCACGCATGCGGTTGTCATGTATGCTCGAGGAACTGACTATAGCATCTTGGAGATTCATCATGGAAGGCTGCAGTACAAGTT
TGACTGTGGAAGTGGCCCTGGAATTGTCTCTGTTCAGAGCATTCAGGTCAATGATGGGCAGTGGCACGCAGTGGCCCTGGAAGTGA
ATGGAAACTATGCTCGCTTGGTTCTAGACCAAGTTCATACTGCATCGGGCACAGCCCCAGGGACTCTGAAAACCCTGAACCTGGAT
AACTATGTGTTTTTTGGTGGCCACATCCGTCAGCAGGGAACAAGGCATGGAAGAAGTCCTCAAGTTGGTAATGGTTTCAGGGGTTG
TATGGACTCCATTTATTTGAATGGGCAGGAGCTCCCTTTAAACAGCAAACCCAGAAGCTATGCACACATCGAAGAGTCGGTGGATG
TATCTCCAGGCTGCTTCCTGACGGCCACGGAAGACTGCGCCAGCAACCCTTGCCAGAATGGAGGCGTTTGCAATCCGTCACCTGCT
GGAGGTTATTACTGCAAATGCAGTGCCTTGTACATAGGGACCCACTGTGAGATAAGCGTCAATCCGTGTTCCTCCAAGCCATGCCT

CTATGGGGGCACGTGTGTTGTCGACAACGGAGGCTTTGTTTGCCAGTGTAGAGGATTATATACTGGTCAGAGGTGTCAGCTTAGTC
CATACTGCAAAGATGAACCCTGTAAGAATGGCGGAACATGCTTTGACAGTTTGGATGGCGCCGTTTGTCAGTGTGATTCGGGTTTT
AGGGGAGAAAGGTGTCAGAGTGATATCGACGAGTGCTCTGGAAACCCTTGCCTGCACGGGGCCCTCTGTGAGAACACGCACGGCTC
CTATCACTGCAACTGCAGCCACGAGTACAGGGGACGTCACTGCGAGGATGCTGCGCCCAACCAGTATGTGTCCACGCCGTGGAACA
TTGGGTTGGCGGAAGGAATTGGAATCGTTGTGTTTGTTGCAGGGATATTTTTACTGGTGGTGGTGTTTGTTCTCTTGGTACCGGTA
GTATCAGCTAAGCCTGTACCGAGGAGATCTGCCACCATGCATCACCATCACCATCACGATTACCCATACGATGTTCCAGATTACGC
TGCGATCGTGTGCCGTAAGATGATTAGTCGGAAAAAGAAGCATCAGGCTGAACCTAAAGACAAGCACCTGGGACCCGCTACGGCTT
TCTTGCAAAGACCGTATTTTGATTCCAAGCTAAATAAGAACATTTACTCAGACATACCACCCCAGGTGCCTGTCCGGCCTATTTCC
TACACCCCGAGTTATGAGGTGATTGATGAGCAGACACCCCTGTACTCAGCAGATCCAAACGCCATCGATACGGACTATTACCCTGG
AGGCTACGACATCGAAAGTGATTTTCCTCCACCCCCAGAAGACTTCCCCGCAGCTGATGAGCTACCACCGTTACCGCCCGAATTCA
GCAATCAGTTTGAATCCATCCACCCTCCTAGAGACATGCCTGCCGCGGGTAGCTTGGGTTCTTCATCAAGAAACCGGCAGAGGTTC
AACTTGAATCAGTATTTGCCCAATTTTTATCCCCTCGATATGTCTGAACCTCAAACAAAAGGCACTGGTGAGAATAGTACTTGTAG
AGAACCCCATGCCCCTTACCCGCCAGGGTATCAAAGACACTTCGAGGCGCCCGCTGTCGAGAGCATGCCCATGTCTGTGTACGCCT
CCACCGCCTCCTGCTCTGACGTGTCAGCCTGCTGCGAAGTGGAGTCCGAGGTCATGATGAGTGACTATGAGAGCGGGGACGACGGC
CACTTCGAAGAGGTGACGATCCCGCCCCTGGATTCCCAGCAGCACACGGAAGTCTGAACGCGTGAGCGGCCGCACTCGAGGTTTAA
ACGGCCGGCCGCGGTCATAGCTGTTTCCTGAACAGATCCCGGGTGGCATCCCTGTGACCCCTCCCCAGTGCCTCTCCTGGCCCTGG
AAGTTGCCACTCCAGTGCCCACCAGCCTTGTCCTAATAAAATTAAGTTGCATCATTTTGTCTGACTAGGTGTCCTTCTATAATATT
ATGGGGTGGAGGGGGGTGGTATGGAGCAAGGGGCAAGTTGGGAAGACAACCTGTAGGGCCTGCGGGGTCTATTGGGAACCAAGCTG
GAGTGCAGTGGCACAATCTTGGCTCACTGCAATCTCCGCCTCCTGGGTTCAAGCGATTCTCCTGCCTCAGCCTCCCGAGTTGTTGG
GATTCCAGGCATGCATGACCAGGCTCAGCTAATTTTTGTTTTTTTGGTAGAGACGGGGTTTCACCATATTGGCCAGGCTGGTCTCC
AACTCCTAATCTCAGGTGATCTACCCACCTTGGCCTCCCAAATTGCTGGGATTACAGGCGTGAACCACTGCTCCCTTCCCTGTCCT
TCTGATTTTAAAATAACTATACCAGCAGGAGGACGTCCAGACACAGCATAGGCTACCTGGCCATGCCCAACCGGTGGGACATTTGA
GTTGCTTGCTTGGCACTGTCCTCTCATGCGTTGGGTCCACTCAGTAGATGCCTGTTGAATTGGGTACGCGGCCAGCTTGGCTGTGG
AATGTGTGTCAGTTAGGGTGTGGAAAGTCCCCAGGCTCCCCAGCAGGCAGAAGTATGCAAAGCATGCATCTCAATTAGTCAGCAAC
CAGGTGTGGAAAGTCCCCAGGCTCCCCAGCAGGCAGAAGTATGCAAAGCATGCATCTCAATTAGTCAGCAACCATAGTCCCGCCCC
TAACTCCGCCCATCCCGCCCCTAACTCCGCCCAGTTCCGCCCATTCTCCGCCCCATGGCTGACTAATTTTTTTTATTTATGCAGAG
GCCGAGGCCGCCTCGGCCTCTGAGCTATTCCAGAAGTAGTGAGGAGGCTTTTTTGGAGGCCTAGGCTTTTGCAAAAAGCTCCCGGG
AGCTTGTATATCCATTTTCGGATCTGATCAAGAGACAGGATGAGGATCGTTTCGCATGATTGAACAAGATGGATTGCACGCAGGTT
CTCCGGCCGCTTGGGTGGAGAGGCTATTCGGCTATGACTGGGCACAACAGACAATCGGCTGCTCTGATGCCGCCGTGTTCCGGCTG
TCAGCGCAGGGGCGCCCGGTTCTTTTTGTCAAGACCGACCTGTCCGGTGCCCTGAATGAACTGCAGGACGAGGCAGCGCGGCTATC
GTGGCTGGCCACGACGGGCGTTCCTTGCGCAGCTGTGCTCGACGTTGTCACTGAAGCGGGAAGGGACTGGCTGCTATTGGGCGAAG
TGCCGGGGCAGGATCTCCTGTCATCTCACCTTGCTCCTGCCGAGAAAGTATCCATCATGGCTGATGCAATGCGGCGGCTGCATACG
CTTGATCCGGCTACCTGCCCATTCGACCACCAAGCGAAACATCGCATCGAGCGAGCACGTACTCGGATGGAAGCCGGTCTTGTCGA
TCAGGATGATCTGGACGAAGAGCATCAGGGGCTCGCGCCAGCCGAACTGTTCGCCAGGCTCAAGGCGCGCATGCCCGACGGCGAGG
ATCTCGTCGTGACCCATGGCGATGCCTGCTTGCCGAATATCATGGTGGAAAATGGCCGCTTTTCTGGATTCATCGACTGTGGCCGG
CTGGGTGTGGCCGACCGCTATCAGGACATAGCGTTGGCTACCCGTGATATTGCTGAAGAGCTTGGCGGCGAATGGGCTGACCGCTT
CCTCGTGCTTTACGGTATCGCCGCTCCCGATTCGCAGCGCATCGCCTTCTATCGCCTTCTTGACGAGTTCTTCTGAGCGGGACTCT
GGGGTTCGAAATGACCGACCAAGCGACGCCCAACCTGCCATCACGAGATTTCGATTCCACCGCCGCCTTCTATGAAAGGTTGGGCT
TCGGAATCGTTTTCCGGGACGCCGGCTGGATGATCCTCCAGCGCGGGGATCTCATGCTGGAGTTCTTCGCCCACCCCAACTTGTTT
ATTGCAGCTTATAATGGTTACAAATAAAGCAATAGCATCACAAATTTCACAAATAAAGCATTTTTTTCACTGCATTCTAGTTGTGG
TTTGTCCAAACTCATCAATGTATCTTATCATGTCTGTATACCGTCGACCTCTAGCTAGAGCTTGGCGTAATCATGGTCATAGCTGT
TTCCTGTGTGAAATTGTTATCCGCTCACAATTCCACACAACATACGAGCCGGAAGCATAAAGTGTAAAGCCTGGGGTGCCTAATGA
GTGAGCTAACTCACATTAATTGCGTTGCGCTCACTGCCCGCTTTCCAGTCGGGAAACCTGTCGTGCCAGCTGCATTAATGAATCGG
CCAACGCGCGGGGAGAGGCGGTTTGCGTATTGGGCGCTCTTCCGCTTCCTCGCTCACTGACTCGCTGCGCTCGGTCGTTCGGCTGC
GGCGAGCGGTATCAGCTCACTCAAAGGCGGTAATACGGTTATCCACAGAATCAGGGGATAACGCAGGAAAGAACATGTGAGCAAAA
GGCCAGCAAAAGGCCAGGAACCGTAAAAAGGCCGCGTTGCTGGCGTTTTTCCATAGGCTCCGCCCCCCTGACGAGCATCACAAAAA
TCGACGCTCAAGTCAGAGGTGGCGAAACCCGACAGGACTATAAAGATACCAGGCGTTTCCCCCTGGAAGCTCCCTCGTGCGCTCTC
CTGTTCCGACCCTGCCGCTTACCGGATACCTGTCCGCCTTTCTCCCTTCGGGAAGCGTGGCGCTTTCTCATAGCTCACGCTGTAGG
TATCTCAGTTCGGTGTAGGTCGTTCGCTCCAAGCTGGGCTGTGTGCACGAACCCCCCGTTCAGCCCGACCGCTGCGCCTTATCCGG
TAACTATCGTCTTGAGTCCAACCCGGTAAGACACGACTTATCGCCACTGGCAGCAGCCACTGGTAACAGGATTAGCAGAGCGAGGT
ATGTAGGCGGTGCTACAGAGTTCTTGAAGTGGTGGCCTAACTACGGCTACACTAGAAGAACAGTATTTGGTATCTGCGCTCTGCTG
AAGCCAGTTACCTTCGGAAAAAGAGTTGGTAGCTCTTGATCCGGCAAACAAACCACCGCTGGTAGCGGTGGTTTTTTTGTTTGCAA
GCAGCAGATTACGCGCAGAAAAAAAGGATCTCAAGAAGATCCTTTGATCTTTTCTACGGGGTCTGACGCTCAGTGGAACGAAAACT
CACGTTAAGGGATTTTGGTCATGAGATTATCAAAAAGGATCTTCACCTAGATCCTTTTAAATTAAAAATGAAGTTTTAAATCAATC
TAAAGTATATATGAGTAAACTTGGTCTGACAGTTACCAATGCTTAATCAGTGAGGCACCTATCTCAGCGATCTGTCTATTTCGTTC
ATCCATAGTTGCCTGACTCCCCGTCGTGTAGATAACTACGATACGGGAGGGCTTACCATCTGGCCCCAGTGCTGCAATGATACCGC
GAGACCCACGCTCACCGGCTCCAGATTTATCAGCAATAAACCAGCCAGCCGGAAGGGCCGAGCGCAGAAGTGGTCCTGCAACTTTA
TCCGCCTCCATCCAGTCTATTAATTGTTGCCGGGAAGCTAGAGTAAGTAGTTCGCCAGTTAATAGTTTGCGCAACGTTGTTGCCAT
TGCTACAGGCATCGTGGTGTCACGCTCGTCGTTTGGTATGGCTTCATTCAGCTCCGGTTCCCAACGATCAAGGCGAGTTACATGAT
CCCCCATGTTGTGCAAAAAAGCGGTTAGCTCCTTCGGTCCTCCGATCGTTGTCAGAAGTAAGTTGGCCGCAGTGTTATCACTCATG
GTTATGGCAGCACTGCATAATTCTCTTACTGTCATGCCATCCGTAAGATGCTTTTCTGTGACTGGTGAGTACTCAACCAAGTCATT
CTGAGAATAGTGTATGCGGCGACCGAGTTGCTCTTGCCCGGCGTCAATACGGGATAATACCGCGCCACATAGCAGAACTTTAAAAG
TGCTCATCATTGGAAAACGTTCTTCGGGGCGAAAACTCTCAAGGATCTTACCGCTGTTGAGATCCAGTTCGATGTAACCCACTCGT
GCACCCAACTGATCTTCAGCATCTTTTACTTTCACCAGCGTTTCTGGGTGAGCAAAAACAGGAAGGCAAAATGCCGCAAAAAAGGG
AATAAGGGCGACACGGAAATGTTGAATACTCATACTCTTCCTTTTTCAATATTATTGAAGCATTTATCAGGGTTATTGTCTCATGA
GCGGATACATATTTGAATGTATTTAGAAAAATAAACAAATAGGGGTTCCGCGCACATTTCCCCGAAAAGTGCCACCTGACGCGCCC
TGTAGCGGCGCATTAAGCGCGGCGGGTGTGGTGGTTACGCGCAGCGTGACCGCTACACTTGCCAGCGCCCTAGCGCCCGCTCCTTT
CGCTTTCTTCCCTTCCTTTCTCGCCACGTTCGCCGGCTTTCCCCGTCAAGCTCTAAATCGGGGGCTCCCTTTAGGGTTCCGATTTA
GTGCTTTACGGCACCTCGACCCCAAAAAACTTGATTAGGGTGATGGTTCACGTAGTGGGCCATCGCCCTGATAGACGGTTTTTCGC
CCTTTGACGTTGGAGTCCACGTTCTTTAATAGTGGACTCTTGTTCCAAACTGGAACAACACTCAACCCTATCTCGGTCTATTCTTT
TGATTTATAAGGGATTTTGCCGATTTCGGCCTATTGGTTAAAAAATGAGCTGATTTAACAAAAATTTAACGCGAATTTTAACAAAA
TATTAACGCTTACAATTTCC

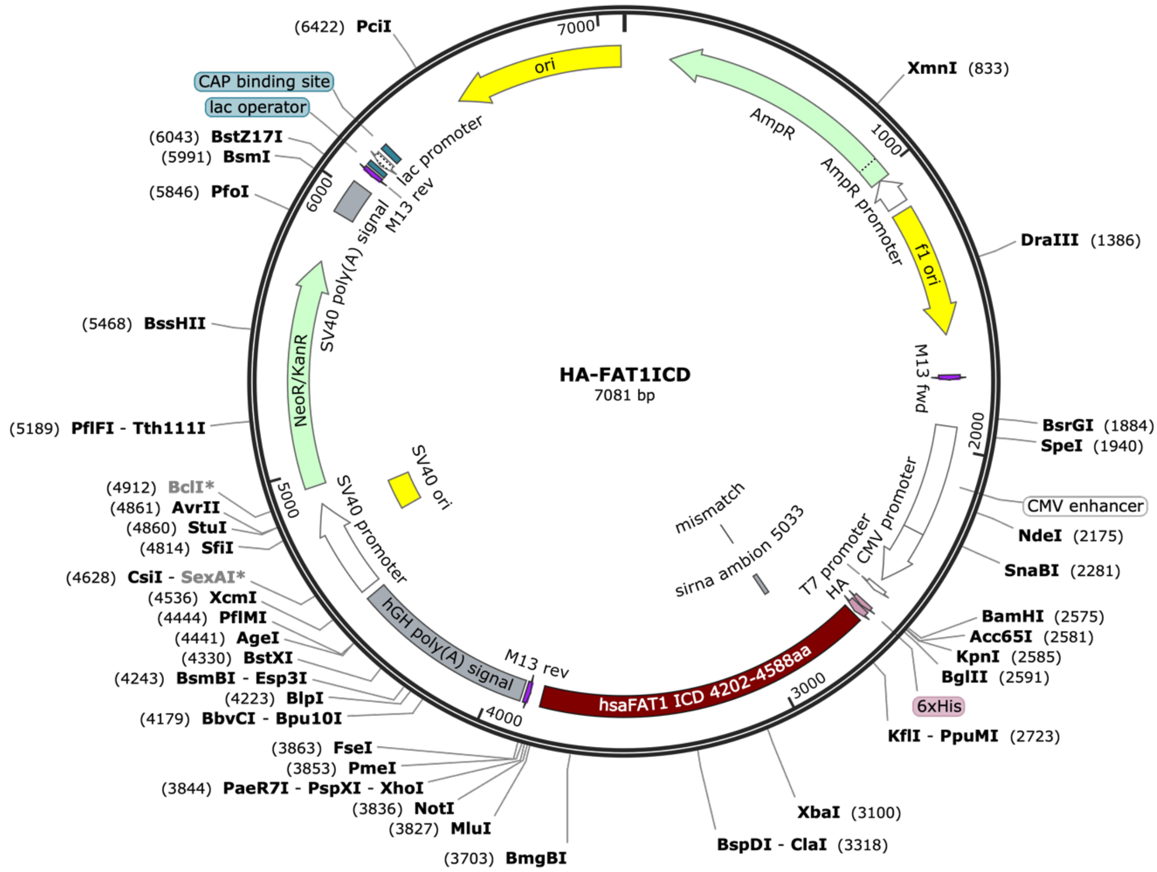

> HA-FAT1 $^{4202-4588}$ ICD, 7081bps

TGATCTTTTCTACGGGGTCTGACGCTCAGTGGAACGAAAACTCACGTTAAGGGATTTTGGTCATGAGATTATCAAAAAGGATCTTC
ACCTAGATCCTTTTAAATTAAAAATGAAGTTTTAAATCAATCTAAAGTATATATGAGTAAACTTGGTCTGACAGTTACCAATGCTT
AATCAGTGAGGCACCTATCTCAGCGATCTGTCTATTTCGTTCATCCATAGTTGCCTGACTCCCCGTCGTGTAGATAACTACGATAC
GGGAGGGCTTACCATCTGGCCCCAGTGCTGCAATGATACCGTGAGACCCACGCTCACCGGCTCCAGATTTATCAGCAATAAACCAG
CCAGCCGGAAGGGCCGAGCGCAGAAGTGGTCCTGCAACTTTATCCGCCTCCATCCAGTCTATTAATTGTTGCCGGGAAGCTAGAGT
AAGTAGTTCGCCAGTTAATAGTTTGCGCAACGTTGTTGCCATTGCTACAGGCATCGTGGTGTCACGCTCGTCGTTTGGTATGGCTT
CATTCAGCTCCGGTTCCCAACGATCAAGGCGAGTTACATGATCCCCCATGTTGTGCAAAAAAGCGGTTAGCTCCTTCGGTCCTCCG
ATCGTTGTCAGAAGTAAGTTGGCCGCAGTGTTATCACTCATGGTTATGGCAGCACTGCATAATTCTCTTACTGTCATGCCATCCGT
AAGATGCTTTTCTGTGACTGGTGAGTACTCAACCAAGTCATTCTGAGAATAGTGTATGCGGCGACCGAGTTGCTCTTGCCCGGCGT
CAATACGGGATAATACCGCGCCACATAGCAGAACTTTAAAAGTGCTCATCATTGGAAAACGTTCTTCGGGGCGAAAACTCTCAAGG
ATCTTACCGCTGTTGAGATCCAGTTCGATGTAACCCACTCGTGCACCCAACTGATCTTCAGCATCTTTTACTTTCACCAGCGTTTC
TGGGTGAGCAAAAACAGGAAGGCAAAATGCCGCAAAAAAGGGAATAAGGGCGACACGGAAATGTTGAATACTCATACTCTTCCTTT
TTCAATATTATTGAAGCATTTATCAGGGTTATTGTCTCATGAGCGGATACATATTTGAATGTATTTAGAAAAATAAACAAATAGGG
GTTCCGCGCACATTTCCCCGAAAAGTGCCACCTGACGCGCCCTGTAGCGGCGCATTAAGCGCGGCGGGTGTGGTGGTTACGCGCAG
CGTGACCGCTACACTTGCCAGCGCCCTAGCGCCCGCTCCTTTCGCTTTCTTCCCTTCCTTTCTCGCCACGTTCGCCGGCTTTCCCC
GTCAAGCTCTAAATCGGGGGCTCCCTTTAGGGTTCCGATTTAGTGCTTTACGGCACCTCGACCCCAAAAAACTTGATTAGGGTGAT
GGTTCACGTAGTGGGCCATCGCCCTGATAGACGGTTTTTCGCCCTTTGACGTTGGAGTCCACGTTCTTTAATAGTGGACTCTTGTT
CCAAACTGGAACAACACTCAACCCTATCTCGGTCTATTCTTTTGATTTATAAGGGATTTTGCCGATTTCGGCCTATTGGTTAAAAA
ATGAGCTGATTTAACAAAAATTTAACGCGAATTTTAACAAAATATTAACGCTTACAATTTCCATTCGCCATTCAGGCTGCGCAACT
GTTGGGAAGGGCGATCGGTGCGGGCCTCTTCGCTATTACGCCAGCTGGCGAAAGGGGGATGTGCTGCAAGGCGATTAAGTTGGGTA
ACGCCAGGGTTTTCCCAGTCACGACGTTGTAAAACGACGGCCAGTGCCAAGCTGATCTATACATTGAATCAATATTGGCAATTAGC
CATATTAGTCATTGGTTATATAGCATAAATCAATATTGGCTATTGGCCATTGCATACGTTGTATCTATATCATAATATGTACATTT
ATATTGGCTCATGTCCAATATGACCGCCATGTTGACATTGATTATTGACTAGTTATTAATAGTAATCAATTACGGGGTCATTAGTT
CATAGCCCATATATGGAGTTCCGCGTTACATAACTTACGGTAAATGGCCCGCCTGGCTGACCGCCCAACGACCCCCGCCCATTGAC
GTCAATAATGACGTATGTTCCCATAGTAACGCCAATAGGGACTTTCCATTGACGTCAATGGGTGGAGTATTTACGGTAAACTGCCC
ACTTGGCAGTACATCAAGTGTATCATATGCCAAGTCCGCCCCCTATTGACGTCAATGACGGTAAATGGCCCGCCTGGCATTATGCC
CAGTACATGACCTTACGGGACTTTCCTACTTGGCAGTACATCTACGTATTAGTCATCGCTATTACCATGGTGATGCGGTTTTGGCA
GTACACCAATGGGCGTGGATAGCGGTTTGACTCACGGGGATTTCCAAGTCTCCACCCCATTGACGTCAATGGGAGTTTGTTTTGGC
ACCAAAATCAACGGGACTTTCCAAAATGTCGTAATAACCCCGCCCCGTTGACGCAAATGGGCGGTAGGCGTGTACGGTGGGAGGTC
TATATAAGCAGAGCTCGTTTAGTGAACCGTCAGAATTTTGTAATACGACTCACTATAGGGCGGCCGGGAATTCGTCGACTGGATCC
GGTACCGAGGAGATCTGCCACCATGCATCACCATCACCATCACGATTACCCATACGATGTTCCAGATTACGCTGCGATCGTGTGCC
GTAAGATGATTAGTCGGAAAAAGAAGCATCAGGCTGAACCTAAAGACAAGCACCTGGGACCCGCTACGGCTTTCTTGCAAAGACCG
TATTTTGATTCCAAGCTAAATAAGAACATTTACTCAGACATACCACCCCAGGTGCCTGTCCGGCCTATTTCCTACACCCCGAGTAT
TCCAAGTGACTCAAGAAACAATCTGGACCGAAATAGTTTTGAAGGATCTGCTATCCCAGAGCATCCCGAATTCAGCACTTTTAACC
CCGAGTCTGTGCACGGGCACCGAAAAGCAGTGGCGGTCTGCAGCGTGGCGCCAAACCTGCCTCCCCCACCCCCTTCAAACTCCCCT
TCTGACAGCGACTCCATCCAGAAGCCTAGCTGGGACTTTGACTATGACACAAAAGTGGTGGATCTTGATCCCTGTCTTTCCAAGAA

GCCTCTAGAGGAAAAGCCTTCCCAGCCATACAGTGCCCGGGAAAGCCTGTCTGAAGTGCAGTCTCTGAGCTCCTTCCAGTCCGAAT
CGTGCGATGACAATGGGTATCACTGGGATACATCAGATTGGATGCCAAGCGTTCCTCTGCCGGACATACAAGAGTTCCCCAACTAT
GAGGTGATTGATGAGCAGACACCCCTGTACTCAGCAGATCCAAACGCCATCGATACGGACTATTACCCTGGAGGCTACGACATCGA
AAGTGATTTTCCTCCACCCCCAGAAGACTTCCCCGCAGCTGATGAGCTACCACCGTTACCGCCCGAATTCAGCAATCAGTTTGAAT
CCATCCACCCTCCTAGAGACATGCCTGCCGCGGGTAGCTTGGGTTCTTCATCAAGAAACCGGCAGAGGTTCAACTTGAATCAGTAT
TTGCCCAATTTTTATCCCCTCGATATGTCTGAACCTCAAACAAAAGGCACTGGTGAGAATAGTACTTGTAGAGAACCCCATGCCCC
TTACCCGCCAGGGTATCAAAGACACTTCGAGGCGCCCGCTGTCGAGAGCATGCCCATGTCTGTGTACGCCTCCACCGCCTCCTGCT
CTGACGTGTCAGCCTGCTGCGAAGTGGAGTCCGAGGTCATGATGAGTGACTATGAGAGCGGGGACGACGGCCACTTCGAAGAGGTG
ACGATCCCGCCCCTGGATTCCCAGCAGCACACGGAAGTCTGAACGCGTGAGCGGCCGCACTCGAGGTTTAAACGGCCGGCCGCGGT
CATAGCTGTTTCCTGAACAGATCCCGGGTGGCATCCCTGTGACCCCTCCCCAGTGCCTCTCCTGGCCCTGGAAGTTGCCACTCCAG
TGCCCACCAGCCTTGTCCTAATAAAATTAAGTTGCATCATTTTGTCTGACTAGGTGTCCTTCTATAATATTATGGGGTGGAGGGGG
GTGGTATGGAGCAAGGGGCAAGTTGGGAAGACAACCTGTAGGGCCTGCGGGGTCTATTGGGAACCAAGCTGGAGTGCAGTGGCACA
ATCTTGGCTCACTGCAATCTCCGCCTCCTGGGTTCAAGCGATTCTCCTGCCTCAGCCTCCCGAGTTGTTGGGATTCCAGGCATGCA
TGACCAGGCTCAGCTAATTTTTGTTTTTTTGGTAGAGACGGGGTTTCACCATATTGGCCAGGCTGGTCTCCAACTCCTAATCTCAG
GTGATCTACCCACCTTGGCCTCCCAAATTGCTGGGATTACAGGCGTGAACCACTGCTCCCTTCCCTGTCCTTCTGATTTTAAAATA
ACTATACCAGCAGGAGGACGTCCAGACACAGCATAGGCTACCTGGCCATGCCCAACCGGTGGGACATTTGAGTTGCTTGCTTGGCA
CTGTCCTCTCATGCGTTGGGTCCACTCAGTAGATGCCTGTTGAATTGGGTACGCGGCCAGCTTGGCTGTGGAATGTGTGTCAGTTA
GGGTGTGGAAAGTCCCCAGGCTCCCCAGCAGGCAGAAGTATGCAAAGCATGCATCTCAATTAGTCAACAACCAGGTGTGGAAAGTC
CCCAGGCTCCCCAGCAGGCAGAAGTATGCAAAGCATGCATCTCAATTAGTCAGCAACCATAGTCCCGCCCCTAACTCCGCCCATCC
CGCCCCTAACTCCGCCCAGTTCCGCCCATTCTCCGCCCCATGGCTGACTAATTTTTTTTATTTATGCAGAGGCCGAGGCCGCCTCG
GCCTCTGAGCTATTCCAGAAGTAGTGAGGAGGCTTTTTTGGAGGCCTAGGCTTTTGCAAAAAGCTCCCGGGAGCTTGTATATCCAT
TTTCGGATCTGATCAAGAGACAGGATGAGGATCGTTTCGCATGATTGAACAAGATGGATTGCACGCAGGTTCTCCGGCCGCTTGGG
TGGAGAGGCTATTCGGCTATGACTGGGCACAACAGACAATCGGCTGCTCTGATGCCGCCGTGTTCCGGCTGTCAGCGCAGGGGCGC
CCGGTTCTTTTTGTCAAGACCGACCTGTCCGGTGCCCTGAATGAACTGCAGGACGAGGCAGCGCGGCTATCGTGGCTGGCCACGAC
GGGCGTTCCTTGCGCAGCTGTGCTCGACGTTGTCACTGAAGCGGGAAGGGACTGGCTGCTATTGGGCGAAGTGCCGGGGCAGGATC
TCCTGTCATCTCACCTTGCTCCTGCCGAGAAAGTATCCATCATGGCTGATGCAATGCGGCGGCTGCATACGCTTGATCCGGCTACC
TGCCCATTCGACCACCAAGCGAAACATCGCATCGAGCGAGCACGTACTCGGATGGAAGCCGGTCTTGTCGATCAGGATGATCTGGA
CGAAGAGCATCAGGGGCTCGCGCCAGCCGAACTGTTCGCCAGGCTCAAGGCGCGCATGCCCGACGGCGAGGATCTCGTCGTGACCC
ATGGCGATGCCTGCTTGCCGAATATCATGGTGGAAAATGGCCGCTTTTCTGGATTCATCGACTGTGGCCGGCTGGGTGTGGCCGAC
CGCTATCAGGACATAGCGTTGGCTACCCGTGATATTGCTGAAGAGCTTGGCGGCGAATGGGCTGACCGCTTCCTCGTGCTTTACGG
TATCGCCGCTCCCGATTCGCAGCGCATCGCCTTCTATCGCCTTCTTGACGAGTTCTTCTGAGCGGGACTCTGGGGTTCGAAATGAC
CGACCAAGCGACGCCCAACCTGCCATCACGAGATTTCGATTCCACCGCCGCCTTCTATGAAAGGTTGGGCTTCGGAATCGTTTTCC
GGGACGCCGGCTGGATGATCCTCCAGCGCGGGGATCTCATGCTGGAGTTCTTCGCCCACCCCAACTTGTTTATTGCAGCTTATAAT
GGTTACAAATAAAGCAATAGCATCACAAATTTCACAAATAAAGCATTTTTTTCACTGCATTCTAGTTGTGGTTTGTCCAAACTCAT
CAATGTATCTTATCATGTCTGTATACCGTCGACCTCTAGCTAGAGCTTGGCGTAATCATGGTCATAGCTGTTTCCTGTGTGAAATT
GTTATCCGCTCACAATTCCACACAACATACGAGCCGGAAGCATAAAGTGTAAAGCCTGGGGTGCCTAATGAGTGAGCTAACTCACA
TTAATTGCGTTGCGCTCACTGCCCGCTTTCCAGTCGGGAAACCTGTCGTGCCAGCTGCATTAATGAATCGGCCAACGCGCGGGGAG
AGGCGGTTTGCGTATTGGGCGCTCTTCCGCTTCCTCGCTCACTGACTCGCTGCGCTCGGTCGTTCGGCTGCGGCGAGCGGTATCAG
CTCACTCAAAGGCGGTAATACGGTTATCCACAGAATCAGGGGATAACGCAGGAAAGAACATGTGAGCAAAAGGCCAGCAAAAGGCC
AGGAACCGTAAAAAGGCCGCGTTGCTGGCGTTTTTCCATAGGCTCCGCCCCCCTGACGAGCATCACAAAAATCGACGCTCAAGTCA
GAGGTGGCGAAACCCGACAGGACTATAAAGATACCAGGCGTTTCCCCCTGGAAGCTCCCTCGTGCGCTCTCCTGTTCCGACCCTGC
CGCTTACCGGATACCTGTCCGCCTTTCTCCCTTCGGGAAGCGTGGCGCTTTCTCATAGCTCACGCTGTAGGTATCTCAGTTCGGTG
TAGGTCGTTCGCTCCAAGCTGGGCTGTGTGCACGAACCCCCCGTTCAGCCCGACCGCTGCGCCTTATCCGGTAACTATCGTCTTGA
GTCCAACCCGGTAAGACACGACTTATCGCCACTGGCAGCAGCCACTGGTAACAGGATTAGCAGAGCGAGGTATGTAGGCGGTGCTA
CAGAGTTCTTGAAGTGGTGGCCTAACTACGGCTACACTAGAAGAACAGTATTTGGTATCTGCGCTCTGCTGAAGCCAGTTACCTTC
GGAAAAAGAGTTGGTAGCTCTTGATCCGGCAAACAAACCACCGCTGGTAGCGGTGGTTTTTTTGTTTGCAAGCAGCAGATTACGCG
CAGAAAAAAAGGATCTCAAGAAGATCCTT

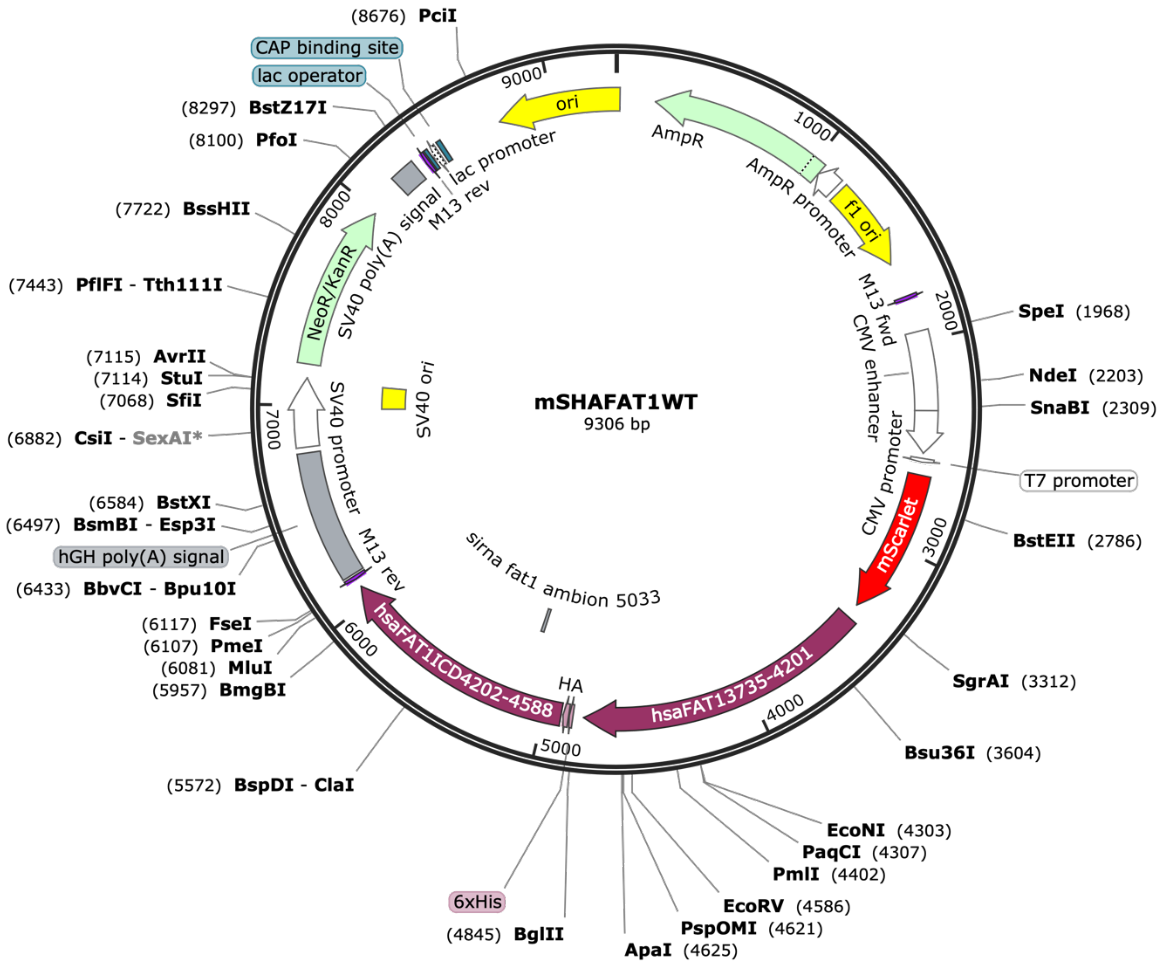

>mScarlet-HA-FAT1$^{3735-4588}$WT, 9306bps

CAGAAAAAAAGGATCTCAAGAAGATCCTTTGATCTTTTCTACGGGGTCTGATGCTCAGTGGAACGAAAACTCACGTTAAGGGATTT
TGGTCATGAGATTATCAAAAAGGATCTTCACCTAGATCCTTTTAAATTAAAAATGAAGTTTTAAATCAATCTAAAGTATATATGAG
TAAACTTGGTCTGACAGTTACCAATGCTTAATCAGTGAGGCACCTATCTCAGCGATCTGTCTATTTCGTTCATCCATAGTTGCCTG
ACTCCCCGTCGTGTAGATAACTACGATACGGGAGGGCTTACCATCTGGCCCCAGTGCTGCAATGATACCGCGAGACCCACGCTCAC
CGGCTCCAGATTTATCAGCAATAAACCAGCCAGCCGGAAGGGCCGAGCGCAGAAGTGGTCCTGCAACTTTATCCGCCTCCATCCAG
TCTATTAATTGTTGCCGGGAAGCTAGAGTAAGTAGTTCGCCAGTTAATAGTTTGCGCAACGTTGTTGCCATTGCTACAGGCATCGT
GGTGTCACGCTCGTCGTTTGGTATGGCTTCATTCAGCTCCGGTTCCCAACGATCAAGGCGAGTTACATGATCCCCCATGTTGTGCA
AAAAAGCGGTTAGCTCCTTCGGTCCTCCGATCGTTGTCAGAAGTAAGTTGGCCGCAGTGTTATCACTCATGGTTATGGCAGCACTG
CATAATTCTCTTACTGTCATGCCATCCGTAAGATGCTTTTCTGTGACTGGTGAGTACTCAACCAAGTCATTCTGAGAATAGTGTAT
GCGGCGACCGAGTTGCTCTTGCCCGGCGTCAATACGGGATAATACCGCGCCACATAGCAGAACTTTAAAAGTGCTCATCATTGGAA
AACGTTCTTCGGGGCGAAAACTCTCAAGGATCTTACCGCTGTTGAGATCCAGTTCGATGTAACCCACTCGTGCACCCAACTGATCT
TCAGCATCTTTTACTTTCACCAGCGTTTCTGGGTGAGCAAAAACAGGAAGGCAAAATGCCGCAAAAAAGGGAATAAGGGCGACACG
GAAATGTTGAATACTCATACTCTTCCTTTTTCAATATTATTGAAGCATTTATCAGGGTTATTGTCTCATGAGCGGATACATATTTG
AATGTATTTAGAAAAATAAACAAATAGGGGTTCCGCGCACATTTCCCCGAAAAGTGCCACCTGACGCGCCCGTAGCGGCGCATTAA
GCGCGGCGGGTGTGGTGGTTACGCGCAGCGTGACCGCTACACTTGCCAGCGCCCTAGCGCCCGCTCCTTTCGCTTTCTTCCCTTCC
TTTCTCGCCACGTTCGCCGGCTTTCCCCGTCAAGCTCTAAATCGGGGGCTCCCTTTAGGGTTCCGATTTAGTGCTTTACGGCACCT
CGACCCCAAAAAACTTGATTAGGGTGATGGTTCACGTAGTGGGCCATCGCCCTGATAGACGGTTTTTCGCCCTTTGACGTTGGAGT
CCACGTTCTTTAATAGTGGACTCTTGTTCCAAACTGGAACAACACTCAACCCTATCTCGGTCTATTCTTTTGATTTATAAGGGATT
TTGCCGATTTCGGCCTATTGGTTAAAAAATGAGCTGATTTAACAAAAATTTAACGCGAATTTTAACAAAATATTAACGCTTACAAT
TTCCATTCGCCATTCAGGCTGCGCAACTGTTGGGAAGGGCGATCGGTGCGGGCCTCTTCGCTATTACGCCAGCTGGCGAAAGGGGG
ATGTGCTGCAAGGCGATTAAGTTGGGTAACGCCAGGGTTTTCCCAGTCACGACGTTGTAAAACGACGGCCAGTGCCAAGCTGATCT
ATACATTGAATCAATATTGGCAATTAGCCATATTAGTCATTGGTTATATAGCATAAATCAATATTGGCTATTGGCCATTGCATACG
TTGTATCTATATCATAATATGTACATTTATATTGGCTCATGTCCAATATGACCGCCATGTTGACATTGATTATTGACTAGTTATTA
ATAGTAATCAATTACGGGGTCATTAGTTCATAGCCCATATATGGAGTTCCGCGTTACATAACTTACGGTAAATGGCCCGCCTGGCT
GACCGCCCAACGACCCCCGCCCATTGACGTCAATAATGACGTATGTTCCCATAGTAACGCCAATAGGGACTTTCCATTGACGTCAA
TGGGTGGAGTATTTACGGTAAACTGCCCACTTGGCAGTACATCAAGTGTATCATATGCCAAGTCCGCCCCCTATTGACGTCAATGA
CGGTAAATGGCCCGCCTGGCATTATGTCCAGTACATGACCTTACGGAACTTTCCTACTTGGCAGTACATCTACGTATTAGTCATCG
CTATTACCATGGTGATGCGGTTTTGGCAGTACACCAATGGGCGTGGATAGCGGTTTGACTCACGGGGATTTCCAAGTCTCCACCCC
ATTGACGTCAATGGGAGTTTGTTTTGGCACCAAAATCAACGGGACTTTCCAAAATGTCGTAATAACCCCGCCCCGTTGACGCAAAT
GGGCGGTAGGCGTGTACGGTGGGAGGTCTATATAAGCAGAGCTCGTTTAGTGAACCGTCAGAATTTTGTAATACGACTCACTATAG
GGCGGCCGGGAATTCGTCGACTGGATCCGGTACGGTAGTATCAGCTAAGCCTGGTACCACCAATGGTGAGCAAGGGCGAGGCAGTG
ATCAAGGAGTTCATGCGGTTCAAGGTGCACATGGAGGGCTCCATGAACGGCCACGAGTTCGAGATCGAGGGCGAGGGCGAGGGCCG
CCCCTACGAGGGCACCCAGACCGCCAAGCTGAAGGTGACCAAGGGTGGCCCCCTGCCCTTCTCCTGGGACATCCTGTCCCCTCAGT

TCATGTACGGCTCCAGGGCCTTCACCAAGCACCCCGCCGACATCCCCGACTACTATAAGCAGTCCTTCCCCGAGGGCTTCAAGTGG
GAGCGCGTGATGAACTTCGAGGACGGCGGCGCCGTGACCGTGACCCAGGACACCTCCCTGGAGGACGGCACCCTGATCTACAAGGT
GAAGCTCCGCGGCACCAACTTCCCTCCTGACGGCCCCGTAATGCAGAAGAAGACAATGGGCTGGGAAGCGTCCACCGAGCGGTTGT
ACCCCGAGGACGGCGTGCTGAAGGGCGACATTAAGATGGCCCTGCGCCTGAAGGACGGCGGCCGCTACCTGGCGGACTTCAAGACC
ACCTACAAGGCCAAGAAGCCCGTGCAGATGCCCGGCGCCTACAACGTCGACCGCAAGTTGGACATCACCTCCCACAACGAGGACTA
CACCGTGGTGGAACAGTACGAACGCTCCGAGGGCCGCCACTCCACCGGCGGCATGGACGAGCTGTACAAGCCCTGGAGATTTTCAT
CACCTCTTCTGGAGAAGAATACTCAGGTGGCGTCATTGGGAAGATCCATGCCACAGAAACTCTGCGCGGGACTGGACTGCCCCTGG
AAGTTCTGCGATGAAAAGGTGTCTGTGGATGAAAGTGTGATGTCAACACACAGCACAGCCAGACTGAGTTTTGTGACTCCCCGCCA
CCACAGGGCAGCGGTGTGTCTCTGCAAAGAGGGAAGGTGCCCACCTGTCCACCATGGCTGTGAAGATGATCCGTGCCCTGAGGGAT
CCGAATGTGTGTCTGATCCCTGGGAGGAGAAACACACCTGTGTCTGTCCCAGCGGCAGGTTTGGTCAGTGCCCAGGGAGTTCATCT
ATGACACTGACTGGAAACAGCTACGTGAAATACCGTCTGACGGAAAATGAAAACAAATTAGAGATGAAACTGACCATGAGGCTCAG
AACATATTCCACGCATGCGGTTGTCATGTATGCTCGAGGAACTGACTATAGCATCTTGGAGATTCATCATGGAAGGCTGCAGTACA
AGTTTGACTGTGGAAGTGGCCCTGGAATTGTCTCTGTTCAGAGCATTCAGGTCAATGATGGGCAGTGGCACGCAGTGGCCCTGGAA
GTGAATGGAAACTATGCTCGCTTGGTTCTAGACCAAGTTCATACTGCATCGGGCACAGCCCCAGGGACTCTGAAAACCCTGAACCT
GGATAACTATGTGTTTTTTGGTGGCCACATCCGTCAGCAGGGAACAAGGCATGGAAGAAGTCCTCAAGTTGGTAATGGTTTCAGGG
GTTGTATGGACTCCATTTATTTGAATGGGCAGGAGCTCCCTTTAAACAGCAAACCCAGAAGCTATGCACACATCGAAGAGTCGGTG
GATGTATCTCCAGGCTGCTTCCTGACGGCCACGGAAGACTGCGCCAGCAACCCTTGCCAGAATGGAGGCGTTTGCAATCCGTCACC
TGCTGGAGGTTATTACTGCAAATGCAGTGCCTTGTACATAGGGACCCACTGTGAGATAAGCGTCAATCCGTGTTCCTCCAAGCCAT
GCCTCTATGGGGGCACGTGTGTTGTCGACAACGGAGGCTTTGTTTGCCAGTGTAGAGGATTATATACTGGTCAGAGGTGTCAGCTT
AGTCCATACTGCAAAGATGAACCCTGTAAGAATGGCGGAACATGCTTTGACAGTTTGGATGGCGCCGTTTGTCAGTGTGATTCGGG
TTTTAGGGGAGAAAGGTGTCAGAGTGATATCGACGAGTGCTCTGGAAACCCTTGCCTGCACGGGGCCCTCTGTGAGAACACGCACG
GCTCCTATCACTGCAACTGCAGCCACGAGTACAGGGGACGTCACTGCGAGGATGCTGCGCCCAACCAGTATGTGTCCACGCCGTGG
AACATTGGGTTGGCGGAAGGAATTGGAATCGTTGTGTTTGTTGCAGGGATATTTTTACTGGTGGTGGTGTTTGTTCTCTTGGTACC
GGTAGTATCAGCTAAGCCTGTACCGAGGAGATCTGCCACCATGCATCACCATCACCATCACGATTACCCATACGATGTTCCAGATT
ACGCTGCGATCGTGTGCCGTAAGATGATTAGTCGGAAAAAGAAGCATCAGGCTGAACCTAAAGACAAGCACCTGGGACCCGCTACG
GCTTTCTTGCAAAGACCGTATTTTGATTCCAAGCTAAATAAGAACATTTACTCAGACATACCACCCCAGGTGCCTGTCCGGCCTAT
TTCCTACACCCCGAGTATTCCAAGTGACTCAAGAAACAATCTGGACCGAAATAGTTTTGAAGGATCTGCTATCCCAGAGCATCCCG
AATTCAGCACTTTTAACCCCGAGTCTGTGCACGGGCACCGAAAAGCAGTGGCGGTCTGCAGCGTGGCGCCAAACCTGTCTCCCCCA
CCCCCTTCAAACTCCCCTTCTGACAGCGACTCCATCCAGAAGCCTAGCTGGGACTTTGACTATGACACAAAAGTGGTGGATCTTGA
TCCCTGTCTTTCCAAGAAGCCTCTAGAGGAAAAGCCTTCCCAGCCATACAGTGCCCGGGAAAGCCTGTCTGAAGTGCAGTCTCTGA
GCTCCTTCCAGTCCGAATCGTGCGATGACAATGGGTATCACTGGGATACATCAGATTGGATGCCAAGCGTTCCTCTGCCGGACATA
CAAGAGTTCCCCAACTATGAGGTGATTGATGAGCAGACACCCCTGTACTCAGCAGATCCAAACGCCATCGATACGGACTATTACCC
TGGAGGCTACGACATCGAAAGTGATTTTCCTCCACCCCCAGAAGACTTCCCCGCAGCTGATGAGCTACCACCGTTACCGCCCGAAT
TCAGCAATCAGTTTGAATCCATCCACCCTCCTAGAGACATGCCTGCCGCGGGTAGCTTGGGTTCTTCATCAAGAAACCGGCAGAGG
TTCAACTTGAATCAGTATTTGCCCAATTTTTATCCCCTCGATATGTCTGAACCTCAAACAAAAGGCACTGGTGAGAATAGTACTTG
TAGAGAACCCCATGCCCCTTACCCGCCAGGGTATCAAAGACACTTCGAGGCGCCCGCTGTCGAGAGCATGCCCATGTCTGTGTACG
CCTCCACCGCCTCCTGCTCTGACGTGTCAGCCTGCTGCGAAGTGGAGTCCGAGGTCATGATGAGTGACTATGAGAGCGGGGACGAC
GGCCACTTCGAAGAGGTGACGATCCCGCCCCTGGATTCCCAGCAGCACACGGAAGTCTGAACGCGTGAGCGGCCGCACTCGAGGTT
TAAACGGCCGGCCGCGGTCATAGCTGTTTCCTGAACAGATCCCGGGTGGCATCCCTGTGACCCCTCCCCAGTGCCTCTCCTGGCCC
TGGAAGTTGCCACTCCAGTGCCCACCAGCCTTGTCCTAATAAAATTAAGTTGCATCATTTTGTCTGACTAGGTGTCCTTCTATAAT
ATTATGGGGTGGAGGGGGGTGGTATGGAGCAAGGGGCAAGTTGGGAAGACAACCTGTAGGGCCTGCGGGGTCTATTGGGAACCAAG
CTGGAGTGCAGTGGCACAATCTTGGCTCACTGCAATCTCCGCCTCCTGGGTTCAAGCGATTCTCCTGCCTCAGCCTCCCGAGTTGT
TGGGATTCCAGGCATGCATGACCAGGCTCAGCTAATTTTTGTTTTTTTGGTAGAGACGGGGTTTCACCATATTGGCCAGGCTGGTC
TCCAACTCCTAATCTCAGGTGATCTACCCACCTTGGCCTCCCAAATTGCTGGGATTACAGGCGTGAACCACTGCTCCCTTCCCTGT
CCTTCTGATTTTAAAATAACTATACCAGCAGGAGGACGTCCAGACACAGCATAGGCTACCTGGCCATGCCCAACCGGTGGGACATT
TGAGTTGCTTGCTTGGCACTGTCCTCTCATGCATTGGGTCCACTCAGTAGATGCCTGTTGAATTGGGTACGCGGCCAGCTTGGCTG
TGGAATGTGTGTCAGTTAGGGTGTGGAAAGTCCCCAGGCTCCCCAGCAGGCAGAAGTATGCAAAGCATGCATCTCAATTAGTCAGC
AACCAGGTGTGGAAAGTCCCCAGGCTCCCCAGCAGGCAGAAGTATGCAAAGCATGCATCTCAATTAGTCAGCAACCATAGTCCCGC
CCCTAACTCCGCCCATCCCGCCCCTAACTCCGCCCAGTTCCGCCCATTCTCCGCCCCATGGCTGACTAATTTTTTTTATTTATGCA
GAGGCCGAGGCCGCCTCGGCCTCTGAGCTATTCCAGAAGTAGTGAGGAGGCTTTTTTGGAGGCCTAGGCTTTTGCAAAAAGCTCCC
GGGAGCTTGTATATCCATTTTCGGATCTGATCAAGAGACAGGATGAGGATCGTTTCGCATGATTGAACAAGATGGATTGCACGCAG
GTTCTCCGGCCGCTTGGGTGGAGAGGCTATTCGGCTATGACTGGGCACAACAGACAATCGGCTGCTCTGATGCCGCCGTGTTCCGG
CTGTCAGCGCAGGGGCGCCCGGTTCTTTTTGTCAAGACCGACCTGTCCGGTGCCCTGAATGAACTGCAGGACGAGGCAGCGCGGCT
ATCGTGGCTGGCCACGACGGGCGTTCCTTGCGCAGCTGTGCTCGACGTTGTCACTGAAGCGGGAAGGGACTGGCTGCTATTGGGCG
AAGTGCCGGGGCAGGATCTCCTGTCATCTCACCTTGCTCCTGCCGAGAAAGTATCCATCATGGCTGATGCAATGCGGCGGCTGCAT
ACGCTTGATCCGGCTACCTGCCCATTCGACCACCAAGCGAAACATCGCATCGAGCGAGCACGTACTCGGATGGAAGCCGGTCTTGT
CGATCAGGATGATCTGGACGAAGAGCATCAGGGGCTCGCGCCAGCCGAACTGTTCGCCAGGCTCAAGGCGCGCATGCCCGACGGCG
AGGATCTCGTCGTGACCCATGGCGATGCCTGCTTGCCGAATATCATGGTGGAAAATGGCCGCTTTTCTGGATTCATCGACTGTGGC
CGGCTGGGTGTGGCCGACCGCTATCAGGACATAGCGTTGGCTACCCGTGATATTGCTGAAGAGCTTGGCGGCGAATGGGCTGACCG
CTTCCTCGTGCTTTACGGTATCGCCGCTCCCGATTCGCAGCGCATCGCCTTCTATCGCCTTCTTGACGAGTTCTTCTGAGCGGGAC
TCTGGGGTTCGAAATGACCGACCAAGCGACGCCCAACCTGCCATCACGAGATTTCGATTCCACCGCCGCCTTCTATGAAAGGTTGG
GCTTCGGAATCGTTTTCCGGGACGCCGGCTGGATGATCCTCCAGCGCGGGGATCTCATGCTGGAGTTCTTCGCCCACCCCAACTTG
TTTATTGCAGCTTATAATGGTTACAAATAAAGCAATAGCATCACAAATTTCACAAATAAAGCATTTTTTTCACTGCATTCTAGTTG
TGGTTTGTCCAAACTCATCAATGTATCTTATCATGTCTGTATACCGTCGACCTCTAGCTAGAGCTTGGCGTAATCATGGTCATAGC
TGTTTCCTGTGTGAAATTGTTATCCGCTCACAATTCCACACAACATACGAGCCGGAAGCATAAAGTGTAAAGCCTGGGGTGCCTAA
TGAGTGAGCTAACTCACATTAATTGCGTTGCGCTCACTGCCCGCTTTCCAGTCGGGAAACCTGTCGTGCCAGCTGCATTAATGAAT
CGGCCAACGCGCGGGGAGAGGCGGTTTGCGTATTGGGCGCTCTTCCGCTTCCTCGCTCACTGACTCGCTGCGCTCGGTCGTTCGGC
TGCGGCGAGCGGTATCAGCTCACTCAAAGGCGGTAATACGGTTATCCACAGAATCAGGGGATAACGCAGGAAAGAACATGTGAGCA
AAAGGCCAGCAAAAGGCCAGGAACCGTAAAAAGGCCGCGTTGCTGGCGTTTTTCCATAGGCTCCGCCCCCCTGACGAGCATCACAA
AAATCGACGCTCAAGTCAGAGGTGGCGAAACCCGACAGGACTATAAAGATACCAGGCGTTTCCCCCTGGAAGCTCCCTCGTGCGCT
CTCCTGTTCCGACCCTGCCGCTTACCGGATACCTGTCCGCCTTTCTCCCTTCGGGAAGCGTGGCGCTTTCTCATAGCTCACGCTGT
AGGTATCTCAGTTCGGTGTAGGTCGTTCGCTCCAAGCTGGGCTGTGTGCACGAACCCCCCGTTCAGCCCGACCGCTGCGCCTTATC
CGGTAACTATCGTCTTGAGTCCAACCCGGTAAGACACGACTTATCGCCACTGGCAGCAGCCACTGGTAACAGGATTAGCAGAGCGA
GGTATGTAGGCGGTGCTACAGAGTTCTTGAAGTGGTGGCCTAACTACGGCTACACTAGAAGAACAGTATTTGGTATCTGCGCTCTG
CTGAAGCCAGTTACCTTCGGAAAAAGAGTTGGTAGCTCTTGATCCGGCAAACAAACCACCGCTGGTAGCGGTGGTTTTTTTGTTTG
CAAGCAGCAGATTACGCG

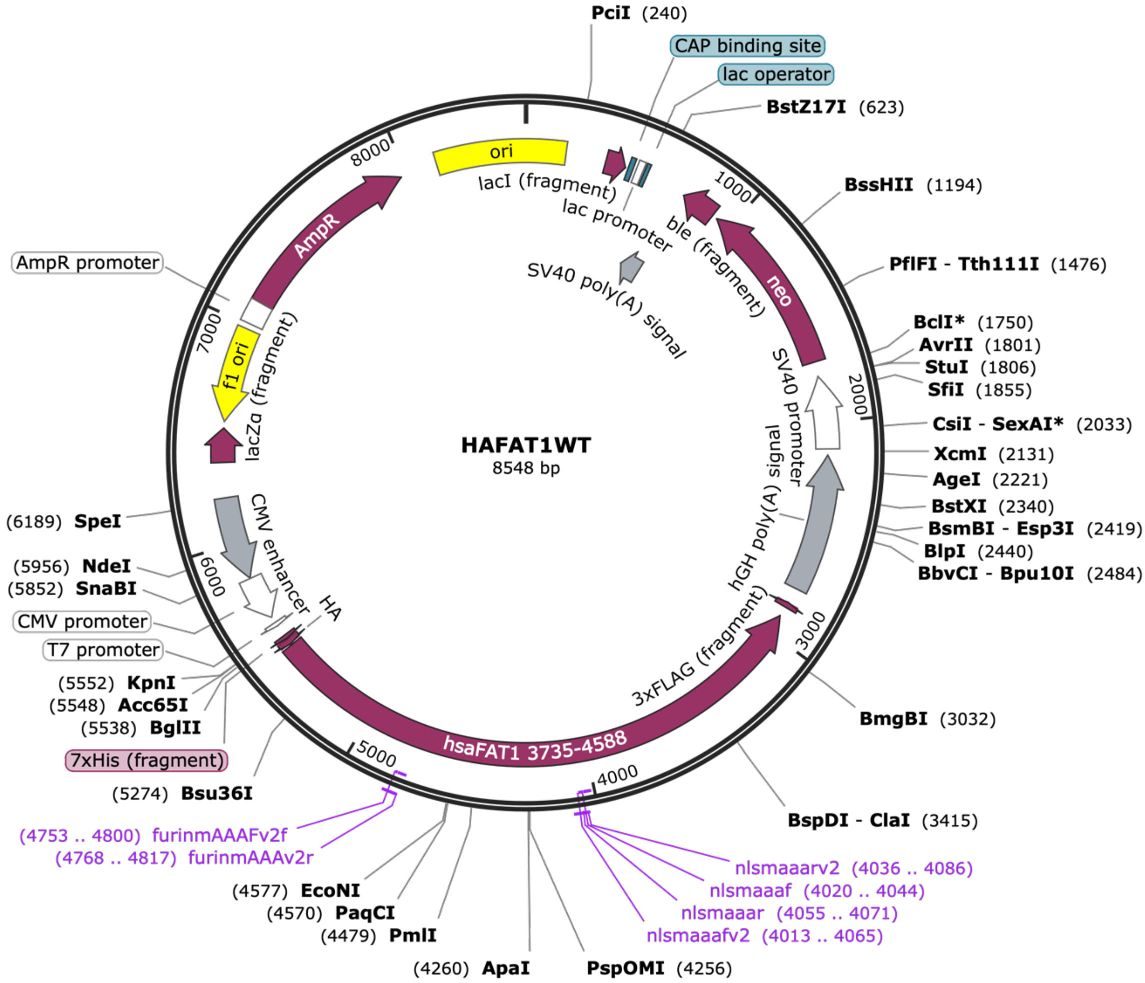

> HA-FAT1$^{3735-4588}$WT, 8548bps

```
cacgcttcccgaagggagaaaggcggacaggtatccggtaagcggcagggtcggaacaggagagcgcacgagggagcttccagggg
gaaacgcctggtatctttatagtcctgtcgggtttcgccacctctgacttgagcgtcgatttttgtgatgctcgtcaggggggcgg
agcctatggaaaaacgccagcaacgcggcctttttacggttcctggccttttgctggccttttgctcacatgttctttcctgcgtt
atcccctgattctgtggataaccgtattaccgcctttgagtgagctgataccgctcgccgcagccgaacgaccgagcgcagcgagt
cagtgagcgaggaagcggaagagcgcccaatacgcaaaccgcctctccccgcgcgttggccgattcattaatgcagctggcacgac
aggtttcccgactggaaagcgggcagtgagcgcaacgcaattaatgtgagttagctcactcattaggcaccccaggctttacactt
tatgcttccggctcgtatgttgtgtggaattgtgagcggataacaatttcacacaggaaacagctatgaccatgattacgccaagc
tctagctagaggtcgacggtatacagacatgataagatacattgatgagtttggacaaaccacaactagaatgcagtgaaaaaaat
gctttatttgtgaaatttgtgatgctattgctttatttgtaaccattataagctgcaataaacaagttggggtgggcgaagaactc
cagcatgagatccccgcgctggaggatcatccagccggcgtcccggaaaacgattccgaagcccaacctttcatagaaggcggcgg
tggaatcgaaatctcgtgatggcaggttgggcgtcgcttggtcggtcatttcgaaccccagagtcccgctcagaagaactcgtcaa
gaaggcgatagaaggcgatgcgctgcgaatcgggagcggcgataccgtaaagcacgaggaagcggtcagcccattcgccgccaagc
tcttcagcaatatcacgggtagccaacgctatgtcctgatagcggtcggccacacccagccggccacagtcgatgaatccagaaaa
gcggccattttccaccatgatattcggcaagcaggcatcgccatgggtcacgacgagatcctcgccgtcgggcatgcgcgccttga
gcctggcgaacagttcggctggcgcgagcccctgatgctcttcgtccagatcatcctgatcgacaagaccggcttccatccgagta
cgtgctcgctcgatgcgatgtttcgcttggtggtcgaatgggcaggtagccggatcaagcgtatgcagccgccgcattgcatcagc
catgatggatactttctcggcaggagcaaggtgagatgacaggagatcctgccccggcacttcgcccaatagcagccagtcccttc
ccgcttcagtgacaacgtcgagcacagctgcgcaaggaacgcccgtcgtggccagccacgatagccgcgctgcctcgtcctgcagt
tcattcagggcaccggacaggtcggtcttgacaaaaagaaccgggcgcccctgcgctgacagccggaacacggcggcatcagagca
gccgattgtctgttgtgcccagtcatagccgaatagcctctccacccaagcggccggagaacctgcgtgcaatccatcttgttcaa
tcatgcgaaacgatcctcatcctgtctcttgatcagatccgaaaatggatatacaagctcccgggagctttttgcaaaagcctagg
cctccaaaaaagcctcctcactacttctggaatagctcagaggccgaggcggcctcggcctctgcataaataaaaaaaattagtca
gccatggggcggagaatgggcggaactgggcggagttaggggcgggatgggcggagttaggggcgggactatggttgctgactaat
tgagatgcatgctttgcatacttctgcctgctggggagcctggggactttccacacctggttgctgactaattgagatgcatgctt
tgcatacttctgcctgctggggagcctggggactttccacaccctaactgacacacattccacagccaagctggccgcgtacccaa
ttcaacaggcatctactgagtggacccaacgcatgagaggacagtgccaagcaagcaactcaaatgtcccaccggttgggcatggc
caggtagcctatgctgtgtctggacgtcctcctgctggtatagttattttaaaatcagaaggacagggaagggagcagtggttcac
gcctgtaatcccagcaatttgggaggccaaggtgggtagatcacctgagattaggagttggagaccagcctggccaatatggtgaa
accccgtctctaccaaaaaaacaaaaattagctgagcctggtcatgcatgcctggaatcccaacaactcgggaggctgaggcagga
gaatcgcttgaacccaggaggcggagattgcagtgagccaagattgtgccactgcactccagcttggttcccaatagaccccgcag
gccctacaggttgtcttcccaacttgccccttgctccataccaccccccctccaccccataatattatagaaggacacctagtcaga
```

caaaatgatgcaacttaattttattaggacaaggctggtgggcactggagtggcaacttccagggccaggagaggcactggggagg
ggtcacagggatgccacccgggatctgttcaggaaacagctatgaccgcggccggccgtttaaacctcgagtgccggccggccgtt
taaaccttatcgtcgtcatccttgtaatccaggatatcatttgctgccagatcctcttctgagatgagtttctggacttccgtgtg
ctgctgggaatccaggggcgggatcgtcacctcttcgaagtggccgtcgtccccgctctcatagtcactcatcatgacctcggact
ccacttcgcagcaggctgacacgtcagagcaggaggcggtggaggcgtacacagacatgggcatgctctcgacagcgggcgcctcg
aagtgtctttgataccctggcgggtaaggggcatggggttctctacaagtactattctcaccagtgccttttgtttgaggttcaga
catatcgaggggataaaaattgggcaaatactgattcaagttgaacctctgccggtttcttgatgaagaacccaagctacccgcgg
caggcatgtctctaggagggtggatggattcaaactgattgctgaattcgggcggtaacggtggtagctcatcagctgcggggaag
tcttctgggggtggaggaaaatcactttcgatgtcgtagcctccagggtaatagtccgtatcgatggcgtttggatctgctgagta
caggggtgtctgctcatcaatcacctcatagttggggaactcttgtatgtccggcagaggaacgcttggcatccaatctgatgtat
cccagtgatacccattgtcatcgcacgattcggactggaaggagctcagagactgcacttcagacaggctttcccgggcactgtat
ggctgggaaggcttttcctctagaggcttcttggaaagacagggatcaagatccaccacttttgtgtcatagtcaaagtcccagct
aggcttctggatggagtcgctgtcagaaggggagtttgaaggggggtgggggaggcaggtttggcgccacgctgcagaccgccactg
cttttcggtgcccgtgcacagactcggggttaaaagtgctgaattcgggatgctctgggatagcagatccttcgaaggaatttcgg
tccagattgtttcttgagtcacttggaatactcggggtgtaggaaataggccggacaggcacctggggtggtatgtctgagtaaat
gttcttatttagcttggaatcaaaatacggtctttgcaagaaagccgtagcgggtcccaggtgcttgtctttaggttcagcctgat
gcttctttttccgactaatcatcttacggcagagaacaaacaccaccaccagtaaaaatatccctgcaacaaacacaacgattcca
attccttccgccaacccaatgttccacggcgtggacacatactggttgggcgcagcatcctcgcagtgacgtcccctgtactcgtg
gctgcagttgcagtgataggagccgtgcgtgttctcacagagggccccgtgcaggcaagggtttccagagcactcgtcgatatcac
tctgacacctttctcccctaaaacccgaatcacactgacaaacggcgccatccaaactgtcaaagcatgttccgccattcttacag
ggttcatctttgcagtatggactaagctgacacctctgaccagtatataatcctctacactggcaaacaaagcctccgttgtcgac
aacacacgtgcccccatagaggcatggcttggaggaacacggattgacgcttatctcacagtgggtccctatgtacaaggcactgc
atttgcagtaataacctccagcaggtgacggattgcaaacgcctccattctggcaagggttgctggcgcagtcttccgtggccgtc
aggaagcagcctggagatacatccaccgactcttcgatgtgtgcatagcttctgggtttgctgtttaaagggagctcctgcccatt
caaataaatggagtccatacaacccctgaaaccattaccaacttgaggacttcttccatgccttgttccctgctgacggatgtggc
caccaaaaaacacatagttatccaggttcagggttttcagagtccctggggctgtgcccgatgcagtatgaacttggtctagaacc
aagcgagcatagtttccattcacttccagggccactgcgtgccactgcccatcattgacctgaatgctctgaacagagacaattcc
agggccacttccacagtcaaacttgtactgcagccttccatgatgaatctccaagatgctatagtcagttcctcgagcatacatga
caaccgcatgcgtggaatatgttctgagcctcatggtcagtttcatctctaatttgttttcattttccgtcagacggtatttcacg
tagctgtttccagtcagtgtcatagatgaactccctgggcactgaccaaacctgccgctgggacagacacaggtgtgtttctcctc
ccagggatcagacacacattcggatccctcagggcacggatcatcttcacagccatggtggacaggtgggcaccttccctctttgc
agagacacaccgctgccctgtggtggcggggagtcacaaaactcagtctggctgtgctgtgtgttgacatcacactttcatccaca
gacaccttttcatcgcagaacttccaggggcagtccagtcccgcgcagagtttctgtatcgcagcgtaatctggaacatcgtatgg
gtaatcgtgatggtgatggtgatgcatggtggcagatctcctcggtaccggatccagtcgacgaattcccggccgccctatagtga
gtcgtattacaaaattctgacggttcactaaacgagctctgcttatatagacctcccaccgtacacgcctaccgcccatttgcgtc
aacggggcggggttattacgacattttggaaagtcccgttgattttggtgccaaaacaaactcccattgacgtcaatggggtggag
acttggaaatccccgtgagtcaaaccgctatccacgcccattggtgtactgccaaaaccgcatcaccatggtaatagcgatgacta
atacgtagatgtactgccaagtaggaaagtcccgtaaggtcatgtactgggcataatgccaggcgggccatttaccgtcattgacg
tcaataggggggcggacttggcatatgatacacttgatgtactgccaagtgggcagtttaccgtaaatactccacccattgacgtca
atggaaagtccctattggcgttactatgggaacatacgtcattattgacgtcaatgggcgggggtcgttgggcggtcagccaggcg
ggccatttaccgtaagttatgtaacgcggaactccatatatgggctatgaactaatgaccccgtaattgattactattaataacta
gtcaataatcaatgtcaacatggcggtcatattggacatgagccaatataaatgtacatattatgatatagatacaacgtatgcaa
tggccaatagccaatattgatttatgctatataaccaatgactaatatggctaattgccaatattgattcaatgtatagatcagct
tggcactggccgtcgttttacaacgtcgtgactgggaaaaccctggcgttacccaacttaatcgccttgcagcacatccccctttc
gccagctggcgtaatagcgaagaggcccgcaccgatcgcccttcccaacagttgcgcagcctgaatggcgaatggaaattgtaagc
gttaatattttgttaaaattcgcgttaaatttttgttaaatcagctcattttttaaccaataggccgaaatcggcaaaatccctta
taaatcaaaagaatagaccgagatagggttgagtgttgttccagtttggaacaagagtccactattaaagaacgtggactccaacg
tcaaagggcgaaaaaccgtctatcagggcgatggcccactacgtgaaccatcaccctaatcaagttttttggggtcgaggtgccgt
aaagcactaaatcggaaccctaaagggagcccccgatttagagcttgacggggaaagccggcgaacgtggcgagaaaggaagggaa
gaaagcgaaaggagcgggcgctagggcgctggcaagtgtagcggtcacgctgcgcgtaaccaccacacccgccgcgcttaatgcgc
cgctacagggcgcgtcaggtggcacttttcggggaaatgtgcgcggaacccctatttgtttatttttctaaatacattcaaatatg
tatccgctcatgagacaataaccctgataaatgcttcaataatattgaaaaaggaagagtatgagtattcaacatttccgtgtcgc
ccttattcccttttttgcggcattttgccttcctgtttttgctcacccagaaacgctggtgaaagtaaaagatgctgaagatcagt
tgggtgcacgagtgggttacatcgaactggatctcaacagcggtaagatccttgagagttttcgccccgaagaacgttttccaatg
atgagcacttttaaagttctgctatgtggcgcggtattatcccgtattgacgccgggcaagagcaactcggtcgccgcatacacta
ttctcagaatgacttggttgagtactcaccagtcacagaaaagcatcttacggatggcatgacagtaagagaattatgcagtgctg
ccataaccatgagtgataacactgcggccaacttacttctgacaacgatcggaggaccgaaggagctaaccgcttttttgcacaac
atgggggatcatgtaactcgccttgatcgttgggaaccggagctgaatgaagccataccaaacgacgagcgtgacaccacgatgcc
tgtagcaatggcaacaacgttgcgcaaactattaactggcgaactacttactctagcttcccggcaacaattaatagactggatgg
aggcggataaagttgcaggaccacttctgcgctcggcccttccggctggctggtttattgctgataaatctggagccggtgagcgt
gggtctcgcggtatcattgcagcactggggccagatggtaagccctcccgtatcgtagttatctacacgacggggagtcaggcaac
tatggatgaacgaaatagacagatcgctgagataggtgcctcactgattaagcattggtaactgtcagaccaagtttactcatata
tactttagattgatttaaaacttcatttttaatttaaaaggatctaggtgaagatcctttttgataatctcatgaccaaaatccct
taacgtgagttttcgttccactgagcgtcagaccccgtagaaaagatcaaaggatcttcttgagatcctttttttctgcgcgtaat
ctgctgcttgcaaacaaaaaaaccaccgctaccagcggtggtttgtttgccggatcaagagctaccaactctttttccgaaggtaa
ctggcttcagcagagcgcagataccaaatactgttcttctagtgtagccgtagttaggccaccacttcaagaactctgtagcaccg
cctacatacctcgctctgctaatcctgttaccagtggctgctgccagtggcgataagtcgtgtcttaccgggttggactcaagacg
atagttaccggataaggcgcagcggtcgggctgaacggggggttcgtgcacacagcccagcttggagcgaacgacctacaccgaac
tgagatacctacagcgtgagctatgagaaagcgc

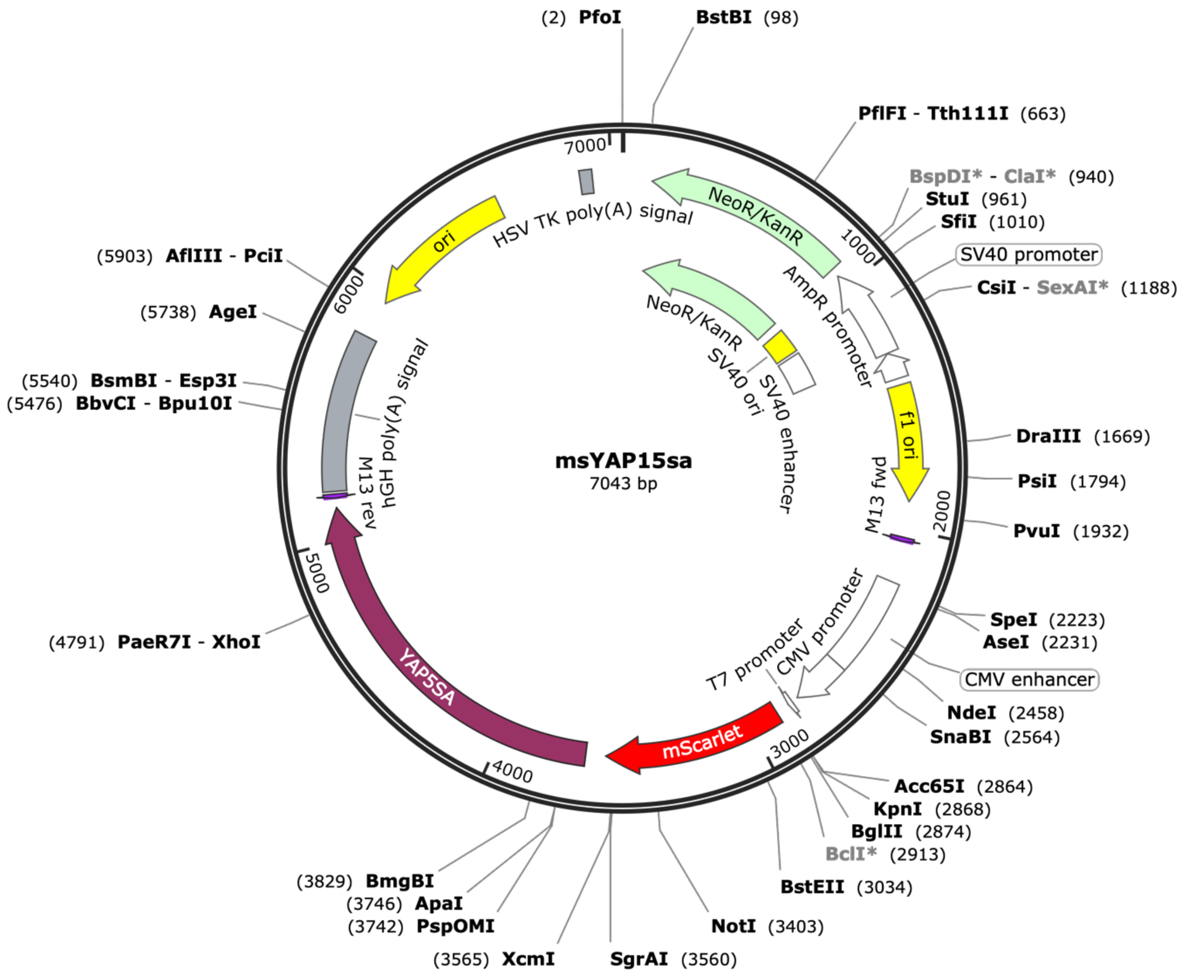

>mScarletYAP1^{5SA} 7043bps

GTCCCGGAAAACGATTCCGAAGCCCAACCTTTCATAGAAGGCGGCGGTGGAATCGAAATCTCGTGATGGCAGGTTGGGCGTCGCTT
GGTCGGTCATTTCGAACCCCAGAGTCCCGCTCAGAAGAACTCGTCAAGAAGGCGATAGAAGGCGATGCGCTGCGAATCGGGAGCGG
CGATACCGTAAAGCACGAGGAAGCGGTCAGCCCATTCGCCGCCAAGCTCTTCAGCAATATCACGGGTAGCCAACGCTATGTCCTGA
TAGCGATCCGCCACACCCAGCCGGCCACAGTCGATGAATCCAGAAAAGCGGCCATTTTCCACCATGATATTCGGCAAGCAGGCATC
GCCATGGGTCACGACGAGATCCTCGCCGTCGGGCATGCTCGCCTTGAGCCTGGCGAACAGTTCGGCTGGCGCGAGCCCCTGATGCT
CTTCGTCCAGATCATCCTGATCGACAAGACCGGCTTCCATCCGAGTACGTGCTCGCTCGATGCGATGTTTCGCTTGGTGGTCGAAT
GGGCAGGTAGCCGGATCAAGCGTATGCAGCCGCCGCATTGCATCAGCCATGATGGATACTTTCTCGGCAGGAGCAAGGTGAGATGA
CAGGAGATCCTGCCCCGGCACTTCGCCCAATAGCAGCCAGTCCCTTCCCGCTTCAGTGACAACGTCGAGCACAGCTGCGCAAGGAA
CGCCCGTCGTGGCCAGCCACGATAGCCGCGCTGCCTCGTCTTGCAGTTCATTCAGGGCACCGGACAGGTCGGTCTTGACAAAAAGA
ACCGGGCGCCCCTGCGCTGACAGCCGGAACACGGCGGCATCAGAGCAGCCGATTGTCTGTTGTGCCCAGTCATAGCCGAATAGCCT
CTCCACCCAAGCGGCCGGAGAACCTGCGTGCAATCCATCTTGTTCAATCATGCGAAACGATCCTCATCCTGTCTCTTGATCGATCT
TTGCAAAAGCCTAGGCCTCCAAAAAAGCCTCCTCACTACTTCTGGAATAGCTCAGAGGCCGAGGCGGCCTCGGCCTCTGCATAAAT
AAAAAAAATTAGTCAGCCATGGGGCGGAGAATGGGCGGAACTGGGCGGAGTTAGGGGCGGGATGGGCGGAGTTAGGGGCGGGACTA
TGGTTGCTGACTAATTGAGATGCATGCTTTGCATACTTCTGCCTGCTGGGGAGCCTGGGGACTTTCCACACCTGGTTGCTGACTAA
TTGAGATGCATGCTTTGCATACTTCTGCCTGCTGGGGAGCCTGGGGACTTTCCACACCCTAACTGACACACATTCCACAGCTGGTT
CTTTCCGCCTCAGGACTCTTCCTTTTTCAATATTATTGAAGCATTTATCAGGGTTATTGTCTCATGAGCGGATACATATTTGAATG
TATTTAGAAAAATAAACAAATAGGGGTTCCGCGCACATTTCCCCGAAAAGTGCCACCTGACGCGCCCTGTAGCGGCGCATTAAGCG
CGGCGGGTGTGGTGGTTACGCGCAGCGTGACCGCTACACTTGCCAGCGCCCTAGCGCCCGCTCCTTTCGCTTTCTTCCCTTCCTTT
CTCGCCACGTTCGCCGGCTTTCCCCGTCAAGCTCTAAATCGGGGGCTCCCTTTAGGGTTCCGATTTAGTGCTTTACGGCACCTCGA
CCCCAAAAAACTTGATTAGGGTGATGGTTCACGTAGTGGGCCATCGCCCTGATAGACGGTTTTTCGCCCTTTGACGTTGGAGTCCA
CGTTCTTTAATAGTGGACTCTTGTTCCAAACTGGAACAACACTCAACCCTATCTCGGTCTATTCTTTTGATTTATAAGGGATTTTG
CCGATTTCGGCCTATTGGTTAAAAAATGAGCTGATTTAACAAAAATTTAACGCGAATTTTAACAAAATATTAACGCTTACAATTTC
CATTCGCCATTCAGGCTGCGCAACTGTTGGGAAGGGCGATCGGTGCGGGCCTCTTCGCTATTACGCCAGCTGGCGAAAGGGGGATG
TGCTGCAAGGCGATTAAGTTGGGTAACGCCAGGGTTTTCCCAGTCACGACGTTGTAAAACGACGGCCAGTGCCAAGCTGATCTATA
CATTGAATCAATATTGGCAATTAGCCATATTAGTCATTGGTTATATAGCATAAATCAATATTGGCTATTGGCCATTGCATACGTTG
TATCTATATCATAATATGTACATTTATATTGGCTCATGTCCAATATGACCGCCATGTTGACATTGATTATTGACTAGTTATTAATA
GTAATCAATTACGGGGTCATTAGTTCATAGCCCATATATGGAGTTCCGCGTTACATAACTTACGGTAAATGGCCCGCCTGGCTGAC
CGCCCAACGACCCCCGCCCATTGACGTCAATAATGACGTATGTTCCCATAGTAACGCCAATAGGGACTTTCCATTGACGTCAATGG
GTGGAGTATTTACGGTAAACTGCCCACTTGGCAGTACATCAAGTGTATCATATGCCAAGTCCGCCCCCTATTGACGTCAATGACGG
TAAATGGCCCGCCTGGCATTATGCCCAGTACATGACCTTACGGGACTTTCCTACTTGGCAGTACATCTACGTATTAGTCATCGCTA
TTACCATGGTGATGCGGTTTTGGCAGTACACCAATGGGCGTGGATAGCGGTTTGACTCACGGGGATTTCCAAGTCTCCACCCCATT

GACGTCAATGGGAGTTTGTTTTGGCACCAAAATCAACGGGACTTTCCAAAATGTCGTAATAACCCCGCCCCGTTGACGCAAATGGG
CGGTAGGCGTGTACGGTGGGAGGTCTATATAAGCAGAGCTCGTTTAGTGAACCGTCAGAATTTTGTAATACGACTCACTATAGGGC
GGCCGGGAATTCGTCGACTGGATCCGGTACCGAGGAGATCTGCCGCCGCGATATGGTGAGCAAGGGCGAGGCAGTGATCAAGGAGT
TCATGCGGTTCAAGGTGCACATGGAGGGCTCCATGAACGGCCACGAGTTCGAGATCGAGGGCGAGGGCGAGGGCCGCCCCTACGAG
GGCACCCAGACCGCCAAGCTGAAGGTGACCAAGGGTGGCCCCCTGCCCTTCTCCTGGGACATCCTGTCCCCTCAGTTCATGTACGG
CTCCAGGGCCTTCACCAAGCACCCCGCCGACATCCCCGACTACTATAAGCAGTCCTTCCCCGAGGGCTTCAAGTGGGAGCGCGTGA
TGAACTTCGAGGACGGCGGCGCCGTGACCGTGACCCAGGACACCTCCCTGGAGGACGGCACCCTGATCTACAAGGTGAAGCTCCGC
GGCACCAACTTCCCTCCTGACGGCCCCGTAATGCAGAAGAAGACAATGGGCTGGGAAGCGTCCACCGAGCGGTTGTACCCCGAGGA
CGGCGTGCTGAAGGGCGACATTAAGATGGCCCTGCGCCTGAAGGACGGCGGCCGCTACCTGGCGGACTTCAAGACCACCTACAAGG
CCAAGAAGCCCGTGCAGATGCCCGGCGCCTACAACGTCGACCGCAAGTTGGACATCACCTCCCACAACGAGGACTACACCGTGGTG
GAACAGTACGAACGCTCCGAGGGCCGCCACTCCACCGGCGGCATGGACGAGCTGTACAAGCCCTGGAGATTTTCATCACCTCTTCT
GGAGAAGAATACTCAGGTGGCGTCATTGGGAAGATCCATGCCAGTACCTAAGGATCCCGGGCAGCAGCCGCCGCCTCAACCGGCCC
CCCAGGGCCAAGGGCAGCCGCCTTCGCAGCCCCCGCAGGGGCAGGGCCCGCCGTCCGGACCCGGGCAACCGGCACCCGCGGCGACC
CAGGCGGCGCCGCAGGCACCCCCCGCCGGGCATCAGATCGTGCACGTCCGCGGGGACGCGGAGACCGACCTGGAGGCGCTCTTCAA
CGCCGTCATGAACCCCAAGACGGCCAACGTGCCCCAGACCGTGCCCATGAGGCTCCGGAAGCTGCCCGACTCCTTCTTCAAGCCGC
CGGAGCCCAAATCCCACTCCCGACAGGCCGCTACTGATGCAGGCACTGCAGGAGCCCTGACTCCACAGCATGTTCGAGCTCATGCC
GCTCCAGCTGCTCTGCAGTTGGGAGCTGTTTCTCCTGGGACACTGACCCCCACTGGAGTAGTCTCTGGCCCAGCAGCTACACCCAC
AGCTCAGCATCTTCGACAGGCTGCTTTTGAGATACCTGATGATGTACCTCTGCCAGCAGGTTGGGAGATGGCAAAGACATCTTCTG
GTCAGAGATACTTCTTAAATCACATCAATCAGACAACAACATGGCAGGACCCCAGGAAGGCCATGCTGTCCCAGATGAACGTCACA
GCCCCCACCAGTCCACCAGTGCAGCAGAATATGATGAACTCGGCTTCAGGTCCTCTTCCTGATGGATGGGAACAAGCCATGACTCA
GGATGGAGAAATTTACTATATAAACCATAAGAACAAGACCACCTCTTGGCTAGACCCAAGGCTTGACCCTCGTTTTGCCATGAACC
AGAGAATCAGTCAGAGTGCTCCAGTGAAACAGCCACCACCCCTGGCTCCCCAGAGCCCACAGGGAGGCGTCATGGGTGGCAGCAAC
TCCAACCAGCAGCAACAGATGCGACTGCAGCAACTGCAGATGGAGAAGGAGAGGCTGCGGCTGAAACAGCAAGAACTGCTTCGGCA
GGAGTTAGCCCTGCGTAGCCAGTTACCAACACTGGAGCAGGATGGTGGGACTCAAAATCCAGTGTCTTCTCCCGGGATGTCTCAGG
AATTGAGAACAATGACGACCAATAGCTCAGATCCTTTCCTTAACAGTGGCACCTATCACTCTCGAGATGAGGCTACAGACAGTGGA
CTAAGCATGAGCAGCTACAGTGTCCCTCGAACCCCAGATGACTTCCTGAACAGTGTGGATGAGATGGATACAGGTGATACTATCAA
CCAAAGCACCCTGCCCTCACAGCAGAACCGTTTCCCAGACTACCTTGAAGCCATTCCTGGGACAAATGTGGACCTTGGAACACTGG
AAGGAGATGGAATGAACATAGAAGGAGAGGAGCTGATGCCAAGTCTGCAGGAAGCTTTGAGTTCTGACATCCTTAATGACATGGAG
TCTGTTTTGGCTGCCACCAAGCTAGATAAAGAAAGCTTTCTTACATGGTTATAGAGCCCTCAGGCAGACTGAATTCTGCAGATCGG
CCGCGGTCATAGCTGTTTCCTGAACAGATCCCGGGTGGCATCCCTGTGACCCCTCCCCAGTGCCTCTCCTGGCCCTGGAAGTTGCC
ACTCCAGTGCCCACCAGCCTTGTCCTAATAAAATTAAGTTGCATCATTTTGTCTGACTAGGTGTCCTTCTATAATATTATGGGGTG
GAGGGGGGTGGTATGGAGCAAGGGGCAAGTTGGGAAGACAACCTGTAGGGCCTGCGGGGTCTATTGGGAACCAAGCTGGAGTGCAG
TGGCACAATCTTGGCTCACTGCAATCTCCGCCTCCTGGGTTCAAGCGATTCTCCTGCCTCAGCCTCCCGAGTTGTTGGGATTCCAG
GCATGCATGACCAGGCTCAGCTAATTTTTGTTTTTTTGGTAGAGACGGGGTTTCACCATATTGGCCAGGCTGGTCTCCAACTCCTA
ATCTCAGGTGATCTACCCACCTTGGCCTCCCAAATTGCTGGGATTACAGGCGTGAACCACTGCTCCCTTCCCTGTCCTTCTGATTT
TAAAATAACTATACCAGCAGGAGGACGTCCAGACACAGCATAGGCTACCTGGCCATGCCCAACCGGTGGGACATTTGAGTTGCTTG
CTTGGCACTGTCCTCTCATGCGTTGGGTCCACTCAGTAGATGCCTGTTGAATTGGGTACGCGGCCAGCGGCGAGCGGTATCAGCTC
ACTCAAAGGCGGTAATACGGTTATCCACAGAATCAGGGGATAACGCAGGAAAGAACATGTGAGCAAAAGGCCAGCAAAAGGCCAGG
AACCGTAAAAAGGCCGCGTTGCTGGCGTTTTTCCATAGGCTCCGCCCCCCTGACGAGCATCACAAAAATCGACGCTCAAGTCAGAG
GTGGCGAAACCCGACAGGACTATAAAGATACCAGGCGTTTCCCCCTGGAAGCTCCCTCGTGCGCTCTCCTGTTCCGACCCTGCCGC
TTACCGGATACCTGTCCGCCTTTCTCCCTTCGGGAAGCGTGGCGCTTTCTCATAGCTCACGCTGTAGGTATCTCAGTTCGGTGTAG
GTCGTTCGCTCCAAGCTGGGCTGTGTGCACGAACCCCCCGTTCAGCCCGACCGCTGCGCCTTATCCGGTAACTATCGTCTTGAGTC
CAACCCGGTAAGACACGACTTATCGCCACTGGCAGCAGCCACTGGTAACAGGATTAGCAGAGCGAGGTATGTAGGCGGTGCTACAG
AGTTCTTGAAGTGGTGGCCTAACTACGGCTACACTAGAAGAACAGTATTTGGTATCTGCGCTCTGCTGAAGCCAGTTACCTTCGGA
AAAAGAGTTGGTAGCTCTTGATCCGGCAAACAAACCACCGCTGGTAGCGGTGGTTTTTTTGTTTGCAAGCAGCAGATTACGCGCAG
AAAAAAAGGATCTCAAGAAGATCCTTTGATCTTTTCTACGGGGTCTGACGCTCAGTGGAACGAAAACTCACGTTAAGGGATTTTGG
TCATGAGATTATCAAAAAGGATCTTCACCTAGATCCTTTTAAATTAAAAATGAAGTTTTAAATCAATCTAAAGTATATATGAGTAA
CCTGAGGCTATGGCAGGGCCTGCCGCCCCGACGTTGGCTGCGAGCCCTGGGCCTTCACCCGAACTTGGGGGGTGGGGTGGGGAAAA
GGAAGAAACGCGGGCGTATTGGCCCCAATGGGGTCTCGGTGGGGTATCGACAGAGTGCCAGCCCTGGGACCGAACCCCGCGTTTAT
GAACAAACGACCCAACACCGTGCGTTTTATTCTGTCTTTTTATTGCCGTCATAGCGCGGGTTCCTTCCGGTATTGTCTCCTTCCGT
GTTTCAGTTAGCCTCCCCCTAGGGTGGGCGAAGAACTCCAGCATGAGATCCCCGCGCTGGAGGATCATCCAGCCGGC
