# Supplementary material for: TRACERx analysis identifies a role for FAT1 in regulating chromosomal instability and whole-genome doubling via Hippo signalling
Source: Nat Cell Biol. 2024 Dec 30;27(1):154–68. doi: 10.1038/s41556-024-01558-w (PMC11735399; doi:10.1038/s41556-024-01558-w)
Supplement: Supplementary file 2 — Reporting Summary [file 41556_2024_1558_MOESM2_ESM.pdf]

nature portfolio

Corresponding author(s): Charles Swanton

Last updated by author(s): 19/8/2024

Reporting Summary

Nature Portfolio wishes to improve the reproducibility of the work that we publish. This form provides structure for consistency and transparency in reporting. For further information on Nature Portfolio policies, see our Editorial Policies and the Editorial Policy Checklist.

Statistics

For all statistical analyses, confirm that the following items are present in the figure legend, table legend, main text, or Methods section.

n/a	Confirmed	
☐	☒	The exact sample size (*n*) for each experimental group/condition, given as a discrete number and unit of measurement
☐	☒	A statement on whether measurements were taken from distinct samples or whether the same sample was measured repeatedly
☐	☒	The statistical test(s) used AND whether they are one- or two-sided<br>*Only common tests should be described solely by name; describe more complex techniques in the Methods section.*
☐	☒	A description of all covariates tested
☐	☒	A description of any assumptions or corrections, such as tests of normality and adjustment for multiple comparisons
☐	☒	A full description of the statistical parameters including central tendency (e.g. means) or other basic estimates (e.g. regression coefficient) AND variation (e.g. standard deviation) or associated estimates of uncertainty (e.g. confidence intervals)
☐	☒	For null hypothesis testing, the test statistic (e.g. *F*, *t*, *r*) with confidence intervals, effect sizes, degrees of freedom and *P* value noted<br>*Give P values as exact values whenever suitable.*
☒	☐	For Bayesian analysis, information on the choice of priors and Markov chain Monte Carlo settings
☒	☐	For hierarchical and complex designs, identification of the appropriate level for tests and full reporting of outcomes
☐	☒	Estimates of effect sizes (e.g. Cohen's *d*, Pearson's *r*), indicating how they were calculated

Our web collection on statistics for biologists contains articles on many of the points above.

Software and code

Policy information about availability of computer code

Data collection	LiCOR image studio v5.0<br>Oxford Optronix GelCount<br>MicroManager 2.0<br>Microvolution plugin for FIJI<br>FIJI ImageJ v 1.54J<br>BD FACSDiva software<br>ABI QuantStudio softwareNikon NIS elements<br>The code used to determine genome doubling is available at https://github.com/amf71/ParallelGDDetect.
Data analysis	R (version 3.6.2)<br>Alignment and QC:<br>FastQC (version 0.11.8)<br>FastQ Screen (version 0.13.0)<br>bwa-mem (version 0.7.17)<br>Sambamba (version 0.7.0)<br>Picard Tools (version 2.21.9)<br>GATK (version 3.8.1)<br>Somalier (version 0.2.7)<br>Samtools (version 1.9)<br>Conpair (version 0.2)<br>Variant Calling:

SAMtools (version 1.10)
VarScan2 (version 2.4.4)
MuTect (version 1.1.7)
bam-readcount (version 0.7.4)
Annovar (version: Revision 529)
R packages used in version 3.6.3:
fst (version 0.9.4)
tidyverse (version 1.3.0)
survival (version 3.2.13)
ggplot2 (version 3.3.2)
dplyr (version 1.0.2)
tidyr (version 1.1.2)
gridExtra (version 2.3)
cowplot (version 1.1.0)
survminer (version 0.4.9)
ggpubr (version 0.4.0)
reshape2 (version 1.4.4)
tibble (version 3.0.4)
gtable (version 0.3.0)
RColorBrewer (version 1.1-2)
plyr (version 1.8.6)
ggrepel (version 0.8.2)
GenomicRanges (version 1.38.0)
rlist (version 0.4.6.2)
tidytext (version 0.2.3)
stringr (version 1.4.0)
data.table (version 1.13.2)
DiagrammR (version 1.0.1)
magrittr (version 2.0.1)
BSgenome.Hsapiens.UCSC.hg19 (version 1.4.0)
deconstructSigs (version 1.8.0)
Li-COR Image studio Lite (5.0)
Fiji (2.0.0)
FindFoci Plug-In (Herbert et al Plos ONE 2014)
FloJo (10.8.1)
Prism (9.4.1)

For manuscripts utilizing custom algorithms or software that are central to the research but not yet described in published literature, software must be made available to editors and reviewers. We strongly encourage code deposition in a community repository (e.g. GitHub). See the Nature Portfolio guidelines for submitting code & software for further information.

Data

Policy information about availability of data

All manuscripts must include a data availability statement. This statement should provide the following information, where applicable:

- Accession codes, unique identifiers, or web links for publicly available datasets
- A description of any restrictions on data availability
- For clinical datasets or third party data, please ensure that the statement adheres to our policy

The RNA-seq, whole-exome sequencing and RRBS data (in each case from the TRACERx study) used during this study have been deposited at the European Genome–phenome Archive, which is hosted by the European Bioinformatics Institute and the Centre for Genomic Regulation, under the accession codes EGAS00001006517 (RNA-seq), EGAS00001006494 (WES) and EGAS00001006523 (RRBS). Access is controlled by the TRACERx data access committee. The Genomics England lung cohort is part of the 100,000 Genomes Project whose data are held in a secure research environment and are only available to registered users, for further information on how to obtain access visit https://www.genomicsengland.co.uk/research/academic.

Research involving human participants, their data, or biological material

Policy information about studies with human participants or human data. See also policy information about sex, gender (identity/presentation), and sexual orientation and race, ethnicity and racism.

Reporting on sex and gender	The TRACERx 421 cohort consisted of 233 males and 188 females (421 patients total), corresponding to a 55:45 M:F ratio. 93% of the cohort was from a white ethnic background and the mean age of the patients was 69, ranging between 34 and 92. Written informed consent was obtained. None of the patients were compensated for their involvement in the study. See Frankell et al Nature 2023 for cohort detail.
Reporting on race, ethnicity, or other socially relevant groupings	The ethnicity breakdown for the TRACERx 421 cohort is the following:<br>White- British 371 (88%)<br>White- Irish 17 (4%)<br>White - European 13 (3%)<br>White -Other 3 (1%)<br>Mixed 4 (1%)<br>Black 1 (<1%)<br>Caribbean 4 (1%)

Indian 3 (1%)
Middle Eastern 4 (1%)
South American 1 (<1%)

Population characteristics

Please note that the study started recruiting patients in 2016, when TNM version 7 was standard of care. The up-to-date inclusion/exclusion criteria now utilizes TNM version 8.

TRACERx inclusion and exclusion criteria

Inclusion Criteria:
_Written Informed consent
_Patients ≥18 years of age, with early stage I-IIIB disease (according to TNM 8th edition) who are eligible for primary surgery.
_Histopathologically confirmed NSCLC, or a strong suspicion of cancer on lung imaging necessitating surgery (e.g. diagnosis determined from frozen section in theatre)
_Primary surgery in keeping with NICE guidelines planned
_Agreement to be followed up at a TRACERx site
_Performance status 0 or 1
_Minimum tumor diameter at least 15mm to allow for sampling of at least two tumour regions (if 15mm, a high likelihood of nodal involvement on pre-operative imaging required to meet eligibility according to stage, i.e. T1N1-3)

Exclusion Criteria:
_Any other* malignancy diagnosed or relapsed at any time, which is currently being treated (including by hormonal therapy).
_Any other* current malignancy or malignancy diagnosed or relapsed within the past 3 years**.
*Exceptions are: non-melanomatous skin cancer, stage 0 melanoma in situ, and in situ cervical cancer
**An exception will be made for malignancies diagnosed or relapsed more than 2, but less than 3, years ago only if a pre-operative biopsy of the lung lesion has confirmed a diagnosis of NSCLC.
_Psychological condition that would preclude informed consent
_Treatment with neo-adjuvant therapy for current lung malignancy deemed necessary
_Post-surgery stage IV
_Known Human Immunodeficiency Virus (HIV), Hepatitis B Virus (HBV), Hepatitis C Virus (HCV) or syphilis infection.
_Sufficient tissue, i.e. a minimum of two tumor regions, is unlikely to be obtained for the study based on pre-operative imaging

Patient ineligibility following registration
_There is insufficient tissue
_The patient is unable to comply with protocol requirements
_There is a change in histology from NSCLC following surgery, or NSCLC is not confirmed during or after surgery.
_Change in staging to IIIC or IV following surgery
_The operative criteria are not met (e.g. incomplete resection with macroscopic residual tumors (R2)). Patients with microscopic residual tumors (R1) are eligible and should remain in the study
_Adjuvant therapy other than platinum-based chemotherapy and/or radiotherapy is administered.

Recruitment

TRACERx: Patients seen with a new diagnosis of lung cancer in lung cancer units across the United Kingdom, according to the eligibility criteria above, were recruited. No selection bias has been identified to date.

Ethics oversight

The TRACERx study was approved by the NRES Committee London with the following details:
Study title: TRAcking non small cell lung Cancer Evolution through therapy (Rx)
REC reference: 13/LO/1546
Protocol number: UCL/12/0279
IRAS project ID: 138871

Note that full information on the approval of the study protocol must also be provided in the manuscript.

Field-specific reporting

Please select the one below that is the best fit for your research. If you are not sure, read the appropriate sections before making your selection.

☒ Life sciences ☐ Behavioural & social sciences ☐ Ecological, evolutionary & environmental sciences

For a reference copy of the document with all sections, see nature.com/documents/nr-reporting-summary-flat.pdf

Life sciences study design

All studies must disclose on these points even when the disclosure is negative.

Sample size

For the bioinformatics studies, the sample size of 421 patients represents the half-way point of the TRACERx longitudinal study. In total 432 tumours (1553 tumour regions) of the 421 patients were analysed in this study. TRACERx is a programme of work of multiple projects built around a single observational cohort study. It is not possible to perform a sample size calculation for each project, especially post hoc. Please see Frankell et al, Nature 2023 for detailed explanation.

For the investigations involving cell lines, a minimum of three independent experiments were performed unless otherwise stated. Measurements were taken from distinct samples except from live cell imaging experiments when the same cell was recorded over a period of time. No statistical methods were used to predetermine sample size. The statistical test types and biological n number used in each experiment are detailed in the relevant figure legends.

Data exclusions

Please see study inclusion/exclusion criteria below. Additionally, samples which fail quality control metrics including low tumor purity (<10%) were also excluded from analysis.

Please note that the study started recruiting patients in 2016, when TNM version 7 was standard of care. The up-to-date inclusion/exclusion criteria now utilizes TNM version 8.

TRACERx inclusion and exclusion criteria

Inclusion Criteria:
_Written Informed consent
_Patients ≥18 years of age, with early stage I-IIIB disease (according to TNM 8th edition) who are eligible for primary surgery.
_Histopathologically confirmed NSCLC, or a strong suspicion of cancer on lung imaging necessitating surgery (e.g. diagnosis determined from frozen section in theatre)
_Primary surgery in keeping with NICE guidelines planned
_Agreement to be followed up at a TRACERx site
_Performance status 0 or 1
_Minimum tumor diameter at least 15mm to allow for sampling of at least two tumour regions (if 15mm, a high likelihood of nodal involvement on pre-operative imaging required to meet eligibility according to stage, i.e. T1N1-3)

Exclusion Criteria:
_Any other* malignancy diagnosed or relapsed at any time, which is currently being treated (including by hormonal therapy).
_Any other* current malignancy or malignancy diagnosed or relapsed within the past 3 years**.
*Exceptions are: non-melanomatous skin cancer, stage 0 melanoma in situ, and in situ cervical cancer
**An exception will be made for malignancies diagnosed or relapsed more than 2, but less than 3, years ago only if a pre-operative biopsy of the lung lesion has confirmed a diagnosis of NSCLC.
_Psychological condition that would preclude informed consent
_Treatment with neo-adjuvant therapy for current lung malignancy deemed necessary
_Post-surgery stage IV
_Known Human Immunodeficiency Virus (HIV), Hepatitis B Virus (HBV), Hepatitis C Virus (HCV) or syphilis infection.
_Sufficient tissue, i.e. a minimum of two tumor regions, is unlikely to be obtained for the study based on pre-operative imaging

Patient ineligibility following registration
_There is insufficient tissue
_The patient is unable to comply with protocol requirements
_There is a change in histology from NSCLC following surgery, or NSCLC is not confirmed during or after surgery.
_Change in staging to IIIC or IV following surgery
_The operative criteria are not met (e.g. incomplete resection with macroscopic residual tumors (R2)). Patients with microscopic residual tumors (R1) are eligible and should remain in the study
_Adjuvant therapy other than platinum-based chemotherapy and/or radiotherapy is administered.

Replication

TRACERx is a prospective longitudinal study. As such, the results shown are not the result of an experimental set up. This is the half-way point of the TRACERx study and reflects hypothesis-generating analysis.

For the investigations involving cell lines, a minimum of three independent experiments were performed unless otherwise stated. N number indicating biological replicates are available in the manuscripts. Measurements were taken from distinct samples except from live cell imaging experiments when the same cell was recorded over a period of time.

Randomization

No randomization was conducted. Randomization is not applicable for TRACERx data because this is an observational study. Randomization is not applicable for experiments involving cell lines.

Blinding

Not applicable for this study, except for fixed cell imaging studies. For experiments comprising imaging analysis, laser intensity and channel intensity were standardized using the control cells as a reference. Initially, microscope slides were renamed and blinded before microscopy imaging and analysis so that the sample identity is not known at the point of imaging. However, since FAT1 loss induces cell morphology change this is not strictly necessary as the identity become rather obvious during imaging. For genomics data analysis (TRACERx , TCGA or Genomics England) blinding is not applicable. For flow cytometry and WBs the experiments are not blinded.

Reporting for specific materials, systems and methods

We require information from authors about some types of materials, experimental systems and methods used in many studies. Here, indicate whether each material, system or method listed is relevant to your study. If you are not sure if a list item applies to your research, read the appropriate section before selecting a response.

Materials & experimental systems

n/a	Involved in the study	
☐	☒	Antibodies
☐	☒	Eukaryotic cell lines
☒	☐	Palaeontology and archaeology
☒	☐	Animals and other organisms
☒	☐	Clinical data
☒	☐	Dual use research of concern
☒	☐	Plants

Methods

n/a	Involved in the study	
☒	☐	ChIP-seq
☐	☒	Flow cytometry
☒	☐	MRI-based neuroimaging

Antibodies

Antibodies used

The specificity of all the antibodies were validated by the supplier, with the relevant data page listed below.

Santa Cruz SC-56324 mouse anti mcm7 used in facs
https://www.scbt.com/p/mcm7-antibody-47dc141-human
abcam ab32053 rabbit anti cyclin b1 used in facs
https://www.abcam.com/en-gb/products/primary-antibodies/cyclin-b1-antibody-y106-ab32053
Thermo Fisher (clickIT EdU kit) c10634 anti edu used in facs and IF(against nucleotides, not protein)
https://www.thermofisher.com/order/catalog/product/C10634?SID=srch-hj-c10634
Abcam 6326 rat anti Anti-BrdU for CIdU used in fibre assay (against nucleotides, not protein, widely used in multiple DDR papers)
https://www.abcam.com/en-gb/products/primary-antibodies/brdu-antibody-bu1-75-icr1-proliferation-marker-ab6326
BD (clone B44) 347580 mouse anti Anti-BrdU for IDU used in fibre assay(against nucleotides, not protein, widely used in multiple DDR papers)
https://www.sigmaaldrich.com/GB/en/product/sigma/b2531?utm_source=google&utm_medium=cpc&utm_campaign=21473730186&utm_content=165772576758&gclid=CjwKCAjwoJa2BhBPEiwA0l0ImFXQUeI5sudLYDRU70jmLQAgn_INvq-U6YK6kxL5jDO2_5G-d8rIoBoCE90QAvD_BwE
MerckMillipore 05-636 mouse anti γH2A.X used in high content screening and IF
https://www.merckmillipore.com/GB/en/product/Anti-phospho-Histone-H2A.X-Ser139-Antibody-clone-JBW301,MM_NF-05-636?ReferrerURL=https%3A%2F%2Fwww.google.com%2F
Santa Cruz sc-22760 rabbit anti 53bp1 used in high content screening – discontinued due to Santa Cruz losing animal license.
https://www.scbt.com/p/53bp1-antibody-h-300
Santa Cruz sc-8349 rabbit anti rad51 used in high content screening– discontinued due to Santa Cruz losing animal license.
https://www.scbt.com/p/rad51-antibody-h-92
Abcam ab87277 rabbit anti rpaps4s8 used in high content screening
https://www.abcam.com/en-gb/products/primary-antibodies/rpa32-rpa2-phospho-s4-s8-antibody-ab87277
Abcam ab109394 rabbit anti rpa pT21 used in high content screening
https://www.abcam.com/en-gb/products/primary-antibodies/rpa32-rpa2-phospho-t21-antibody-epr28462-ab109394
Abcam ab63801 rabbit anti rad51 used in IF
https://www.abcam.com/en-gb/products/primary-antibodies/rad51-antibody-ab63801
Cell Signaling 4526 mouse anti patmS1981 used in IF
https://www.cellsignal.com/products/primary-antibodies/phospho-atm-ser1981-10h11-e12-mouse-mab/4526
Novus Biologicals nb100-305 rabbit anti 53BP1 used in IF
https://www.bio-techne.com/p/antibodies/53bp1-antibody_nb100-305
Abcam ab16780 mouse anti BRCA1 used in IF
https://www.abcam.com/en-gb/products/primary-antibodies/brca1-antibody-ms110-ab16780
Francis Crick institute (in house monoclonal) E43.2 mouse anti cyclin A used in IF
NB: This is the same clone sold by Santa Cruz Sc-53229, and was used in multiple papers.
https://www.scbt.com/p/cyclin-a-antibody-e43-2?srsltid=AfmBOoqon6i6jwR5N5L9n1EpI04yAxIx6OM_Udav79LNDDhAwsEU56Te
Cell Signaling 9201 rabbit anti CtIP used in IF
https://www.cellsignal.com/products/primary-antibodies/ctip-d76f7-rabbit-mab/9201
ImmunoVision HCT-0100 human anti CREST used in IF
https://immunovision.com/index.php/autoimmune-polyclonal-antibodies/autoimmune-positive-controls/
Cell Signaling 58982 rabbit anti CENPF used in IF
https://www.cellsignal.com/products/primary-antibodies/cenp-f-d6x4l-rabbit-mab/58982
Cell Signaling 8878 anti Alexa Fluor 488 phalloidin used in if
https://www.cellsignal.com/products/buffers-dyes/alexa-fluor-488-phalloidin/8878
Cell Signaling 8940 anti Alexa Fluor 647 phalloidin used in IF
https://www.cellsignal.com/products/buffers-dyes/alexa-fluor-647-phalloidin/8940
Atlas antibodies AMAb90562 mouse anti emerin used in if
https://www.atlasantibodies.com/products/primary-antibodies/precisa-monoclonals/anti-emd-antibody-amab90562-100ul/?language=en
Santa Cruz SC-101199 mouse anti Yap1 used in if and wb
https://www.scbt.com/p/yap-antibody-63-7
Novus Biological NB100-182 rabbit anti FancD2 used in if and wb
https://www.bio-techne.com/p/antibodies/fancd2-antibody_nb100-182
Cell Signaling 65344 rabbit anti Ubr5 used in wb
https://www.cellsignal.com/products/primary-antibodies/ubr5-d6o8z-rabbit-mab/65344
Novus Biologicals NBP2-32275 rabbit anti Fat1 used in wb
https://www.bio-techne.com/p/antibodies/fat1-antibody_nbp2-32275#reviews

Proteintech 27071-1-AP rabbit anti Rad21 used in wb
https://www.ptglab.com/products/RAD21-Antibody-27071-1-AP.htm
Cell Signaling 9718 rabbit anti γH2A.X used in wb
https://www.cellsignal.com/products/primary-antibodies/phospho-histone-h2a-x-ser139-20e3-rabbit-mab/9718
Proteintech 10398-1-AP rabbit anti Bap1 used in wb
https://www.ptglab.com/products/BAP1-Antibody-10398-1-AP.htm
Bethyl A300-363A-T rabbit anti Crebbp used in wb
NB: Bethyl Laboratories was rebranded as Fortis Life Science
https://www.fortislife.com/products/primary-antibodies/rabbit-anti-cbp-antibody/BETHYL-A300-363
Francis Crick insitute (in house monoclonal) clone 12AC5 mouse anti ha used in wb
*NB: In house monoclonal antibody. It is however the same clone as
https://www.sigmaaldrich.com/GB/en/product/roche/roaha?srsltid=AfmBOor8dii5mITqC6Z-h7XNiOfy_COduOp1bRgZnUHPEz8eKL2Dbgcg
Proteintech 25241-1-AP rabbit anti ncoa6 used in wb
https://www.ptglab.com/products/NCOA6-Antibody-25241-1-AP.htm
Abcam ab1791 rabbit anti Histone H3 used in wb
https://www.abcam.com/en-gb/products/primary-antibodies/histone-h3-antibody-nuclear-marker-and-chip-grade-ab1791
Abcam ab9485 rabbit anti gapdh used in wb
https://www.abcam.com/en-gb/products/primary-antibodies/gapdh-antibody-loading-control-ab9485
Bethyl A300-767A rabbit anti pkap1 used in wb
NB: Bethyl Laboratories was rebranded as Fortis Life Science
https://www.fortislife.com/products/primary-antibodies/rabbit-anti-phospho-kap-1-s824-antibody/BETHYL-A300-767
Abcam ab22758 rabbit anti nucleolin used in wb
https://www.abcam.com/en-gb/products/primary-antibodies/nucleolin-antibody-ab22758
Cell Signaling 2661 rabbit anti pChk2 Thr68 used in wb
https://www.cellsignal.com/products/primary-antibodies/phospho-chk2-thr68-antibody/2661
Cell Signaling 3477 rabbit anti Lats1 used in wb
https://www.cellsignal.com/products/primary-antibodies/lats1-c66b5-rabbit-mab/3477
Cell Signaling 5888 rabbit anti Lats2 used in wb
https://www.cellsignal.com/products/primary-antibodies/lats2-d83d6-rabbit-mab/5888
Cell Signaling 2349 rabbit anti pchk1 s296 used in wb
https://www.cellsignal.com/products/primary-antibodies/phospho-chk1-ser296-antibody/2349
Cell Signaling 2348 rabbit anti pchk1 s345 used in wb
https://www.cellsignal.com/products/primary-antibodies/phospho-chk1-ser345-133d3-rabbit-mab/2348
Cell Signaling 2360 mouse anti chk1 used in wb
https://www.cellsignal.com/products/primary-antibodies/chk1-2g1d5-mouse-mab/2360
Cell Signaling 9286 mouse anti p53s15 used in wb
https://www.cellsignal.com/products/primary-antibodies/phospho-p53-ser15-16g8-mouse-mab/9286
Cell Signaling 2527 rabbit anti p53 used in wb
https://www.cellsignal.com/products/primary-antibodies/p53-7f5-rabbit-mab/2527
Abcam ab6046 rabbit anti beta tubulin used in wb
https://www.abcam.com/en-gb/products/primary-antibodies/beta-tubulin-antibody-loading-control-ab6046
Affinity DF2444 rabbit anti e2f7 used in wb
https://www.affbiotech.com/pdf?id=6525
Cell Signaling 29495 rabbit anti active yap1 used in wb
https://www.cellsignal.com/products/primary-antibodies/non-phospho-active-yap-ser127-e6u8z-rabbit-mab/29495
Cell Signaling 13619 rabbit anti yap1 ps397 used in wb
https://www.cellsignal.com/products/primary-antibodies/phospho-yap-ser397-d1e7y-rabbit-mab/13619
Cell Signaling 4911 rabbit anti yap1 ps127 used in wb
https://www.cellsignal.com/products/primary-antibodies/phospho-yap-ser127-antibody/4911
Santa Cruz sc-73614 mouse anti vinculin used in wb
https://www.scbt.com/p/vinculin-antibody-7f9
Cell Signaling 6943 rabbit anti Src pTyr416 used in wb
https://www.cellsignal.com/products/primary-antibodies/phospho-src-family-tyr416-d49g4-rabbit-mab/6943
Cell Signaling 65890 rabbit anti yes used in wb
https://www.cellsignal.com/products/primary-antibodies/yes-d9p3e-rabbit-mab/65890
Cell Signaling 9154 rabbit anti phospho mek1/2 Ser217/221 used in wb
https://www.cellsignal.com/products/primary-antibodies/phospho-mek1-2-ser217-221-41g9-rabbit-mab/9154
Cell Signaling 4376 rabbit anti pErk1/2 T202/Y204 used in wb
https://www.cellsignal.com/products/primary-antibodies/phospho-p44-42-mapk-erk1-2-thr202-tyr204-20g11-rabbit-mab/4376
Cell Signaling 4695 rabbit anti total erk1/2 used in wb
https://www.cellsignal.com/products/primary-antibodies/p44-42-mapk-erk1-2-137f5-rabbit-mab/4695

Validation

The antibodies used have been validated accordingly to manufacturer's instructions. Molecular size markers have been included on each of the western blots, and the molecular weights for each of the antibodies were validated as per manufacturer's datasheets detailed above.

Eukaryotic cell lines

Policy information about cell lines and Sex and Gender in Research

Cell line source(s)

H1944 (lung, female), H1650 (lung, male), H1792 (lung, male), HCC4006(lung, male), A549 (CCL-185, lung male) and U2OS (HTB-96, osteosarcomam female) cells were obtained from Cell Services at the Francis Crick Institute, UK.

	RPE1-FUCCI-H2B-Turquoise cells have been described previously and were a kind gift from John Diffley (The Francis Crick Institute, UK). U2OS-HA-ER-AsiSI cells have been described previously 18 and were a kind gift from Gaelle Legube (Univ. Paul Sabatier, Toulouse, France). T2P-ER-KRAS-V12 cells (referred to as T2P) have been described previously and were a kind gift from Julian Downward (The Francis Crick Institute, UK). RPE1 TP53WT and RPE1 TP53 KO cells have been described previously. HCT116 (male, colon) iRFP cell lines have been described previously and were a kind gift from Karen Vousden (The Francis Crick Institute, UK).
Authentication	For Cell Authentication we use STR (Short Tandem Repeat) Profiling for all our Human cell lines using the Promega PowerPlex16HS system. This profile is compared back to any available on commercial cell banks (such as ATCC). We confirm the species is correct using a primer system based on the Cytochrome C Oxidase Subunit 1 gene from mitochondria – we call this test Species ID. Authentication is carried out in house within the Francis Crick Institute.
Mycoplasma contamination	For Mycoplasma screening we primarily use two different tests – Agar Culture (which involves culturing any mycoplasma that may be present in the cell culture on specialised agar) and Fluorescent staining using the Hoescht Stain. A third detection method, the PCR mycoplasma test (ATCC), is used on occasion when a rapid result is required.Mycoplasma test is carried out in house routinely within the Francis Crick Institute. No mycoplasma contamination was detected.
Commonly misidentified lines (See ICLAC register)	No Commonly misidentified lines were used in this study.

Flow Cytometry

Plots

Confirm that:

☒ The axis labels state the marker and fluorochrome used (e.g. CD4-FITC).

☒ The axis scales are clearly visible. Include numbers along axes only for bottom left plot of group (a 'group' is an analysis of identical markers).

☒ All plots are contour plots with outliers or pseudocolor plots.

☒ A numerical value for number of cells or percentage (with statistics) is provided.

Methodology

Sample preparation	For HAC assay, HT1080 cells containing EGFP-HAC were maintained in 6 µg/ml blasticidin S selection media. For siRNA treatment, 100000 cells/well were seeded in 6 well plates before the day of the experiment. Cells were transfected with each siRNAs using the Lulluby reagent. Cells were grown without blasticidin S selection for 14 days. Silencing efficiency was monitored by Western blot analysis. On Day 14, cells were collected and analyzed by flow cytometry to determine the proportion of cells that gained or lost EGFP fluorescence. All experiments were carried out in triplicate.<br><br>For ImageStream FISH experiments, transfected cells were processed for FISH in suspension as described in Worrall et al, Cell Reports 2018. Briefly, following transfection, cells were harvested and fixed with freshly prepared 3:1 methanol-glacial acetic acid. Cells were subsequently hybridized with chromosome 15 satellite enumeration probe (LPE015G, Cytocell) performed in a thermocycler under the following conditions: 65°C (2 hr pre annealing), 80°C (5 min), 37°C (16 hr), prior to analysis on an ImageStream X Mk II (Amnis).<br><br>For the DR-GFP assay, samples were prepared as detailed in Seluanov et al JoVE 2010. For ploidy determination experiments, cells were trypsinized, washed in PBS and then fixed in 70% ethanol. Cells were then washed again, and treated with staining buffer containing propidium iodide and 100 µg/ml RNAse A.<br><br>For MCM7 loading assay, PC9 cells or RPE1 cells were trypsinized and treated with CSK buffer (10 mM HEPES pH7.9, 100 mM NaCl, 3 mM MgCl2, 1 mM EGTA, 300 mM sucrose, 1%BSA, 0.2% Triton X-100, 1 mM DTT) on ice for 5 minutes to remove soluble proteins. Cells were washed with 1%BSA in PBS and fixed in 4% PFA (Thermo Fisher Scientific for 10 minutes). Cells were then washed, pelleted and permeabilised in 70% EtOH for 15 minutes. MCM7 staining was performed using a mouse anti-MCM7 antibody (Santa Cruz, sc-56324) followed by an Alexa 594 goat-anti-mouse antibody (A11007, Invitrogen). DNA content was stained using 1 ug/ml DAPI supplemented with 100 ug/ml RNAse A. For the EdU incorporation assay and cyclin B loading assays, the Click-iT Plus EdU Alexa Fluor 647 Flow Cytometry Assay kit (Thermo Fisher) was used for EdU incorporation assays. Cells were incubated with 10 uM EdU for 30 minutes before being fixed and processed as per the manufacturer's instructions. Cyclin B1 antibody was obtained from Abcam (ab32053, Abcam). DNA content was stained in staining buffer (1 µg/ml DAPI, 100 µg/ml RNAse A in PBS).<br><br>Cell doublets were excluded from all analyses.
Instrument	Flow cytometry analyses were carried out on a BD Fortessa. All cell-sorting experiments were carried out on a BD Aria Fusion or Aria III. Image Stream MkII flow cytometer (Merck) is used for flow-FISH experiments.
Software	FlowJo10.4.2 was used for flow analysis
Cell population abundance	Not applicable. Cell lines were used for flow analysis. Gating strategy described as below.

Gating strategy

All samples were first gated to exclude cellular debris using SSC-A/FSC-A and a diagonal gate using FSC-H/FSC-A was then used to exclude doublets. Whenever applicable, a diagonal gate using nucleic acid stains (PE-H/PE-A) was further used to refine doublet gating. Fluorescence minus one controls were used to set up EdU incorporation experiments, DNA ploidy analysis, MCM7 loading assays and cyclin B loading experiments. For DR-GFP HR reporter assays, No-ISceI control and mCherry co-transfection control were used to refine the gating strategy.
Gating strategy examples will be included in the Supp. information in the revised manuscript.

☒ Tick this box to confirm that a figure exemplifying the gating strategy is provided in the Supplementary Information.
