# Supplementary material for: TRACERx analysis identifies a role for FAT1 in regulating chromosomal instability and whole-genome doubling via Hippo signalling
Source: Nat Cell Biol. 2024 Dec 30;27(1):154–68. doi: 10.1038/s41556-024-01558-w (PMC11735399; doi:10.1038/s41556-024-01558-w)
Supplement: Supplementary file 17 — Statistical source data. [file 41556_2024_1558_MOESM17_ESM.zip › extended fig1/ext fig1A source/docs/README.docx]

## Required Libraries

This project needs the following packages installed: ProjectTemplate randomForest pheatmap data.table ggplot2 gridExtra stringr dplyr

## Set Gene List

The reference gene list can be set in src/assignGeneList.R. It sets the variable genesToUse. This is referenced by screen_get_heatmaps.R, hac_getSSMD_TP53_NT_plots.R, hac_getDual_flashlight_plots.R, hac_getSSMD_replicate_plots.R

## Screen Analysis

All screen raw data is stored in the data/rawData directory.

### screen_runRandomForestAssignments.R

The random forest hit calculations are run using src/screen_runRandomForestAssignment.R and read data from the data/rawData direcory. This script outputs several PCA seperation plots of controls within each screen/condition. These are contained within graphs/screen directory. It also outputs files desribing the models and controls used in training the models within the outputs/ directory named modelScores.txt and modelScores_H2AX.txt. The main output of the script is the geneClassTable.txt and geneClassTable_H2AX.txt which contain the binary class assignment from the random models for each gene in each screen/condition/cell line. These two files are used in the screen_get_heatmaps.R script.

### screen_get_heatmaps.R

The heatmaps are produced from the data outputted from the script above. Heatmaps are saved to the outputs/randomForest_heatmaps directory. The script reads data from the geneClassTable.txt and geneClassTable_H2AX.txt within the outputs/ directory. This references the gene list and only include referenced genes in the output heatmaps.
