# Supplementary figures and images for: TRACERx analysis identifies a role for FAT1 in regulating chromosomal instability and whole-genome doubling via Hippo signalling
Source: Nat Cell Biol. 2024 Dec 30;27(1):154–68. doi: 10.1038/s41556-024-01558-w (PMC11735399; doi:10.1038/s41556-024-01558-w)

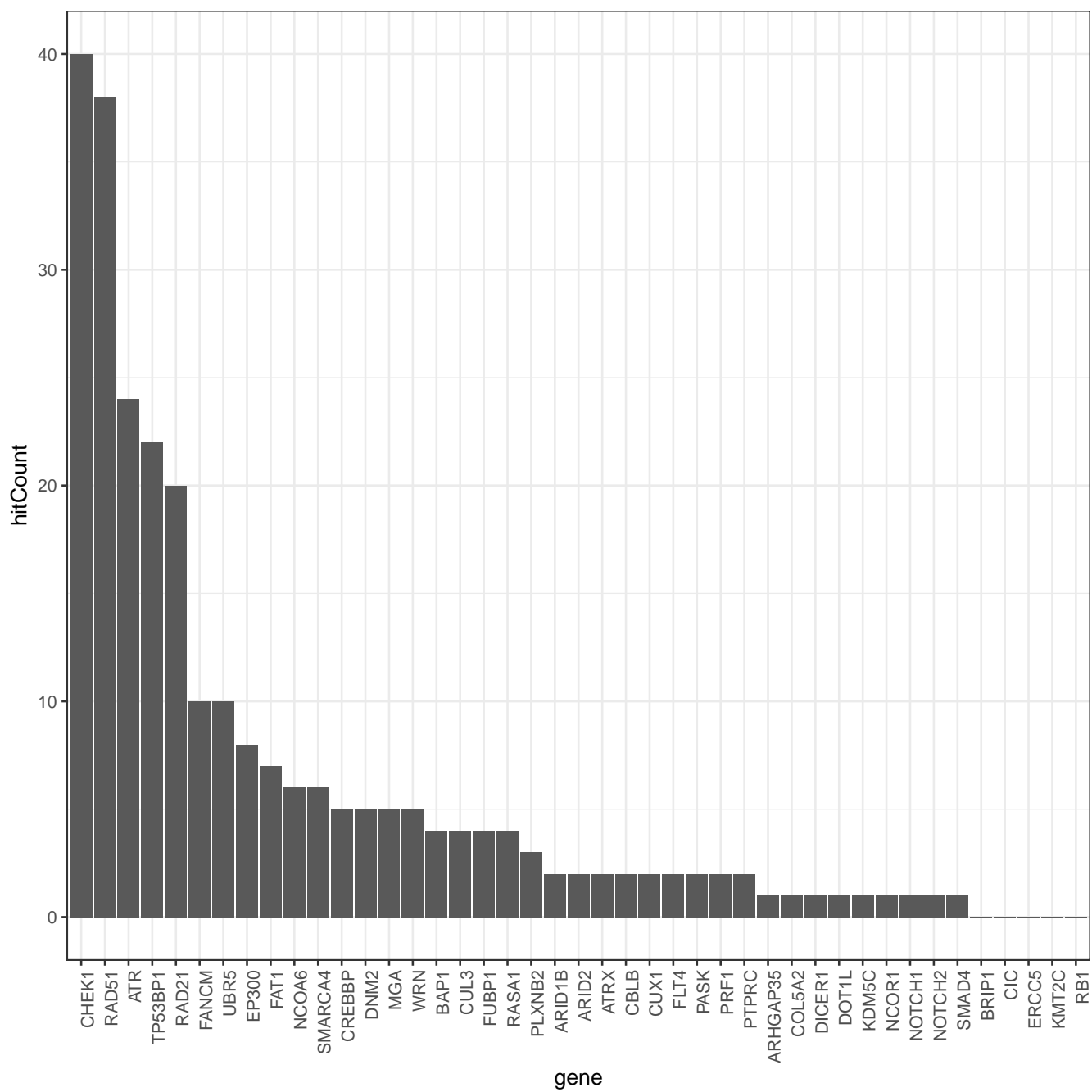

Supplement: Supplementary file 17 — Statistical source data. [file 41556_2024_1558_MOESM17_ESM.zip › extended fig1/ext fig1A source/graphs/cummulative_hitPerGene_BarPlot.pdf]

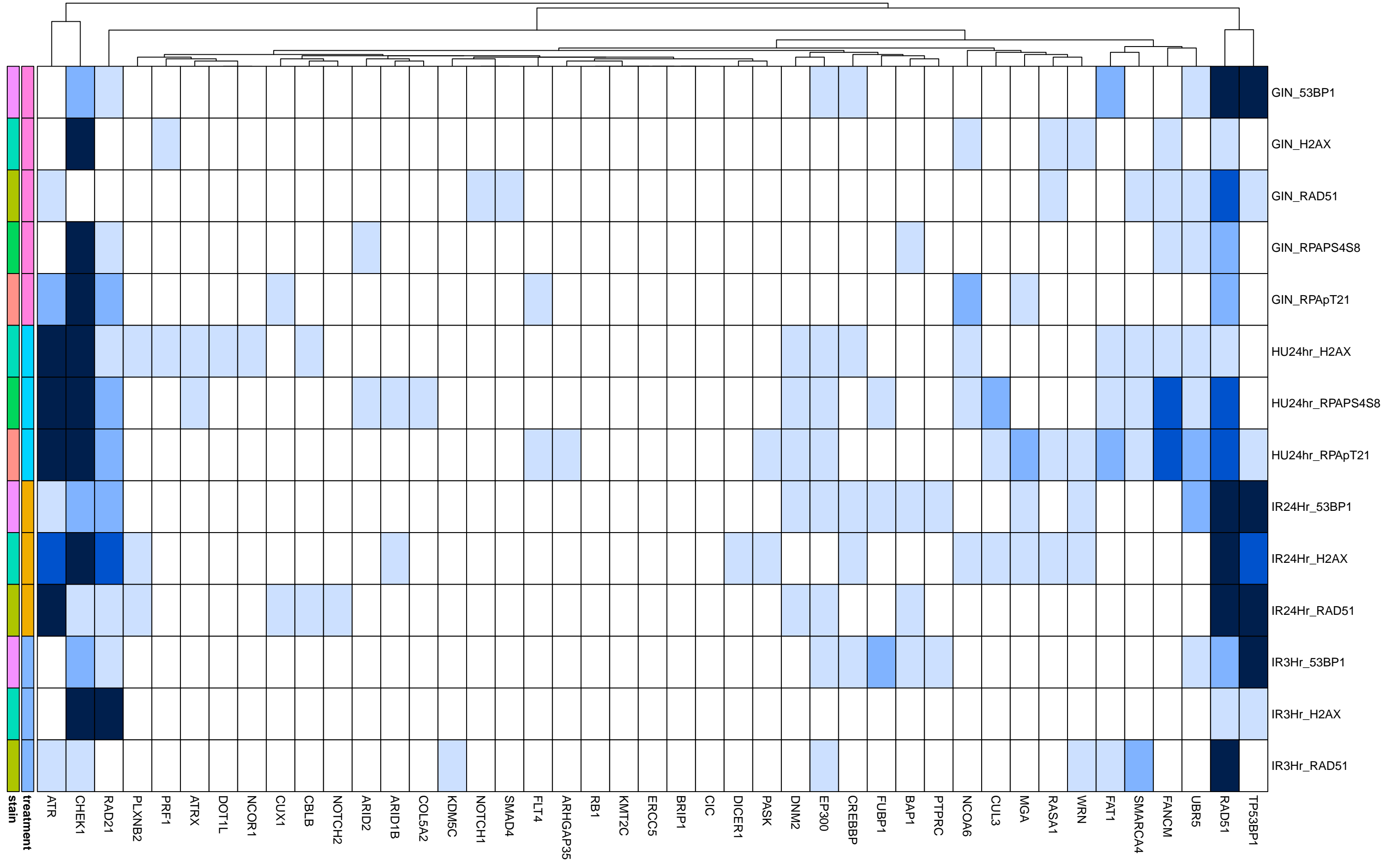

Supplement: Supplementary file 17 — Statistical source data. [file 41556_2024_1558_MOESM17_ESM.zip › extended fig1/ext fig1A source/outputs/randomForest_heatmaps/heatmap_cellLine_clustered.pdf]

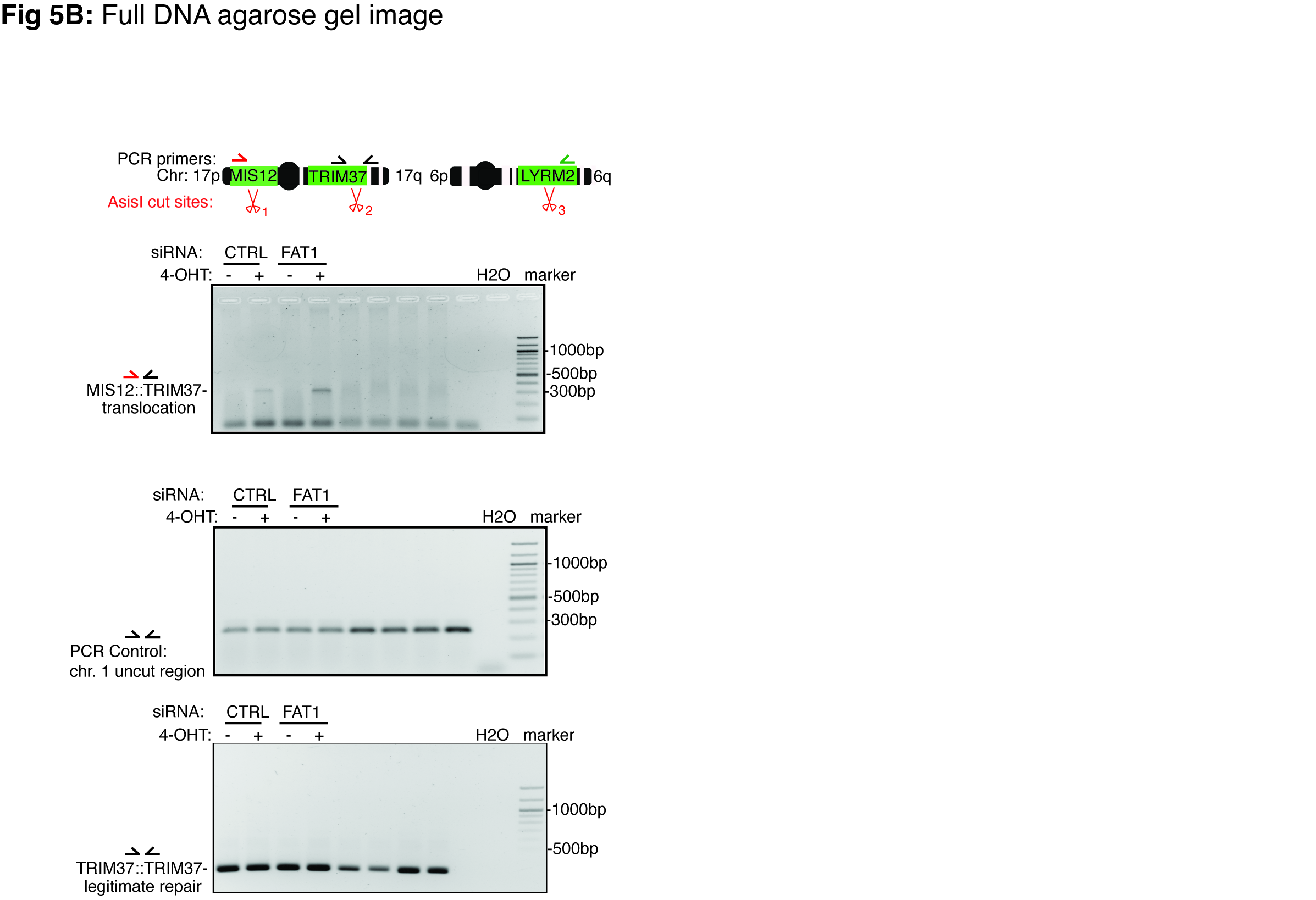

Supplement: Supplementary file 27 — Unprocessed gels or blots. [file 41556_2024_1558_MOESM27_ESM.tif]

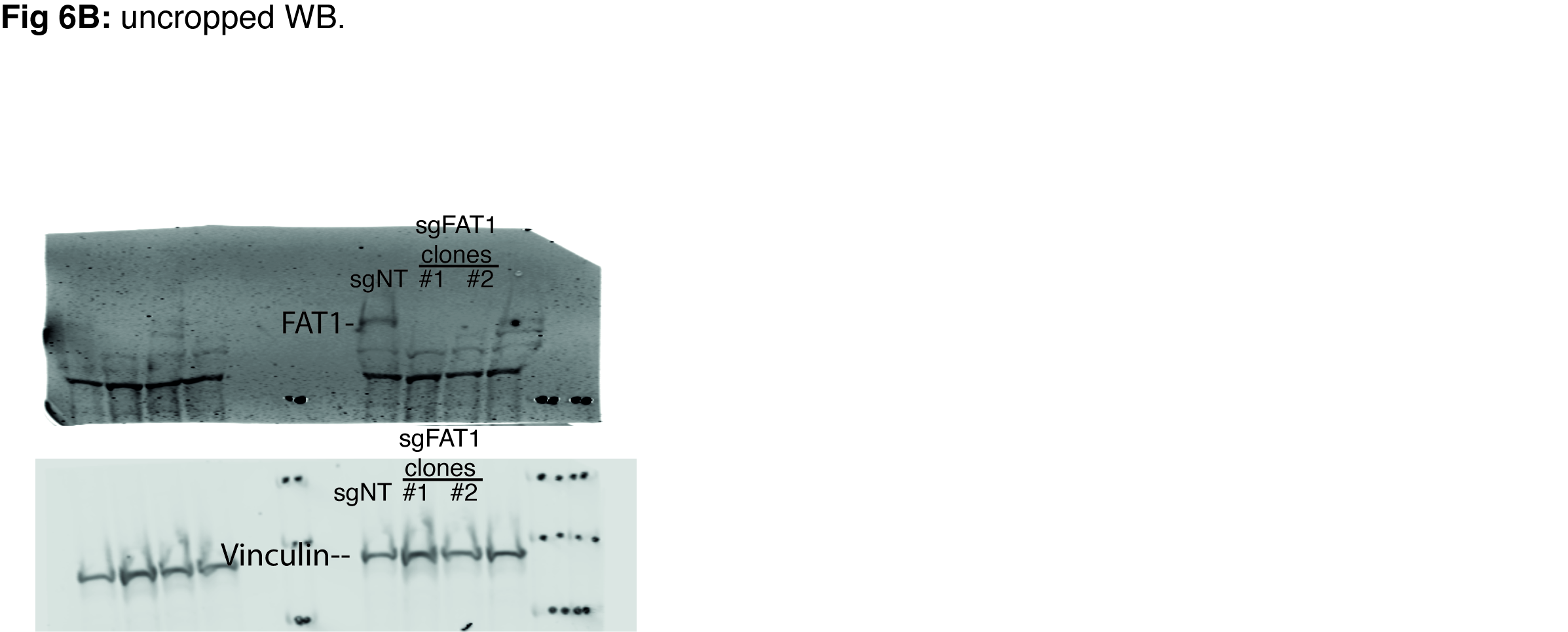

Supplement: Supplementary file 28 — Unprocessed gels or blots. [file 41556_2024_1558_MOESM28_ESM.tif]

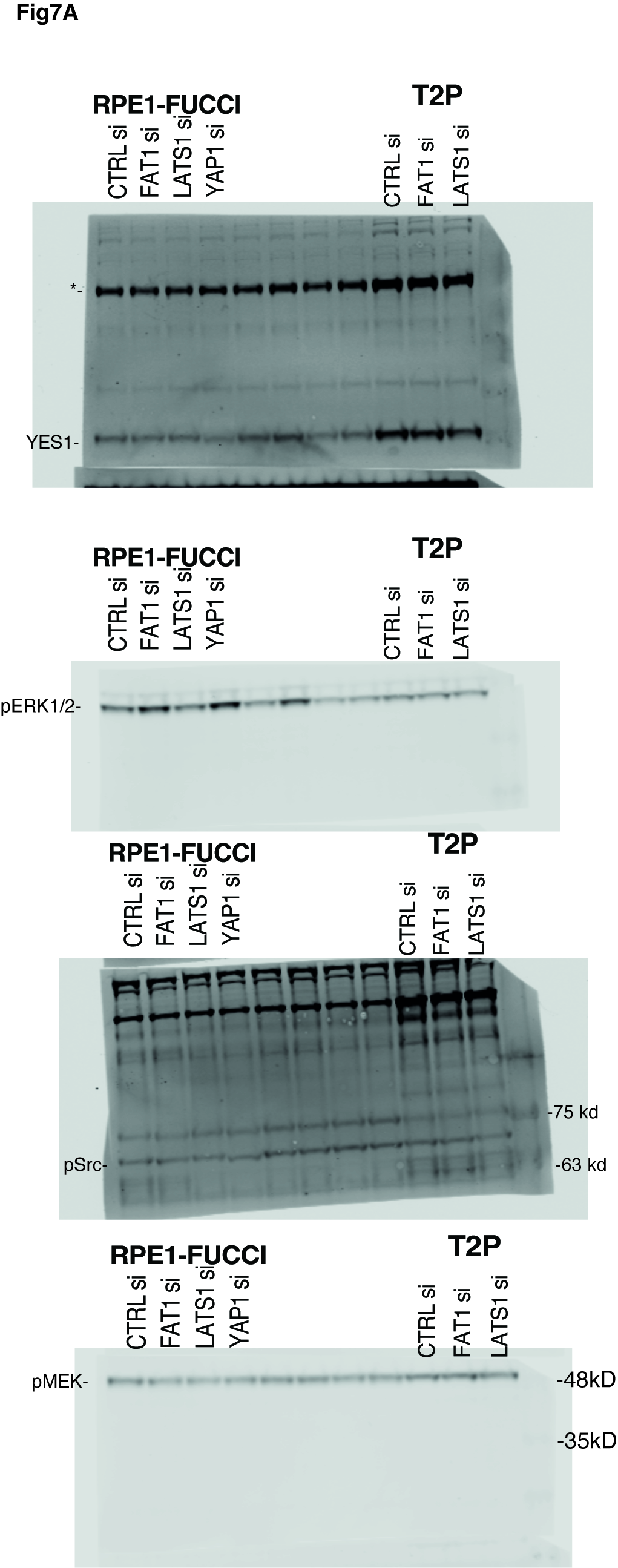

Supplement: Supplementary file 29 — Unprocessed gels or blots. [file 41556_2024_1558_MOESM29_ESM.tif]

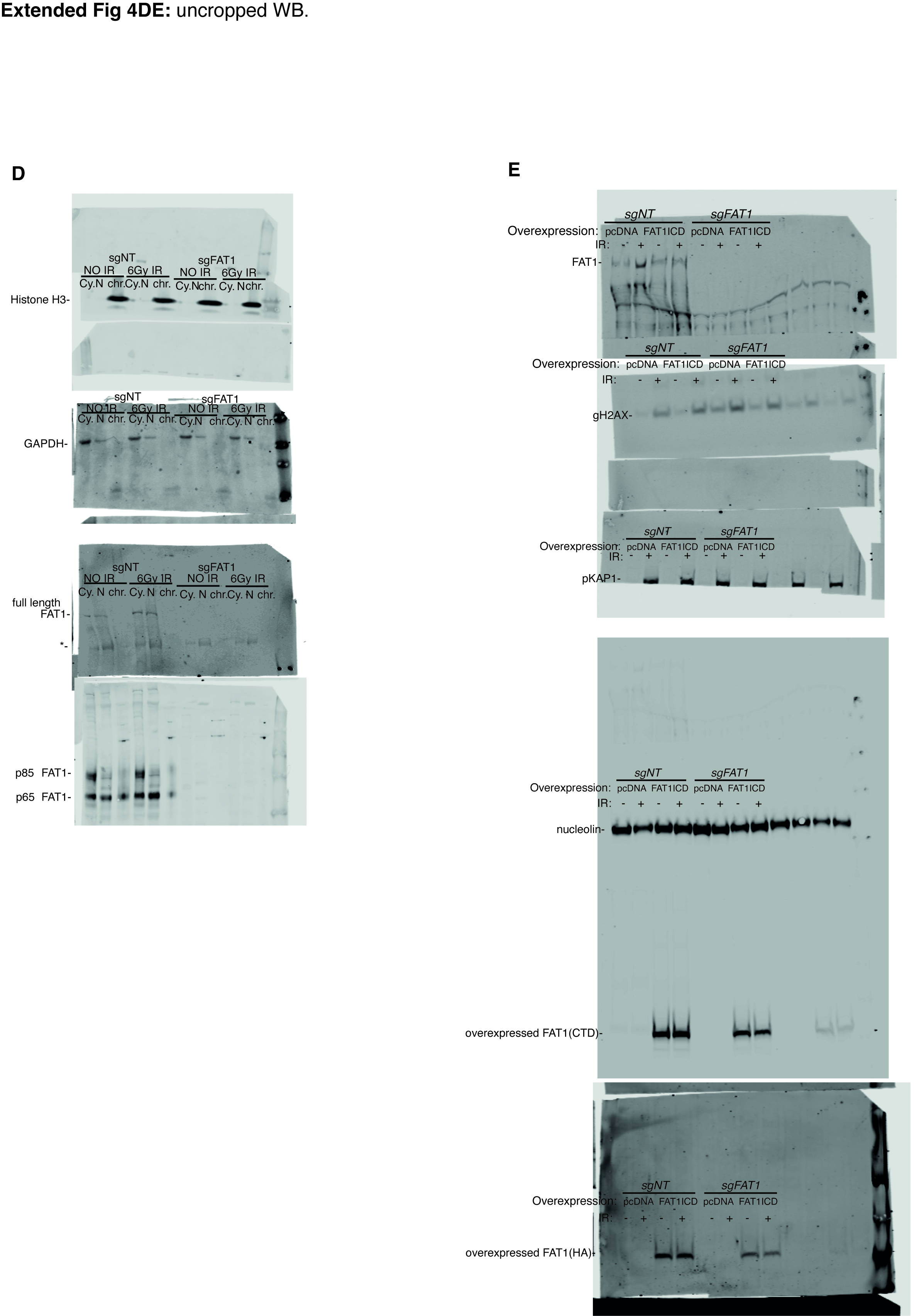

Supplement: Supplementary file 30 — Unprocessed gels or blots. [file 41556_2024_1558_MOESM30_ESM.tif]

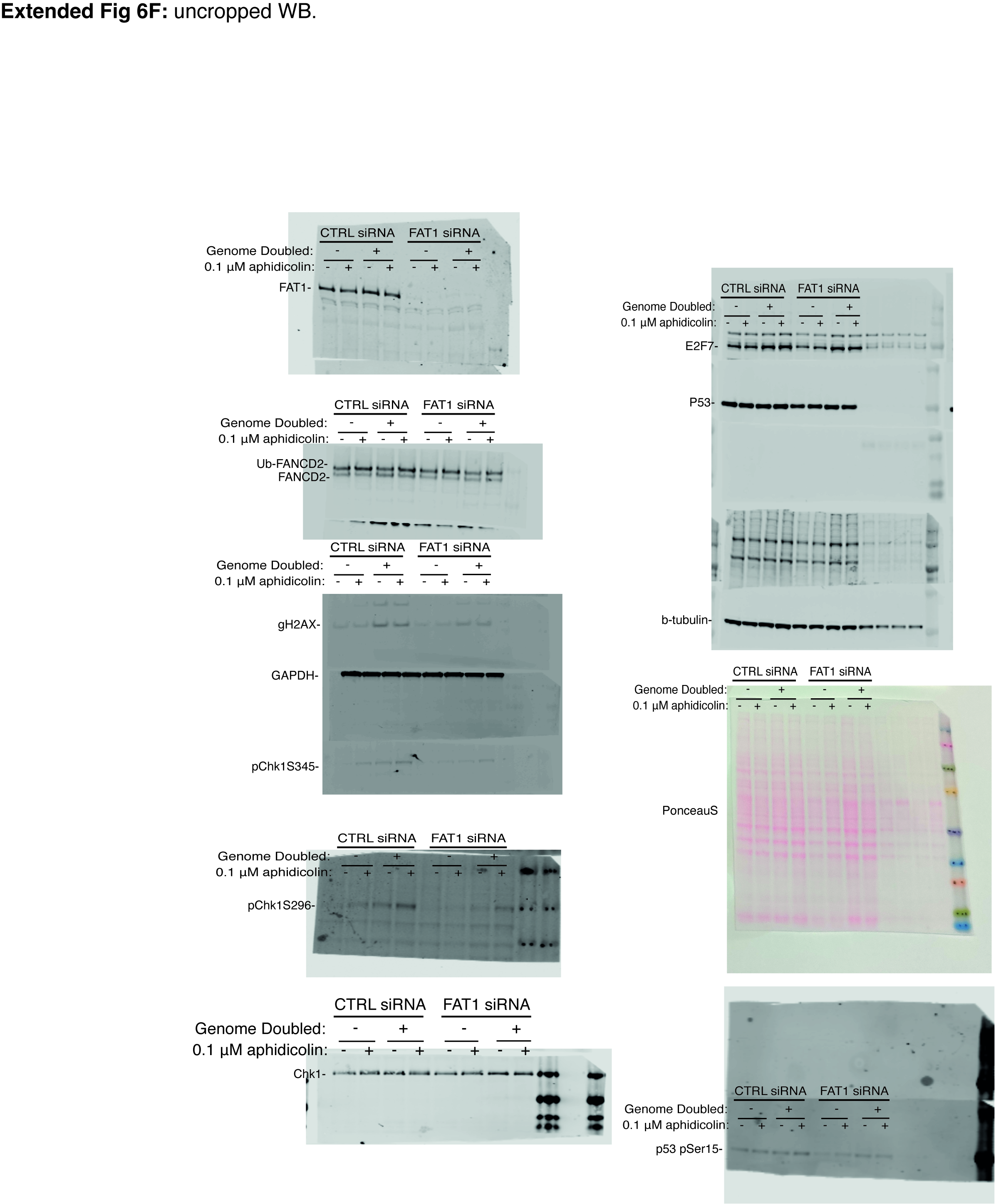

Supplement: Supplementary file 31 — Unprocessed gels or blots. [file 41556_2024_1558_MOESM31_ESM.tif]

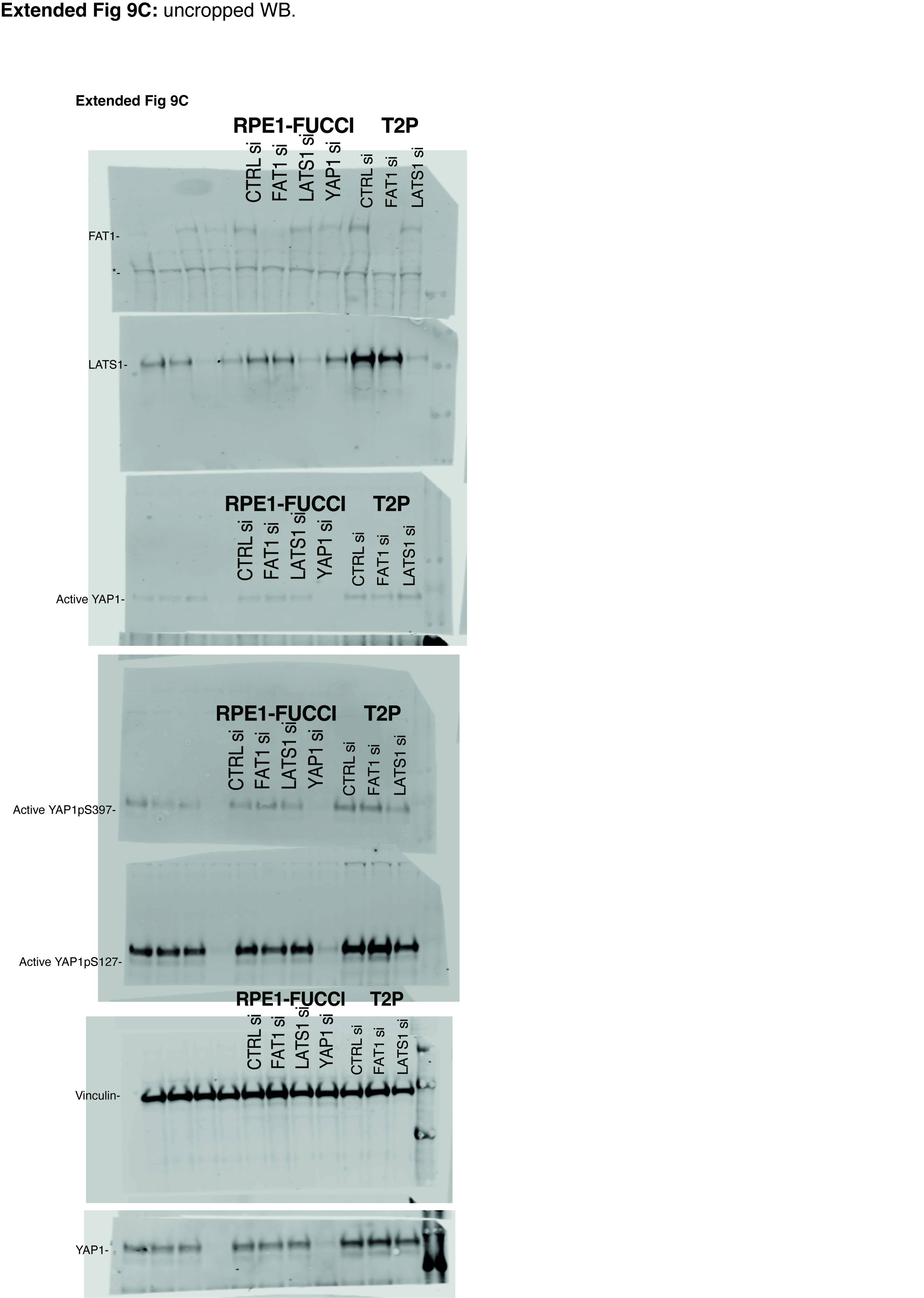

Supplement: Supplementary file 32 — Unprocessed gels or blots. [file 41556_2024_1558_MOESM32_ESM.tif]

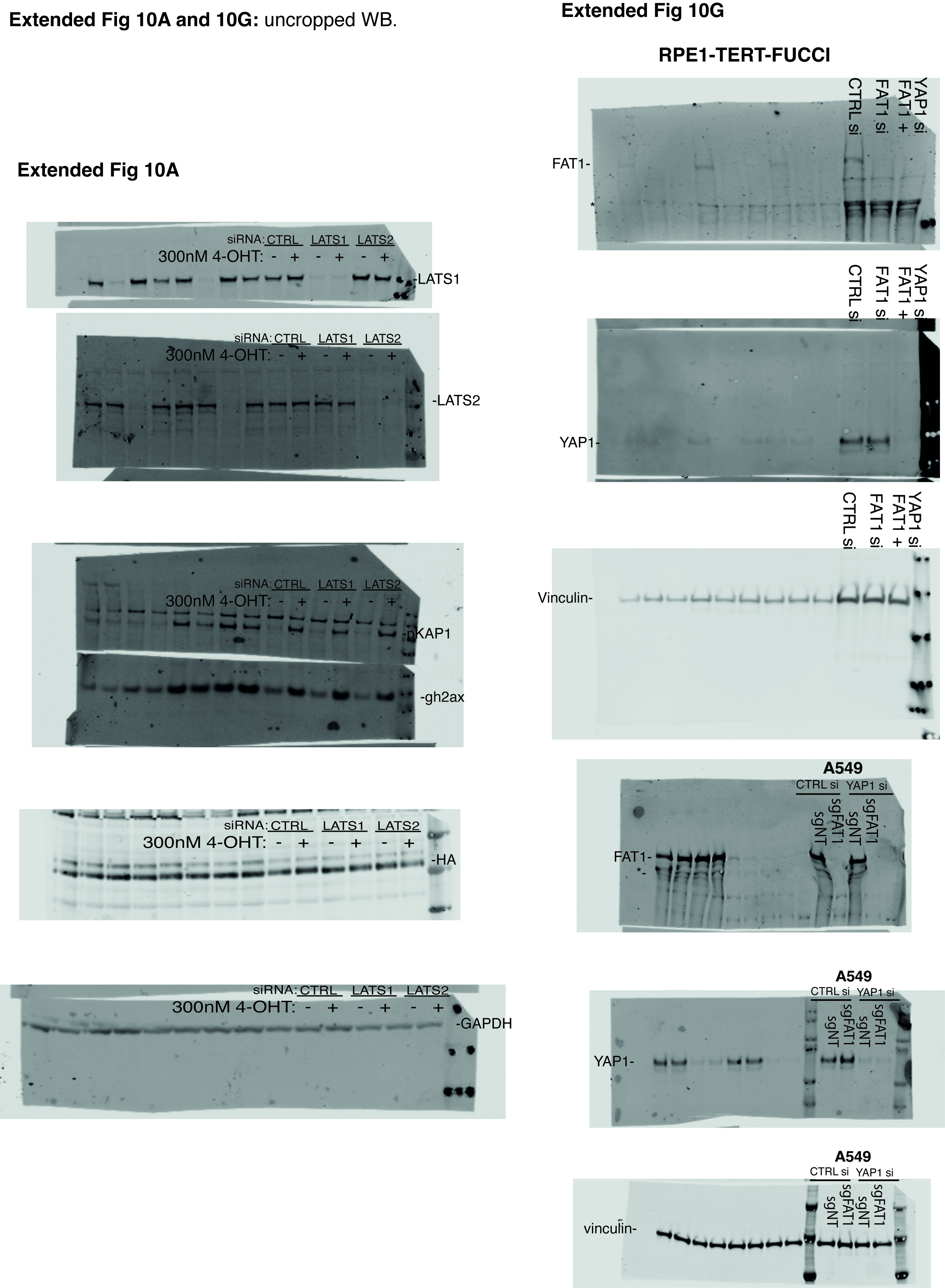

Supplement: Supplementary file 33 — Unprocessed gels or blots. [file 41556_2024_1558_MOESM33_ESM.tif]
